# Supplementary material for: Cohesin contributes to transcriptional repression of stage‐specific genes in the human malaria parasite
Source: EMBO Rep. 2023 Aug 18;24(10):e57090. doi: 10.15252/embr.202357090 (PMC10561359; doi:10.15252/embr.202357090)
Supplement: Supplementary file 1 — Expanded View Figures PDF [file EMBR-24-e57090-s003.pdf]

Expanded View Figures

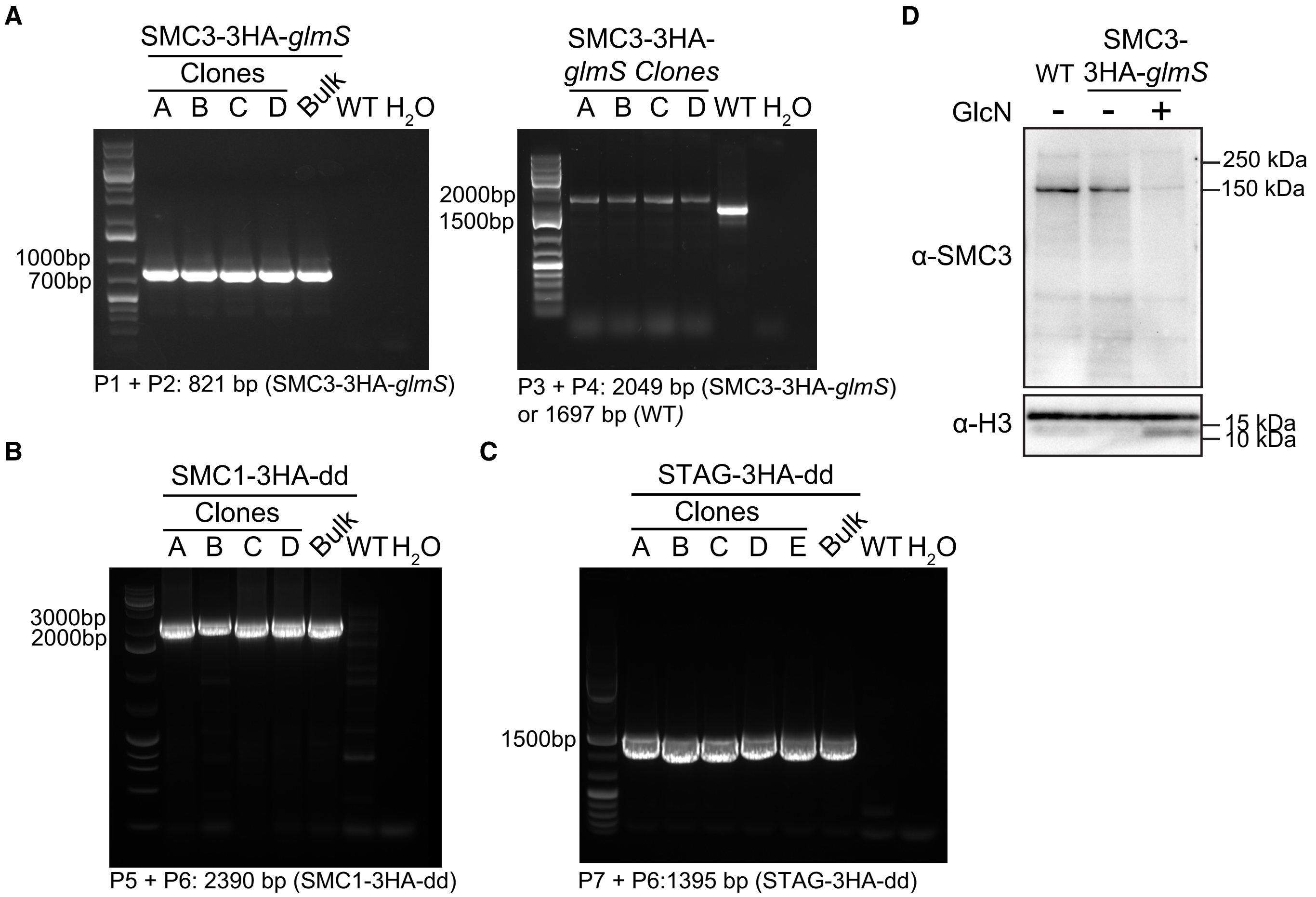

Figure EV1. Validation of strains and SMC3 antibody.

A DNA gels showing PCR validation of the SMC3-3HA-*glmS* strain with the indicated primers (Dataset EV22) shows integration of the 3HA-*glmS*-encoding sequence at the 3′ end of the endogenous *smc3* gene in the bulk transfection parasite population, as well as clones used in this study. No genomic DNA (H_2O) and genomic DNA from WT parasites (WT) are used as controls. DNA size is indicated with a ladder at the left side of each gel, and expected band sizes are indicated at the bottom of each gel.

B,C DNA gels showing PCR validation of the SMC1-3HA-dd (B) and STAG-3HA-dd (C) strains. PCR with the indicated primers (Dataset EV22) shows integration of the 3HA-dd-encoding sequence at the 3′ end of the endogenous *smc1* and *stag* genes in the bulk transfection parasite population and clones. No genomic DNA (H_2O) and genomic DNA from WT parasites (WT) are used as controls. DNA size is indicated with a ladder at the left side of each gel, and expected band sizes are indicated at the bottom of each gel.

D Western blot analysis of nuclear extracts from synchronous clonal populations of WT and SMC3-3HA-*glmS* trophozoite parasites in the absence (−) or presence (+) of glucosamine (GlcN). SMC3 is detected with an in-house generated anti-SMC3 antibody. An antibody against histone H3 is used as a loading control. Molecular weights are shown to the right.

© 2023 The Authors

Figure EV2. Extended SMC3-3HA-glmS ChIP-seq analysis.

A Venn diagrams showing overlap between significant ChIP-seq peaks called with macs2 (q-value < 0.05, Dataset EV4) from clones A and B at 12, 24, and 36 hpi.

B Circos plots of ChIP-seq data from clone B showing genome-wide SMC3 binding across the IDC. For 12 (blue), 24 (coral), and 36 (green) hpi, the 14 chromosomes are represented circularly by the outer gray bars, with chromosome number indicated in roman numerals and chromosome distances (Mbp) indicated in Arabic numerals. Enrichment ratio (ChIP/input) is shown as average reads per million (RPM) over bins of 1,000 nucleotides. The maximum y-axis value is 50 for 12 and 24 hpi and 80 for 36 hpi. Centromeric regions are represented by red bars in the innermost circle.

C Zoomed-in view of SMC3 ChIP-seq data from clone B corresponding to chromosome 4 (604,345–709,167 bp), including the centromere (represented with dark red line below the x-axis). For 12 (blue), 24 (coral), and 36 (green) hpi, the y-axis is enrichment (ChIP/Input), with vertical lines below representing significant peaks obtained from peak calling algorithm macs2 (present in both clones, q-value < 0.05). The x-axis is DNA sequence, with genes represented by black boxes indented to delineate introns and labeled with white arrowheads to indicate transcription direction.

D Plot comparing SMC3 peak enrichment [$\log_2$ (ChIP/Input)] in regions between convergent (“Converg.”) and divergent (“Diverg.”) genes at 12, 24, and 36 hpi (defined in Dataset EV6). Data shown are for consensus peaks between clone A and B that were called with macs2 (q-value < 0.05, Dataset EV4). Center black dot, median; central vertical line, standard deviation.

© 2023 The Authors

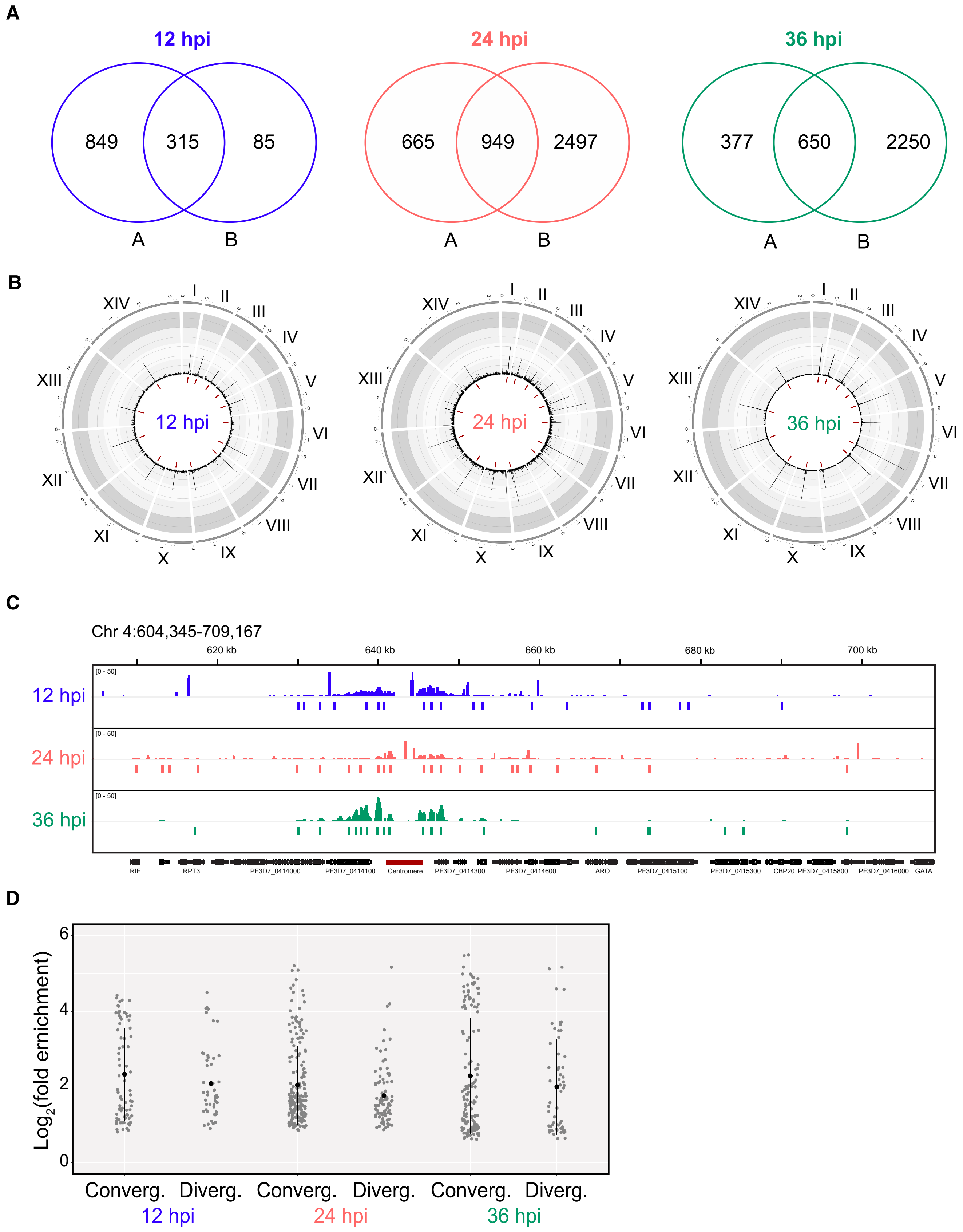
A
12 hpi
849
315
85
A
B
24 hpi
665
949
2497
A
B
36 hpi
377
650
2250
A
B
B
12 hpi
24 hpi
36 hpi
I
II
III
IV
V
VI
VII
VIII
IX
X
XI
XII
XIII
XIV
C
Chr 4:604,345-709,167
620 kb
640 kb
660 kb
680 kb
700 kb
12 hpi
24 hpi
36 hpi
[0 - 50]
RIF
RPT3
PF3D7_0414000
PF3D7_0414100
Centromere
PF3D7_0414300
PF3D7_0414600
ARO
PF3D7_0415100
PF3D7_0415300
CBP20
PF3D7_0415800
PF3D7_0416000
GATA
D
Log2(fold ernichment)
0
2
4
6
Converg.
Diverg.
Converg.
Diverg.
Converg.
Diverg.
12 hpi
24 hpi
36 hpi

Figure EV2.

Figure EV3. Analysis of SMC3-3HA-glmS knockdown.

A Western blot analysis of nuclear extracts from a synchronous clonal population of SMC3-3HA-*glmS* ring stage parasites in the absence (−) or presence (+) of glucosamine (GlcN) for 48 and 96 h (one and two IDC cycles, respectively). SMC3-3HA is detected with an anti-HA antibody. An antibody against histone H3 is used as a control. Molecular weights are shown to the right.

B RNA-seq of a WT and SMC3-3HA-*glmS* clone shows *smc3* transcript levels (FPKM) at 12, 24, and 36 hpi in the absence of glucosamine. Circles represent technical replicates of WT parasites and squares represent technical replicates of SMC3-3HA-*glmS* parasites. Asterisk indicates significance ($P < 0.05$).

C Giemsa-stained synchronous, clonal WT and SMC3-3HA-*glmS* parasite cultures in the absence (−GlcN) or presence (+GlcN) of glucosamine at the time points harvested for RNA-seq: 12, 24, and 36 hpi. Scale bar equals 10 μm.

D Cell cycle progression (hours post invasion on *x*-axis) estimation of synchronous, clonal WT and SMC3-3HA-*glmS* populations in the absence or presence of glucosamine (GlcN). RNA-seq data from synchronized parasites harvested at 12 (blue), 24 (coral), and 36 (green) hpi were compared to microarray data from (Data ref: Bozdech *et al*, 2003a; Bozdech *et al*, 2003b) as in (Lemieux *et al*, 2009) to determine the approximate time point in the IDC (*x*-axis). Replicates are represented with filled (−GlcN) or empty (+GlcN) circles.

© 2023 The Authors

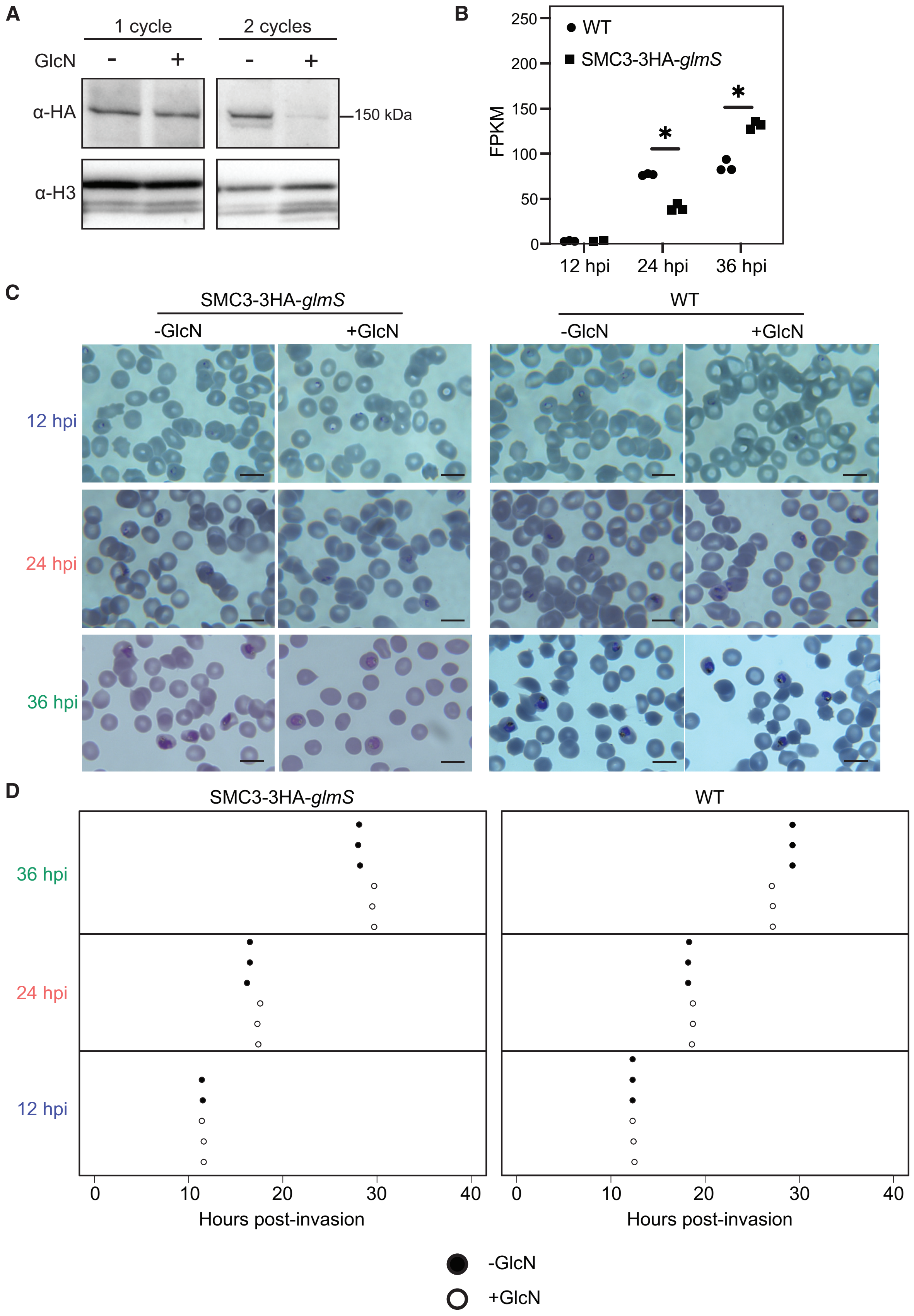
A
1 cycle
2 cycles
GlcN
-
+
-
+
α-HA
α-H3
150 kDa
B
FPKM
WT
SMC3-3HA-glmS
250
200
150
100
50
0
*
*
12 hpi
24 hpi
36 hpi
C
SMC3-3HA-glmS
WT
-GlcN
+GlcN
-GlcN
+GlcN
12 hpi
24 hpi
36 hpi
D
SMC3-3HA-glmS
WT
36 hpi
24 hpi
12 hpi
0
10
20
30
40
Hours post-invasion
-GlcN
+GlcN

Figure EV3.

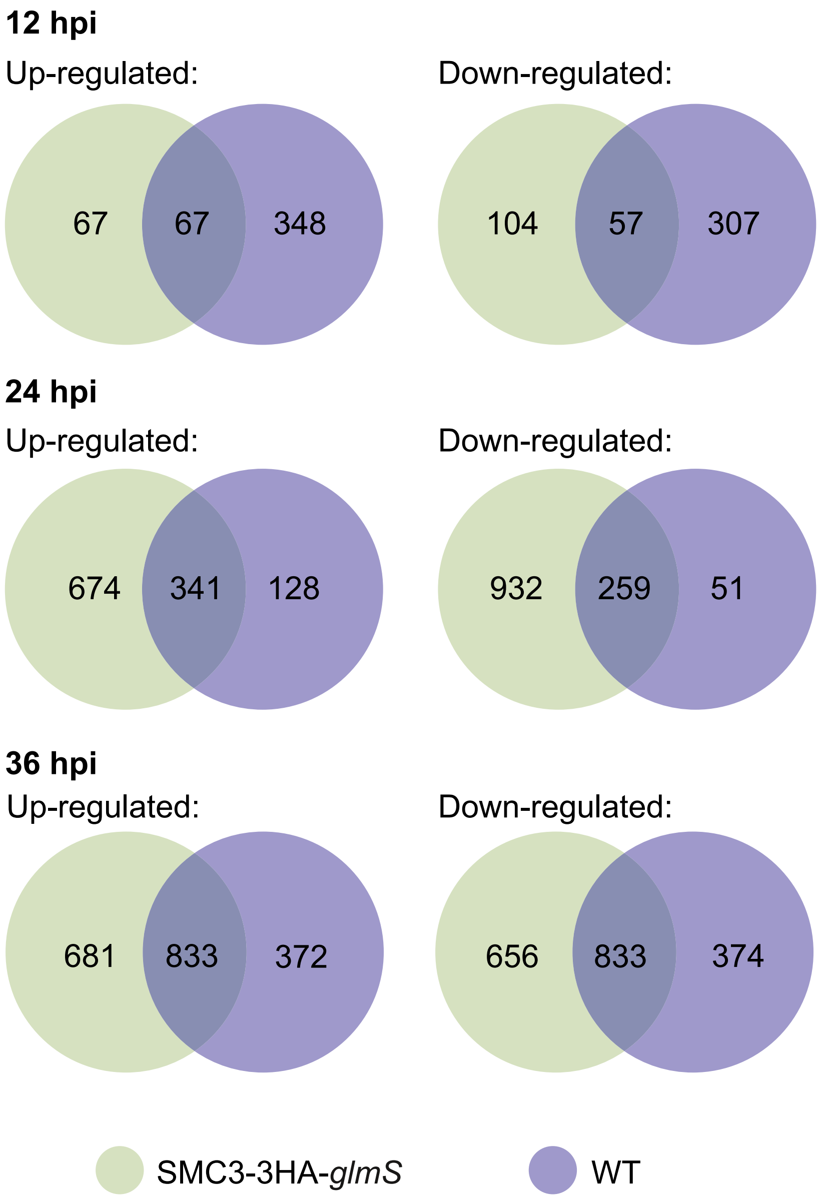

Figure EV4. Strategy for determining expression changes due to SMC3-3HA-glmS knockdown versus glucosamine treatment.

Venn diagram showing the number of unique or shared significantly up- or downregulated genes after two cycles of glucosamine treatment in synchronous, clonal populations of SMC3-3HA-glmS (green) and WT (purple) parasites at 12, 24, and 36 hpi.

© 2023 The Authors

Figure EV5. SMC3 ChIP-seq analysis at invasion- or egress-related genes.

A Metagene plot showing average SMC3 enrichment (*y*-axis = ChIP/Input) in clonal SMC3-3HA-*glmS* parasites at 12 hpi from 1.5 kb upstream of the transcription start site (TSS) to 1.5 kb downstream of the transcription end site (TES) for genes that are significantly down- (gray) or upregulated (black) upon SMC3 knockdown. One replicate (clone B) was used for the SMC3 ChIP-seq. Dashed line represents a ChIP/Input ratio of 1.

B Metagene plot for a control ChIP-seq experiment (Data ref: Baumgarten *et al*, 2022a; Baumgarten *et al*, 2022b) showing average anti-HA enrichment (*y*-axis = ChIP/Input) in WT parasites at 12 hpi from 1.5 kb upstream of the transcription start site (TSS) to 1.5 kb downstream of the transcription end site (TES) for genes that are significantly down- (gray) or upregulated (black) upon SMC3 knockdown. One replicate was used for the anti-HA ChIP-seq. Dashed line represents a ChIP/Input ratio of 1.

C Metagene plot showing average SMC3 enrichment (*y*-axis = ChIP/Input) in clonal SMC3-3HA-*glmS* parasites at 12 (blue), 24 (coral), and 36 hpi (green) from 1.5 kb upstream of the transcription start site (TSS) to 1.5 kb downstream of the transcription end site (TES) for genes that are significantly upregulated upon SMC3 knockdown at 12 hpi. One replicate (clone B) was used for the SMC3 ChIP-seq. Dashed line represents a ChIP/Input ratio of 1.

D Metagene plot showing average SMC3 enrichment (*y*-axis = ChIP/Input) in clonal SMC3-3HA-*glmS* parasites at 12 (blue), 24 (coral), and 36 hpi (green) from 1.5 kb upstream of the transcription start site (TSS) to 1.5 kb downstream of the transcription end site (TES) for invasion-related genes, as defined in Hu *et al* (2010). One replicate (clone B) was used for the SMC3 ChIP-seq. Dashed line represents a ChIP/Input ratio of 1.

E Metagene plot showing average SMC3 enrichment (*y*-axis = ChIP/Input) in clonal SMC3-3HA-*glmS* parasites at 12 (blue), 24 (coral), and 36 hpi (green) from 1.5 kb upstream of the transcription start site (TSS) to 1.5 kb downstream of the transcription end site (TES) for randomly chosen genes that reach peak transcription in early-stage parasites. One replicate (clone A) was used for the SMC3 ChIP-seq. Dashed line represents a ChIP/Input ratio of 1.

F Metagene plot showing average SMC3 enrichment (*y*-axis = ChIP/Input) in clonal SMC3-3HA-*glmS* parasites at 12 (blue), 24 (coral), and 36 hpi (green) from 1.5 kb upstream of the transcription start site (TSS) to 1.5 kb downstream of the transcription end site (TES) for genes that reach peak expression in gametocytes. One replicate (clone A) was used for the SMC3 ChIP-seq. Dashed line represents a ChIP/Input ratio of 1.

G Metagene plot for a control ChIP-seq experiment (Data ref: Baumgarten *et al*, 2022a; Baugarten *et al*, 2022b) showing average anti-HA enrichment (*y*-axis = ChIP/Input) in WT parasites at 12 hpi from 1.5 kb upstream of the transcription start site (TSS) to 1.5 kb downstream of the transcription end site (TES) for invasion-related genes (black) as defined in Hu *et al* (2010), randomly chosen genes that reach peak transcription in early-stage parasites (gray), and genes that reach peak expression in gametocytes (gold). One replicate was used for the anti-HA ChIP-seq. Dashed line represents a ChIP/Input ratio of 1.

H ChIP-seq data showing enrichment of SMC3 (*y*-axis = ChIP/Input) at 12 (blue), 24 (coral), and 36 (green) hpi in clonal SMC3-3HA-*glmS* parasites at the *rhoptry-associated protein 2* (*rap2*, PF3D7_0501600) and the *glideosome-associated protein 45* (*gap45*, PF3D7_1222700) gene loci. The *x*-axis is DNA sequence, with the gene represented by a black box with white arrowheads to indicate transcription direction. One replicate (clone B) was used for ChIP-seq. ATAC-seq data from closely corresponding time points (15, 25, and 35 hpi) from Data ref: Toenhake *et al* (2018a); Toenhake *et al* (2018b) are shown in gray, with the *y*-axis representing ATAC-seq (RPM)/gDNA (RPM).

I ChIP-seq data showing enrichment of SMC3 (*y*-axis = ChIP/Input) at 12 (blue), 24 (coral), and 36 (green) hpi in clonal SMC3-3HA-*glmS* parasites at the *merozoite surface protein 1* (*msp1*, PF3D7_0930300) and the *merozoite surface protein 9* (*msp9*, PF3D7_1228600) gene loci. The *x*-axis is DNA sequence, with the gene represented by a black box with white arrowheads to indicate transcription direction. Two biological replicates (clones A and B) were used for ChIP-seq.

© 2023 The Authors

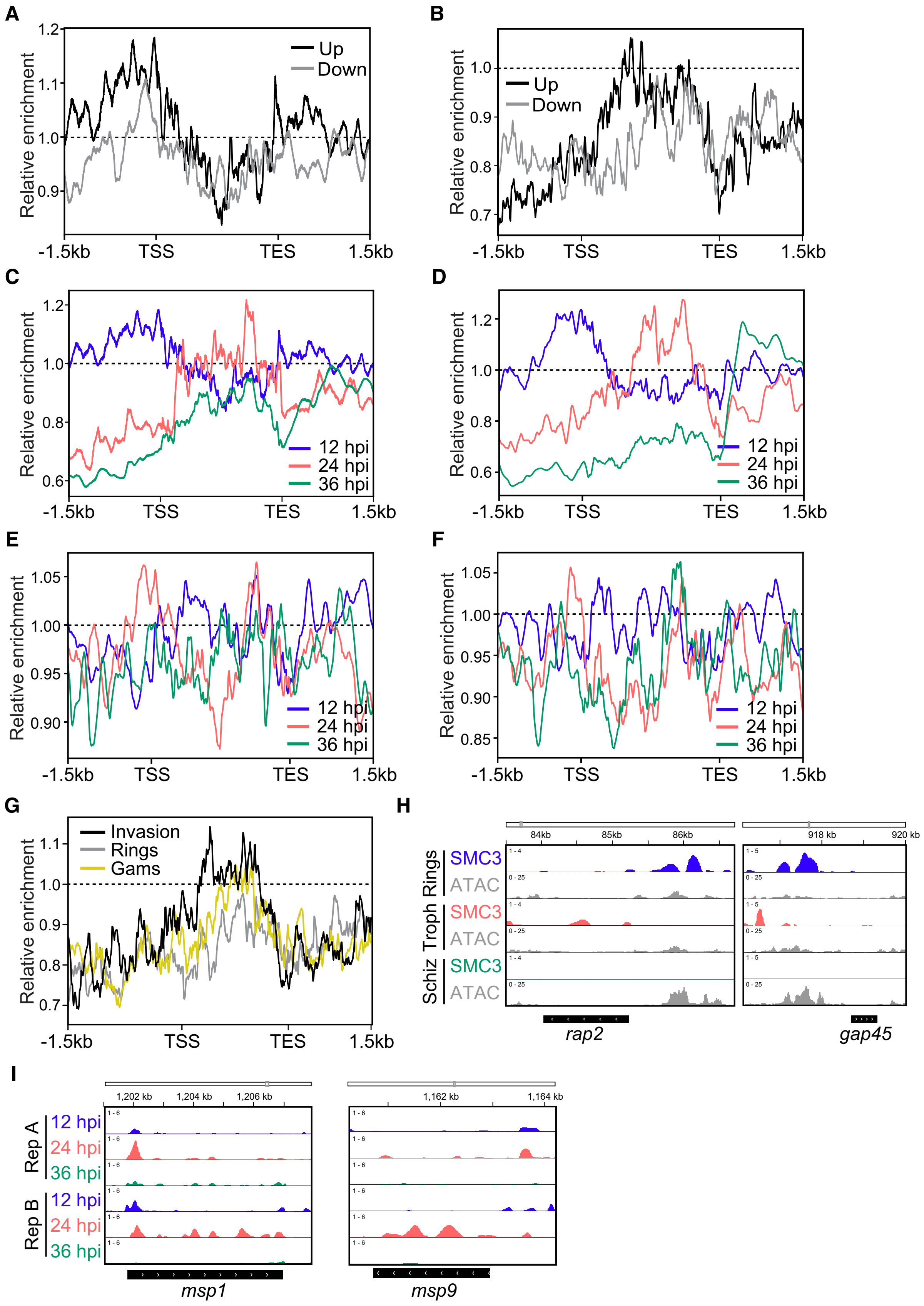

Figure EV5.

 © 2023 The Authors
