# Supplementary material for: Cohesin contributes to transcriptional repression of stage‐specific genes in the human malaria parasite
Source: EMBO Rep. 2023 Aug 18;24(10):e57090. doi: 10.15252/embr.202357090 (PMC10561359; doi:10.15252/embr.202357090)
Supplement: Supplementary file 24 — PDF+ [file EMBR-24-e57090-s009.pdf]

Article

SOURCE DATA TRANSPARENT PROCESS OPEN ACCESS

EMBO reports

Cohesin contributes to transcriptional repression of stage-specific genes in the human malaria parasite

Catarina Rosa[1,2], Parul Singh[1], Patty Chen[1], Ameya Sinha[3,4], Aurélie Claës[1], Peter R Preiser[3,4], Peter C Dedon[4,5], Sebastian Baumgarten[6], Artur Scherf[1] & Jessica M Bryant[1,*]

Abstract

The complex life cycle of the human malaria parasite, *Plasmodium falciparum*, is driven by specific transcriptional programs, but it is unclear how most genes are activated or silenced at specific times. There is an association between transcription and spatial organization; however, the molecular mechanisms behind genome organization are unclear. While *P. falciparum* lacks key genome-organizing proteins found in metazoans, it has all core components of the cohesin complex. To investigate the role of cohesin in *P. falciparum*, we functionally characterize the cohesin subunit Structural Maintenance of Chromosomes protein 3 (SMC3). SMC3 knockdown during early stages of the intraerythrocytic developmental cycle (IDC) upregulates a subset of genes involved in erythrocyte egress and invasion, which are normally expressed at later stages. ChIP-seq analyses reveal that during the IDC, SMC3 enrichment at the promoter regions of these genes inversely correlates with gene expression and chromatin accessibility. These data suggest that SMC3 binding contributes to the repression of specific genes until their appropriate time of expression, revealing a new mode of stage-specific gene repression in *P. falciparum*.

Keywords chromatin; cohesin; *Plasmodium falciparum*; transcription
Subject Categories Chromatin, Transcription & Genomics; Molecular Biology of Disease
DOI 10.15252/embr.202357090 | Received 28 February 2023 | Revised 18 July 2023 | Accepted 21 July 2023 | Published online 18 August 2023
EMBO Reports (2023) 24: e57090

Introduction

The most virulent human malaria parasite, *Plasmodium falciparum*, has a significant impact on human health in endemic regions (World Health Organization, 2020). The approximately 48-h intraerythrocytic developmental cycle (IDC) takes place in the human blood and is responsible for all clinical symptoms of malaria. During the IDC, each parasite replicates by schizogony, giving rise to up to 36 daughter cells that egress out of the red blood cell (RBC) and begin a new round of infection (Cowman *et al*, 2016). Underlying parasite development across the IDC is a highly coordinated gene expression program in which transcription of most genes peaks when the corresponding protein is required (Bozdech *et al*, 2003b; Painter *et al*, 2018b). Since the major limiting step for gene expression is transcription initiation (Caro *et al*, 2014), one possibility is that expression patterns result from a precisely timed production and/or binding of sequence-specific transcription factors (TFs). While recent studies of chromatin accessibility show evidence for dynamic exposure of potential transcription factor binding sites upstream of genes, the *P. falciparum* genome encodes few sequence-specific TFs compared to other eukaryotes, accounting for < 1% of the protein-coding genes (Balaji *et al*, 2005; Campbell *et al*, 2010; Toenhake *et al*, 2018b).

Most of the *P. falciparum* genome is in a transcriptionally permissive, euchromatic state, with histone acetylation and deacetylation being the main predictors of gene activation or repression, respectively (Salcedo-Amaya *et al*, 2009; Trelle *et al*, 2009). Exceptions to this rule include multigene families encoding variant surface antigens, which are uniquely heterochromatinized via heterochromatin protein 1 (HP1) and form clusters at the nuclear periphery (Ralph *et al*, 2005; Lopez-Rubio *et al*, 2009). The recent application of genome-wide chromosome conformation capture techniques (Hi-C) confirmed close association of these multigene families, the clustering of centromeres and telomeres at opposite sides of the nucleus, and co-localization of active ribosomal DNA (rDNA) genes (Ay *et al*, 2014; Bunnik *et al*, 2019). In addition to the strong clustering of HP1-enriched multigene families and highly transcribed rDNA units, genes with similar expression profiles were also found to associate in a spatiotemporal manner during the IDC (Ay *et al*, 2014). Indeed, certain gene families appear to change their localization within the nucleus between the IDC (rings, trophozoites, and schizonts), the transmission from human to mosquito (early and late gametocytes), and from mosquito to human (sporozoites; Bunnik *et al*, 2018).

Although this growing body of evidence shows that specific genes and genomic features associate at specific times in the

1 Institut Pasteur, Université Paris Cité, INSERM U1201, CNRS EMR9195, Biology of Host-Parasite Interactions Unit, Paris, France
2 Sorbonne Université, Collège Doctoral Complexité du Vivant ED515, Paris, France
3 School of Biological Sciences, Nanyang Technological University, Singapore, Singapore
4 Antimicrobial Resistance Interdisciplinary Research Group, Singapore-MIT Alliance for Research and Technology, Singapore, Singapore
5 Department of Biological Engineering, Massachusetts Institute of Technology, Cambridge, MA, USA
6 Institut Pasteur, Université Paris Cité, Parasite RNA Biology Group, Paris, France
*Corresponding author. Tel: +33 (0)1 40 66 96 22; E-mail: jessica.bryant@pasteur.fr

© 2023 The Authors. Published under the terms of the CC BY 4.0 license

P. falciparum life cycle, the factors responsible for this organization are largely unknown. Protein factors including actin and HP1 were shown to play a role in the organization and transcriptional regulation of the *var* multigene family (Ralph *et al*, 2005; Lopez-Rubio *et al*, 2009; Zhang *et al*, 2011). More recently, an architectural factor, the high-mobility-group-box protein 1 (*Pf*HMGB1) was found to play a role in the nuclear organization of centromeres, and knockdown led to defects in *var* gene transcription (Lu *et al*, 2021). However, architectural factors linking chromosomal organization to the strict spatio-temporal transcriptional regulation of HP1-independent genes remain to be uncovered.

Although *P. falciparum* lacks lamins and CCCTC-binding factor (CTCF)—key genome organizing proteins in metazoans (Batsios *et al*, 2012; Heger *et al*, 2012)—it encodes the functionally uncharacterized putative orthologues of the core components of the cohesin complex: Structural Maintenance of Chromosomes protein 1 (SMC1, PF3D7_1130700), SMC3 (PF3D7_0414000), and an α-kleisin subunit (RAD21; PF3D7_1440100; Gardner *et al*, 2002). Among eukaryotes investigated, cohesin is a multiprotein complex that performs multiple different functions that primarily rely on its ability to topologically entrap strands of DNA (reviewed in Dorsett & Ström, 2012; Uhlmann, 2016; Perea-Resa *et al*, 2021). SMC1 and SMC3 each contain a hinge domain, which facilitates dimerization between the two proteins, and an ATPase head domain (Fig 1A). Association with RAD21 at the SMC1/3 head domains results in a ring-like structure (Fig 1A) that can both entrap DNA (Gligoris *et al*, 2014; Huis in 't Veld *et al*, 2014) and extrude DNA loops (Davidson *et al*, 2019; Kim *et al*, 2019).

The most well-characterized role of cohesin is in holding replicated sister chromatids together to ensure faithful chromosome segregation during cell division (Michaelis *et al*, 1997). Cohesin is loaded onto chromosomes during G_1 or S phase, but in early mitosis, most of it is removed except for at centromeric and pericentromeric regions. This final pool is removed at the onset of anaphase to facilitate chromatid separation (reviewed in Peters & Nishiyama, 2012; Mirkovic & Oliveira, 2017). In the IDC of *P. falciparum*, asexual replication is accomplished through endocyclic schizogony, during which asynchronous rounds of DNA replication and mitosis lead to multinucleated cells, all in the absence of chromosome condensation (Fig 1B). Schizogony culminates with a final round of nuclear division before cytokinesis (Rudlaff *et al*, 2020; Klaus *et al*, 2022). While much recent progress has been made in elucidating the mechanisms behind this unique cell division, many questions remain.

In contrast to mitosis, cohesin binding during G_1 phase or in non-dividing cells was found to be more dynamic (Gerlich *et al*, 2006; Eichinger *et al*, 2013). In fact, in more recent years, the cohesin complex and the regulatory proteins that control the loading and unloading of the complex to DNA were found to play a role in shaping chromosomal architecture and thus, transcription, during interphase. In mammalian cells, cohesin and CTCF are often found at the boundaries of topologically associating domains (TADs; Dixon *et al*, 2012; Rao *et al*, 2017; Wutz *et al*, 2017; Nuebler *et al*, 2018). A TAD is a region of the genome that preferentially interacts with itself in comparison with the rest of the genome (reviewed in Dekker & Heard, 2015). Importantly, TADs have emerged as functional structures involved in the regulation of cell type- and developmental stage-specific transcriptional programs, most likely via the correct pairing of enhancers with promoters (reviewed in Dixon *et al*, 2016; Perea-Resa *et al*, 2021). While the *P. falciparum* genome is not organized into TADs, as they are defined in metazoans, it does feature long-range inter- and intra-chromosomal interactions that are involved in transcriptional control (Zhang *et al*, 2011; Ay *et al*, 2014; Bunnik *et al*, 2019).

In *Plasmodium*, the physical association of SMC1, SMC3, and a protein containing the Rad21/Rec8-like N-terminal domain has been described (Hillier *et al*, 2019). Recently, a preliminary characterization of *Pf*SMC3 was carried out using an antibody generated in-house (Batugedara *et al*, 2020). Chromatin immunoprecipitation and sequencing (ChIP-seq) in trophozoites revealed that SMC3 was enriched at centromeric regions (Batugedara *et al*, 2020). In the present study, we use genome editing, mass spectrometry, and ChIP- and RNA-seq to functionally characterize the cohesin subunit SMC3 in interphase transcriptional regulation during the IDC. We show that while SMC3 is constantly present at centromeres across the IDC (Batugedara *et al*, 2020), it binds dynamically to the promoters of a specific subset of genes that are upregulated in its absence. Our findings represent a new mode of transcriptional repression in *P. falciparum*.

Results

SMC3 is expressed across the IDC and localizes to HP1-independent nuclear foci

In the *P. falciparum* genome, three putative core cohesin subunits have been annotated: SMC1 (PF3D7_1130700), SMC3 (PF3D7_0414000),

Figure 1. SMC3 is part of a conserved cohesin complex in *P. falciparum*. ►

A Cohesin complex subunits annotated in *P. falciparum* (Gardner *et al*, 2002). The hinge and ATPase domains of SMC1 and SMC3 are indicated. Image prepared with BioRender.com.

B Schematic of *P. falciparum* intraerythrocytic developmental cycle (IDC). Yellow, approximate timing of DNA replication; Gray, approximate duration of schizogony (modified from Ganter *et al*, 2017; Matthews *et al*, 2018). Time points in this study – 12 hpi (ring), 24 hpi (trophozoite), and 36 hpi (schizont) – are indicated.

C Alignment of *P. falciparum* (*Pf*) SMC3 (*Pf*SMC3) with SMC3 protein sequences in *H. sapiens* (*Hs*), *D. melanogaster* (*Dm*), *S. pombe* (*Sp*), *S. cerevisiae* (*Sc*), and *A. thaliana* (*At*). A schematic of *Pf*SMC3 domain architecture is shown above. Coiled-coil domains are in yellow, low complexity regions are in blue, and other structured domains are annotated and in green. Sequence consensus ("Cons") is indicated by the gray bar with colors representing regions of 100% agreement between the aligned sequences. Image prepared with Geneious Prime 2020.0.3.

D, E Western blot analysis with an anti-HA antibody or in-house-generated anti-SMC3 antibody (indicated to the left of each blot) of nuclear extracts from synchronous SMC1-3HA-dd (D) or STAG-3HA-dd (E) late-stage parasites before (input, "In") and after (flow-through, "FT") immunoprecipitation with an antibody against HA ("α-HA IP"). An antibody against histone H3 is used as a control. Immunoprecipitation with IgG ("IgG IP") was used as a control in the right panel of (E). Molecular weights are shown to the right. SMC1-3HA-dd has a predicted molecular weight of approximately 230 kDa and STAG-3HA-dd has a predicted molecular weight of approximately 298 kDa (of which 3.3 and 11.9 kDa correspond to the 3HA tag and the ddFKBP, respectively).

Source data are available online for this figure.

 © 2023 The Authors

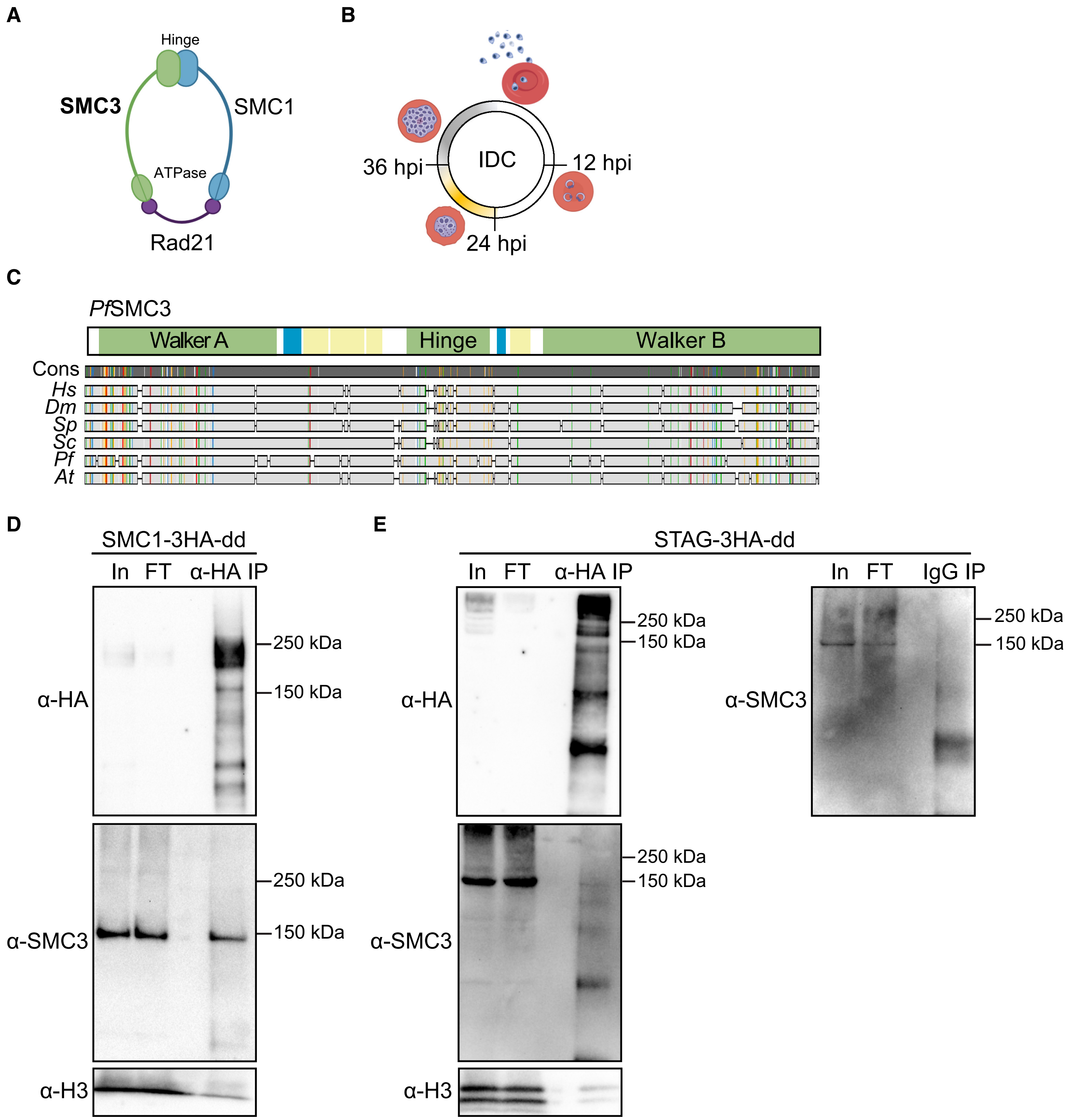

Figure 1.

and a protein with the N-terminal Rad21/Rec8 domain (PF3D7_1440100; Fig 1A). A comparative sequence analysis showed that, of these three subunits, *Pf*SMC3 shares the highest sequence similarly and identity to its orthologues in *H. sapiens*, *D. melanogaster*, *S. cerevisiae*, *S. pombe*, and *A. thaliana* (Fig 1C). A Pfam domain analysis (Mistry *et al*, 2021) showed an overall conserved domain architecture: an N-terminal Walker A motif-containing domain, a central hinge domain, and a C-terminal Walker B motif-containing domain (Fig 1C). Given the conserved nature of *Pf*SMC3, we decided to investigate its function *in vivo*.

We used CRISPR/Cas9 genome editing (Ghorbal *et al*, 2014) to add a 3× hemagglutinin (3HA) epitope tag-encoding sequence followed by a *glmS* ribozyme sequence at the 3′ end of *smc3* (SMC3-3HA-*glmS*; Fig EV1A), which allows for inducible knockdown (Prommana *et al*, 2013). Immunoprecipitation followed by liquid chromatography-mass spectrometry (IP LC–MS/MS) of SMC3-3HA

© 2023 The Authors

confirmed the interaction of SMC1, SMC3, and RAD21 previously reported in *P. falciparum* (Hillier *et al*, 2019; Batugedara *et al*, 2020; Dataset EV1). A Stromal Antigen ("STAG") domain-containing protein (PF3D7_1456500) was also enriched in the SMC3-3HA IP LC–MS/MS, suggesting that a fourth cohesin subunit (STAG1/2 in *H. sapiens* and Scc3 in *S. cerevisiae*) is present in the *P. falciparum* cohesin complex (Dataset EV1). We used selection-linked integration genome editing (Birnbaum *et al*, 2017) to add a 3HA epitope tag-encoding sequence followed by a destabilization domain-encoding sequence at the 3′ end of *smc1* (SMC1-3HA-dd) and *stag* (STAG-3HA-dd; Fig EV1B and C). Using an in-house generated anti-SMC3 antibody (Fig EV1D), interaction of SMC1 and STAG with SMC3 was confirmed with co-immunoprecipitation (Fig 1D and E), suggesting that these subunits exist in a core cohesin complex similar to that found in other eukaryotes.

Western blot analysis of a synchronous bulk population of SMC3-3HA-*glmS* parasites showed that SMC3 is expressed across the IDC but increases in abundance from ring to schizont stage (Fig 2A). The presence of SMC3 in both ring and trophozoite stages suggests that cohesin plays a role in interphase parasites (i.e. outside schizogony) and perhaps even before the onset of S phase, which is believed to take place after 24 hpi (Arnot *et al*, 2011; Ganter *et al*, 2017; Stanojcic *et al*, 2017; Fig 1B). Immunofluorescence assay (IFA) corroborated the nuclear localization, revealing a focus of SMC3-3HA at the nuclear periphery in trophozoite and schizont stages (Fig 2B). While these foci are reminiscent of the heterochromatic *var* gene clusters at the nuclear periphery, no co-localization was observed between SMC3 and HP1 foci in trophozoite stage (Fig 2C). It was not possible to detect SMC3 in ring stage and early trophozoite parasites with IFA, possibly due to the low abundance of the protein at this stage.

SMC3 binds stably to centromeres, but dynamically to other genes across the IDC

To determine the genome-wide binding pattern of SMC3 across the IDC, ChIP-seq was performed in synchronous clonal populations (clones A and B) of SMC3-3HA-*glmS* parasites at 12 (ring), 24 (trophozoite), and 36 (schizont) hours post invasion (hpi). Using the macs2 peak calling algorithm (Zhang *et al*, 2008), we obtained 1,164, 1,614, and 1,027 significant peaks at 12, 24, and 36 hpi, respectively (Dataset EV2), for clone A and 400, 3,446, and 2,900 significant peaks at 12, 24, and 36 hpi, respectively (Dataset EV3), for clone B. There was significant overlap ($P < 0.01$) between significant peaks called in both clones for each time point, and we used this cohort of shared peaks for further analysis (Dataset EV4, Fig EV2A). No significant overlap was seen between consensus SMC3 peaks and peaks called for a negative control anti-HA ChIP performed in a wild-type (WT) strain at the same time point (Data ref: Baumgarten *et al*, 2022a; Baumgarten *et al*, 2022b).

Most striking was SMC3 enrichment at centromeric regions at all time points, a phenomenon that was previously reported for trophozoite stages (Batugedara *et al*, 2020; Figs 3A and B, and EV2B and C). Comparison of the SMC3 peaks with the centromeric regions defined in Hoeijmakers *et al* (2012) revealed extensive overlap (Dataset EV5). SMC3 peak enrichment in centromeric regions was significantly higher than that of the peaks associated with the rest of the genome at 12, 24, and 36 hpi ($P < 0.0001$; Fig 3C). While quantification of the SMC3 peaks showed the largest enrichment in the centromeric and pericentromeric regions, there were significant SMC3 peaks across other genomic locations at all time points (Dataset EV4, Figs 3B and EV2C). As was seen for cohesin in *S. cerevisiae* (Lengronne *et al*, 2004), more SMC3 peaks were found in regions between convergent genes versus divergent genes or head-to-tail genes (Dataset EV6). SMC3 peak enrichment values were higher in regions between convergent genes versus divergent genes, although the difference was not significant (Dataset EV6, Fig EV2D).

SMC3 peaks were found in intergenic and intragenic regions closest to 164, 625, and 437 protein coding genes at 12, 24, and 36 hpi, respectively (Dataset EV7). Of all genes within ±500 base pairs (bp) of an SMC3 peak, 83 were bound by SMC3 across all three time points (Fig 3D). In general, these genes were near centromeric regions but were otherwise functionally unrelated (Dataset EV8). However, most SMC3-bound genes showed a dynamic binding pattern, with a peak present at only one or two time points (Dataset EV7, Fig 3B and D). Gene ontology (GO) enrichment analysis showed that genes associated with SMC3 peaks at 12 hpi were not significantly represented by a specific GO term category (Dataset EV8) while genes associated with SMC3 peaks at 24 and 36 hpi were most significantly represented by biological process categories such as "obsolete pathogenesis" ($q = 9.4 \times 10^{-28}$ and 2.6×10^{-9}, respectively), "cell–cell adhesion" ($q = 4 \times 10^{-24}$ and 1.8×10^{-9}, respectively), "response to host" ($q = 1.6 \times 10^{-19}$ and 1×10^{-8}, respectively), and "antigenic variation" ($q = 1.6 \times 10^{-19}$ and 3.4×10^{-8}, respectively; Dataset EV8). These categories include many genes in common such as *var* and *rif* genes, which encode proteins that are exported to the surface of the host red blood cell to facilitate adhesion to the host microvasculature (reviewed in Scherf *et al*, 2008). Genes associated with SMC3 peaks at 24 hpi were also significantly represented by the categories "biological process involved in interaction with host" ($q = 1 \times 10^{-19}$), "movement in host environment" ($q = 3.3 \times 10^{-3}$), "exit from host" ($q = 0.026$), and "entry into host" ($q = 0.05$). These categories include genes that are involved in invasion of or egress from the red blood cell such as *ralp1* (PF3D7_0722200; Haase *et al*, 2008), *rhoph3* (PF3D7_0905400; Sherling *et al*, 2017), and *msp1* (PF3D7_0930300; O'Donnell *et al*, 2000, 2001).

While peak calling analysis is informative, the diverse functional categories of genes associated with SMC3 peaks makes it difficult to determine if SMC3 plays a specific role in transcriptional regulation or binds randomly throughout genic regions to facilitate a role in mitosis-related chromosome organization. Thus, functional analysis was required to elucidate a potential transcriptional function for SMC3 binding.

SMC3 inducible knockdown results in deregulation of genes across the IDC

To gain insight into the transcriptional role of SMC3 during the IDC, we performed an inducible knockdown of SMC3 using the *glmS* ribozyme system (Prommana *et al*, 2013). An SMC3-3HA-*glmS* clone was tightly synchronized and split, and glucosamine was added to one half for 96 h (two cell cycles), as knockdown at the protein level could not be achieved after a single cell cycle (Fig EV3A). Simultaneously, a WT clone from the parent 3D7 strain was synchronized and treated in the same way to account for transcriptional

 © 2023 The Authors

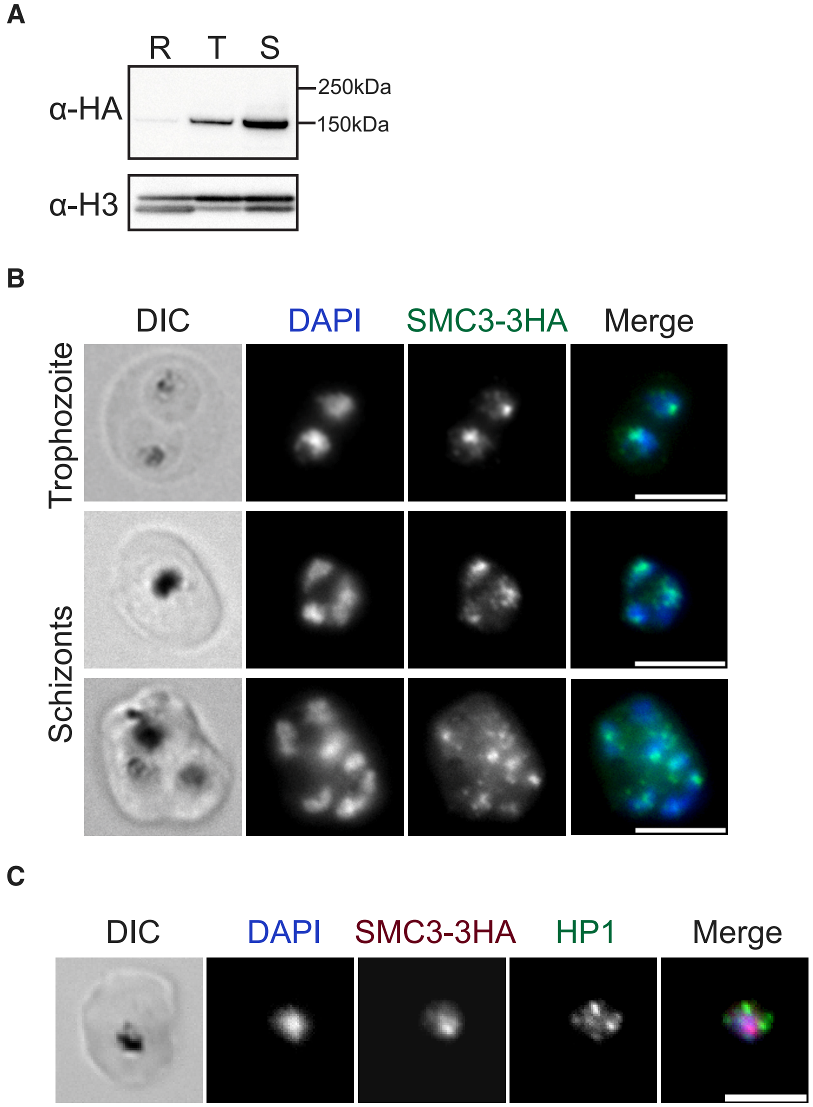

Figure 2. SMC3 is expressed across the IDC and localizes to HP1-independent nuclear foci.

A Western blot analysis of nuclear extracts of ring (R), trophozoite (T), and schizont (S) stages from a synchronous population of SMC3-3HA-*glmS* parasites. SMC3-3HA is detected with an anti-HA antibody. An antibody against histone H3 is used as a control. Molecular weights are shown to the right. SMC3-3HA has a predicted molecular weight of 147.3 kDa (of which 3.3 kDa correspond to the 3HA tag).

B, C Immunofluorescence assays of fixed RBCs infected with trophozoite or schizont stage SMC3-3HA-*glmS* parasites. DNA was stained with DAPI (blue) and SMC3-3HA was detected with anti-HA (green in B and magenta in C) antibody. HP1 was detected with anti-HP1 antibody (green in C). DIC, differential interference contrast. Scale bars equal 10 µm (B) and 5 µm (C).

Source data are available online for this figure.

changes due to the presence of glucosamine. After another round of synchronization, parasites were harvested at 12, 24, and 36 hpi, and western blot analysis revealed an SMC3-3HA knockdown at the protein level in nuclear extracts at all time points, although to a lesser extent at 36 hpi (Fig 4A). Growth curve analysis showed the SMC3 depletion did not significantly affect parasitemia over 5 days of culture (Fig 4B).

We then performed RNA-seq followed by differential expression analysis for the untreated and glucosamine-treated SMC3-3HA-*glmS* and WT parasites, which confirmed a significant knockdown of SMC3 at the transcript level in the SMC3-3HA-*glmS* parasites: 55% at 12 hpi (Dataset EV9, $q = 8.5 \times 10^{-3}$), 69% at 24 hpi (Dataset EV10, $q = 1.3 \times 10^{-39}$), and 48% at 36 hpi (Dataset EV11, $q = 4.1 \times 10^{-54}$; Fig 5A). While there was a significant difference in SMC3 transcript levels between untreated WT and SMC3-3HA-*glmS* parasites at 24 and 36 hpi (Fig EV3B), this did not seem to translate into a significant difference in SMC3 protein levels in trophozoite parasites (Fig EV1D). To determine synchronicity of untreated and glucosamine-treated strains and replicates, we performed Giemsa staining of parasites during the time course (Fig EV3C) and compared our RNA-seq data to time course microarray data from (Data ref: Bozdech *et al*, 2003a;

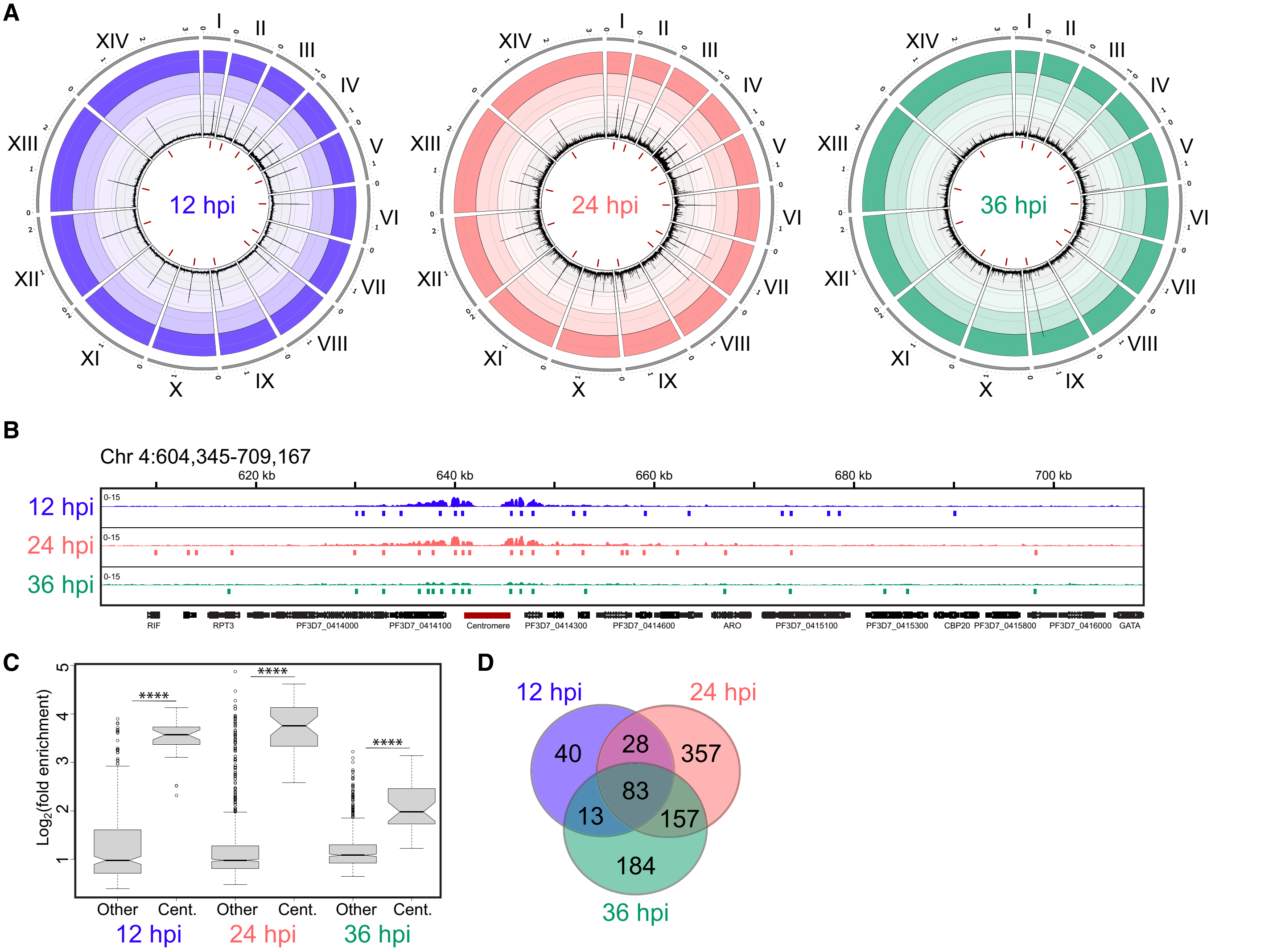

Figure 3. SMC3 binds stably to centromeres, but dynamically to other genes across the IDC.

A Circos plots of ChIP-seq data from clone A showing genome-wide SMC3 binding across the IDC. For 12 (blue), 24 (coral), and 36 (green) hpi, the 14 chromosomes are represented circularly by the outer gray bars, with chromosome number indicated in roman numerals and chromosome distances (Mbp) indicated in Arabic numerals. Enrichment ratio (ChIP/input) is shown as average reads per million (RPM) over bins of 1,000 nucleotides. The maximum *y*-axis value is 24. Centromeric regions are represented by red bars in the innermost circle.

B Zoomed-in view of SMC3 ChIP-seq data from clone A corresponding to chromosome 4 (604,345–709,167 bp), including the centromere (represented with dark red line below the *x*-axis). For 12 (blue), 24 (coral), and 36 (green) hpi, the *y*-axis is enrichment (ChIP/Input), with vertical lines below representing significant peaks obtained from peak calling algorithm macs2 (present in both clones, *q*-value < 0.05). The *x*-axis is DNA sequence, with genes represented by black boxes indented to delineate introns and labeled with white arrowheads to indicate transcription direction.

C Box plot comparing the distribution of SMC3 peak enrichment [$\log_2$ (ChIP/Input)] between centromeric (Cent.) regions and extra-centromeric (Other) regions of the genome for 12, 24, and 36 hpi. Data shown are for consensus peaks between clone A and B that were called with macs2 (*q*-value < 0.05, Dataset EV4). Center line, median; box limits, first and third quartiles; whiskers, 1.5× interquartile range. Wilcoxon test was used for statistical analysis. **** = adjusted *P*-value < 0.0001.

D Venn diagram showing overlap between SMC3 peak-associated genes at 12 (blue), 24 (coral), and 36 (green) hpi. Closest unique protein-coding genes to the SMC3-3HA consensus peaks (± 500 bp) at 12, 24, and 36 hpi are shown in Dataset EV7.

Bozdech *et al*, 2003b), as in (Lemieux *et al*, 2009; Fig EV3D). These analyses suggested tight synchronicity at each time point, showing that the morphology and “transcriptional age” of all replicates from the untreated and glucosamine-treated parasites harvested at each respective time point were within ± 3 h of each other (Fig EV3C and D).

To remove potential artifacts of glucosamine treatment, genes that were significantly up- or downregulated in the glucosamine-treated WT parasites at 12, 24, and 36 hpi (Datasets EV12–EV14, respectively) were filtered out of the datasets for significantly up- and downregulated genes in the SMC3-3HA-*glmS* parasites at the corresponding time points (Fig EV4). After filtering, 104, 932, and 656 genes were significantly downregulated and 67, 674, and 681 genes were significantly upregulated (Figs 5B and EV4, and Datasets EV15–EV17) at 12, 24, and 36 hpi, respectively, in SMC3-3HA-*glmS* parasites.

 © 2023 The Authors

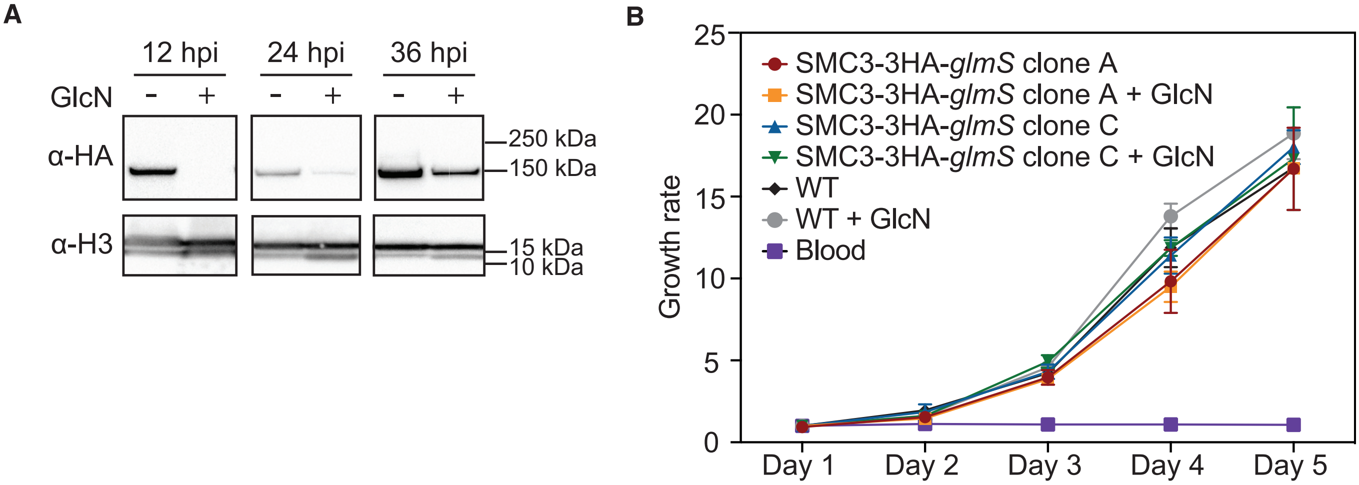

Figure 4. SMC3 inducible knockdown does not affect parasite growth.

A Western blot analysis of nuclear extracts at 12, 24, and 36 hpi from a clonal population of SMC3-3HA-*glmS* parasites in the absence (−) or presence (+) of glucosamine (GlcN). SMC3-3HA is detected with an anti-HA antibody. An antibody against histone H3 is used as a control. Molecular weights are shown to the right.

B Growth curve showing parasite growth rate (parasitemia at Day X/parasitemia at Day 1) over 5 days for WT and two clones of SMC3-3HA-*glmS* parasites in the absence or presence of glucosamine (GlcN). Glucosamine treatment was started 96 h (two IDC cycles) before Day 1 to ensure SMC3 knockdown during the days sampled (Fig EV3A). Uninfected red blood cells (Blood) served as reference of background. Error bars indicate standard deviation of three technical replicates (n = 3). A two-way ANOVA with Tukey *post hoc* test was used for statistical analysis. No significant differences were found.

Source data are available online for this figure.

To gain insight into the transcriptional function of SMC3, we performed a GO enrichment analysis of genes that were up- and downregulated specifically in response to SMC3 knockdown at 12, 24, and 36 hpi. Downregulated genes were most significantly represented by the biological process categories of "protein insertion into membrane" ($q = 0.017$, Dataset EV18) at 12 hpi, "chromosome organization" ($q = 1.0 \times 10^{-3}$, Dataset EV19) and "chromosome segregation" ($q = 1.0 \times 10^{-3}$, Dataset EV19) at 24 hpi, and "mitotic cell cycle" ($q = 9 \times 10^{-8}$, Dataset EV20) and "cell cycle process" (1.7×10^{-5}, Dataset EV20) at 36 hpi.

At 12 and 24 hpi, upregulated genes were most significantly represented by the biological process categories of "movement in host environment" (12 hpi: $q = 1.8 \times 10^{-7}$, Dataset EV18; 24 hpi: $q = 1.3 \times 10^{-5}$, Dataset EV19) and "entry into host" (12 hpi: $q = 1.8 \times 10^{-7}$, Dataset EV18; 24 hpi: $q = 1.3 \times 10^{-5}$, Dataset EV19). Genes included in these categories are involved in egress and invasion of the red blood cell (reviewed in Cowman *et al*, 2012, 2017). Indeed, a substantial percentage of invasion-related genes defined in Hu *et al* (2010) were significantly upregulated upon SMC3 depletion at 12 and 24 hpi, but not 36 hpi (Dataset EV21). At 36 hpi, upregulated genes were most significantly represented by the biological process categories "translation" ($q = 1.4 \times 10^{-44}$) and ribonucleoprotein complex biogenesis (1.82×10^{-15}), subunit organization (2.47×10^{-13}), and assembly (2.95×10^{-13}; Dataset EV20). Many of the genes included in this category encode ribosomal proteins and eukaryotic translation initiation factor subunits that are highly expressed in trophozoite parasites (Painter *et al*, 2018b).

Comparison of our RNA-seq data to the time course transcriptomics data from (Data ref: Painter *et al*, 2018a; Painter *et al*, 2018b) revealed that SMC3 depletion at 12, 24, or 36 hpi caused downregulation of genes that normally reach their peak expression in the trophozoite stage (Fig 5C). Interestingly, SMC3 depletion at 12 hpi caused upregulation of genes that normally reach their peak

Figure 5. SMC3 inducible knockdown results in deregulation of genes across the IDC.

A RNA-seq of an SMC3-3HA-*glmS* clone shows *smc3* transcript levels (FPKM) at 12 (blue, $q = 8.5 \times 10^{-3}$), 24 (coral, $q = 1.3 \times 10^{-39}$), and 36 (green, $q = 4.1 \times 10^{-54}$) hpi in the absence (filled circles) or presence (empty circles) of glucosamine (GlcN). Error bars represent standard deviation of three technical replicates except for the untreated 12 hpi parasites, for which there were two replicates. P-values are calculated with a Wald test for significance of coefficients in a negative binomial generalized linear model as implemented in DESeq2 (Love *et al*, 2014). q = Bonferroni corrected P-value. Asterisks indicate q values < 0.5. Corresponding data can be found in Datasets EV9–EV11.

B MA plots of $\log_2$ (glucosamine-treated/untreated, M) plotted over the mean abundance of each gene (A) at 12, 24, and 36 hpi. Transcripts that were significantly higher (above x-axis) or lower (below x-axis) in abundance in the presence of glucosamine after filtering are highlighted in red ($q \leq 0.1$). *smc3* is highlighted in black. Dotted lines indicate a $\log_2$ fold change of 0.5. Three technical replicates were used for untreated and glucosamine-treated parasites, except for the untreated 12 hpi parasites, for which there were two replicates. P-values were calculated with a Wald test for significance of coefficients in a negative binomial generalized linear model as implemented in DESeq2 (Love *et al*, 2014). q = Bonferroni corrected P-value.

C Frequency plots showing the time in the IDC (hpi) of peak transcript level [comparison to transcriptomics time course in Data ref: Painter *et al* (2018a); Painter *et al* (2018b) for genes that are significantly downregulated (gray) or upregulated (black) following SMC3 knockdown at 12, 24, and 36 hpi.

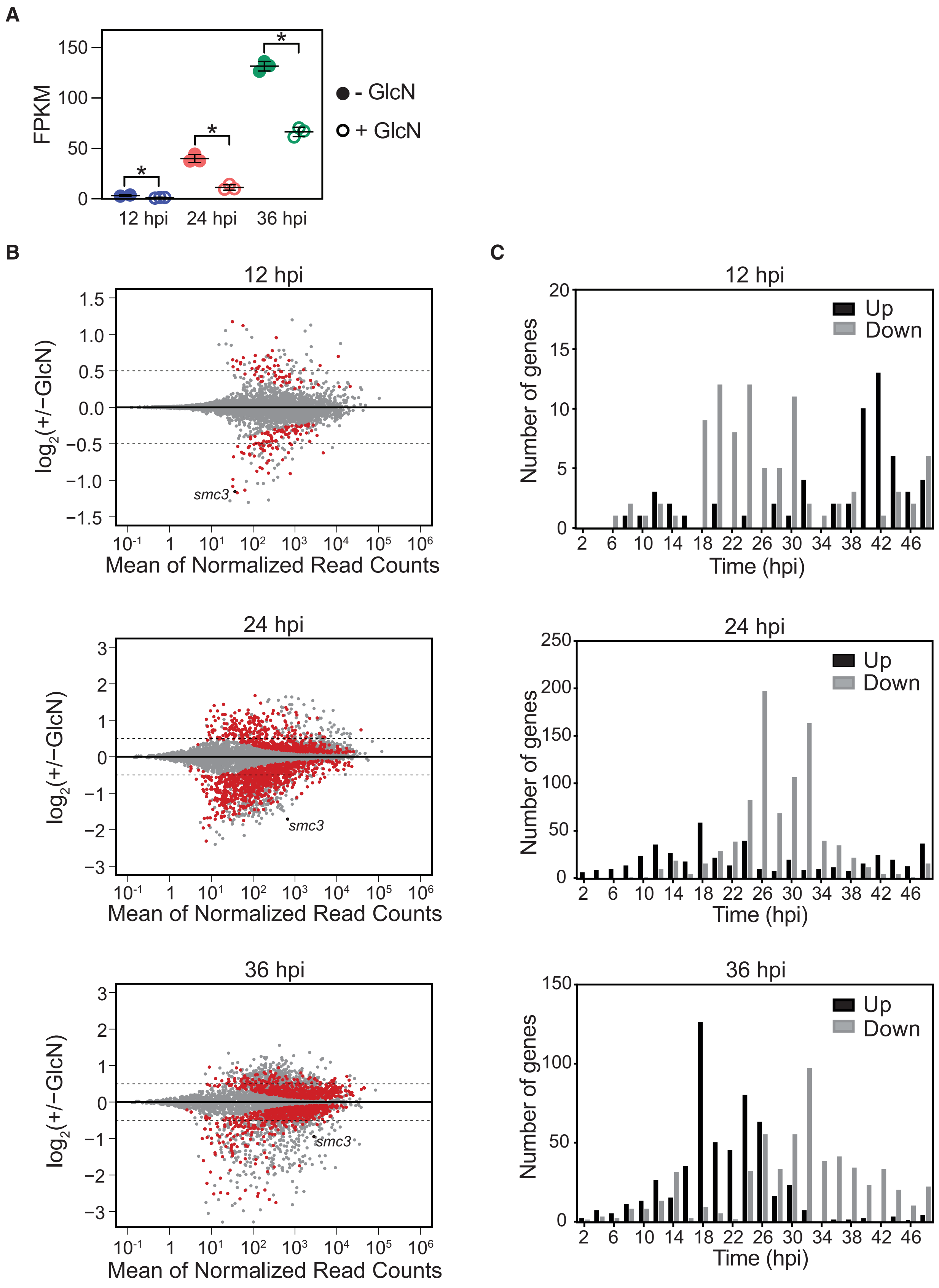

Figure 5.

 © 2023 The Authors

expression in late stages (40-48 hpi) while at 36 hpi, most upregulated genes normally reach their peak expression in late rings and early trophozoites (Fig 5C). Taken together, these data suggest a stage-specific role of SMC3.

SMC3 is involved in transcriptional regulation of genes involved in invasion and egress

To provide evidence for a direct function of SMC3 in the transcriptional regulation of these up- and downregulated genes, we compared our SMC3 ChIP-seq data to our RNA-seq data at 12 and 36 hpi. Metagene analysis from the ChIP-seq data showed that SMC3 was absent from the promoter regions of genes that were downregulated in response to its knockdown at 12 and 36 hpi (Figs 6A and EV5A). In contrast, SMC3 was enriched in the promoter regions of genes that were upregulated in response to its knockdown at 12 hpi (Fig 6A, left and EV5A), a pattern that was not seen for a control ChIP (Fig EV5B) (Data ref: Baumgarten *et al*, 2022a; Baumgarten *et al*, 2022b). Indeed, this enrichment of SMC3 at the promoters of upregulated genes was present in early, but not late-stage parasites (Figs 6B, left and EV5C), suggesting that SMC3 binding has a direct effect on the transcription of genes that are upregulated in its absence, whether naturally or via knockdown. The same trends were not observed for the 36 hpi time point. There was a slight enrichment of SMC3 in the bodies of genes that were upregulated in response to its knockdown at 36 hpi (Fig 6A, right), but this enrichment was present at all time points (Fig 6B, right). Thus, SMC3 binding does not correlate with the temporal transcription of genes that are up- or downregulated in response to its knockdown in late-stage parasites.

Because genes that are significantly upregulated upon SMC3 knockdown at 12 hpi normally reach peak expression late in the cell cycle (Fig 5C), are depleted of SMC3 at 36 hpi (Fig 6B, left), and are most significantly represented by GO terms pertaining to invasion and egress (Dataset EV18), we hypothesized that SMC3 helps to repress these genes until their appropriate time of expression late in the cell cycle. Metagene analysis showed that SMC3 is naturally enriched at the promoter regions of invasion-related genes as defined in Hu *et al* (2010) in early, but not late-stage parasites (Figs 6C and EV5D). Indeed, genes that are significantly upregulated upon SMC3 knockdown at 12 hpi show an increase in chromatin accessibility [Assay for Transposase-Accessible Chromatin using sequencing (ATAC-seq) data from (Data ref: Toenhake *et al*, 2018a; Toenhake *et al*, 2018b)] at their promoters at later stages of the IDC (Fig 6D), the opposite trend of SMC3 binding (Figs 6B, left and EV5C). Importantly, the observed inverse correlation between SMC3 enrichment and transcriptional activity was not seen for a randomly chosen set of genes that reach peak transcription in early-stage parasites and are repressed in late-stage parasites (Fig EV5E). Furthermore, SMC3 enrichment is not seen at genes that reach peak expression in gametocytes (Fig EV5F). None of the promoter regions for any of these gene sets showed enrichment in a control ChIP dataset (Fig EV5G) (Data ref: Baumgarten *et al*, 2022a; Baumgarten *et al*, 2022b). These analyses further support a potential role for SMC3 in the specific transcriptional regulation of invasion-related genes.

Examples of invasion-related genes that are upregulated upon SMC3 knockdown include the rhoptry-associated protein 2 (*rap2*, PF3D7_0501600), glideosome-associated protein 45 (*gap45*, PF3D7_1222700), merozoite surface protein 1 (*msp1*, PF3D7_0930300), and merozoite surface protein 9 (*msp9*, PF3D7_1228600; Dataset EV21). *rap2* and *gap45* show SMC3 enrichment at their promoter regions in early, but not late-stage parasites (Figs 6E and EV5H), and depletion of SMC3 resulted in upregulation at both 12 and 24 hpi (Fig 6F). The same trend can be seen at *msp1* and *msp9*, which are upregulated upon SMC3 knockdown at 12 and 24 hpi, respectively (Fig EV5I). Comparison of the SMC3 ChIP-seq data with published ATAC-seq data (Data ref: Toenhake

Figure 6. SMC3 is involved in transcriptional regulation of genes involved in invasion and egress.

A Metagene plots showing average SMC3 enrichment (*y*-axis = ChIP/Input) in clonal SMC3-3HA-*glmS* parasites at 12 (left) and 36 (right) hpi from 1.5 kb upstream of the transcription start site (TSS) to 1.5 kb downstream of the transcription end site (TES) for genes that are significantly down- (gray) or upregulated (black) upon SMC3 knockdown. One replicate (clone A) was used for the SMC3 ChIP-seq. Dashed line represents a ChIP/Input ratio of 1.

B Metagene plots showing average SMC3 enrichment (*y*-axis = ChIP/Input) in clonal SMC3-3HA-*glmS* parasites at 12 (blue), 24 (coral), and 36 hpi (green) from 1.5 kb upstream of the transcription start site (TSS) to 1.5 kb downstream of the transcription end site (TES) for genes that are significantly upregulated upon SMC3 knockdown at 12 (left) and 36 (right) hpi. One replicate (clone A) was used for the SMC3 ChIP-seq. Dashed line represents a ChIP/Input ratio of 1.

C Metagene plot showing average SMC3 enrichment (*y*-axis = ChIP/Input) in clonal SMC3-3HA-*glmS* parasites (clone A) at 12 (blue), 24 (coral), and 36 hpi (green) from 1.5 kb upstream of the transcription start site (TSS) to 1.5 kb downstream of the transcription end site (TES) for invasion-related genes, as defined in Hu *et al* (2010). Dashed line represents a ChIP/Input ratio of 1.

D Metagene plot showing average chromatin accessibility [ATAC-seq (RPM)/gDNA (RPM)] from Data ref: Toenhake *et al* (2018a); Toenhake *et al* (2018b) across the IDC from 1.5 kb upstream of the transcription start site (TSS) to 1.5 kb downstream of the transcription end site (TES) for genes that are significantly upregulated upon SMC3 knockdown at 12 hpi.

E ChIP-seq data showing enrichment of SMC3 (*y*-axis = ChIP/Input) at 12 (blue), 24 (coral), and 36 (green) hpi in clonal SMC3-3HA-*glmS* parasites at the *rhoptry-associated protein 2* (*rap2*, PF3D7_0501600) and the *glideosome-associated protein 45* (*gap45*, PF3D7_1222700) gene loci. The *x*-axis is DNA sequence, with the gene represented by a black box with white arrowheads to indicate transcription direction. One replicate (clone A) was used for ChIP-seq. ATAC-seq data from closely corresponding time points (15, 25, and 35 hpi) from Data ref: Toenhake *et al* (2018a); Toenhake *et al* (2018b) are shown in gray, with the *y*-axis representing ATAC-seq (RPM)/gDNA (RPM).

F RNA-seq of an SMC3-3HA-*glmS* clone shows transcript levels (FPKM) for *rap2* (PF3D7_0501600) at 12 ($q = 3 \times 10^{-2}$) and 24 ($q = 3.3 \times 10^{-2}$) hpi and *gap45* (PF3D7_1222700) at 12 ($q = 8 \times 10^{-1}$) and 24 ($q = 1.6 \times 10^{-2}$) hpi in the absence (black) or presence (gray) of glucosamine (GlcN). Error bars represent standard deviation of three technical replicates except for the untreated 12 hpi parasites, for which there were two replicates. *P*-values are calculated with a Wald test for significance of coefficients in a negative binomial generalized linear model as implemented in DESeq2 (Love *et al*, 2014). q = Bonferroni corrected *P*-value. Corresponding data can be found in Datasets EV9 and EV10.

G Expression values of *rap2* (PF3D7_0501600) and *gap45* (PF3D7_1222700) genes across the IDC (indicated on the *x*-axis by hpi) from the transcriptomics time course in Data ref: Painter *et al* (2018a); Painter *et al* (2018b). Data corresponding to 12 (blue), 24 (coral), and 36 (green) hpi time points are highlighted.

▶

© 2023 The Authors

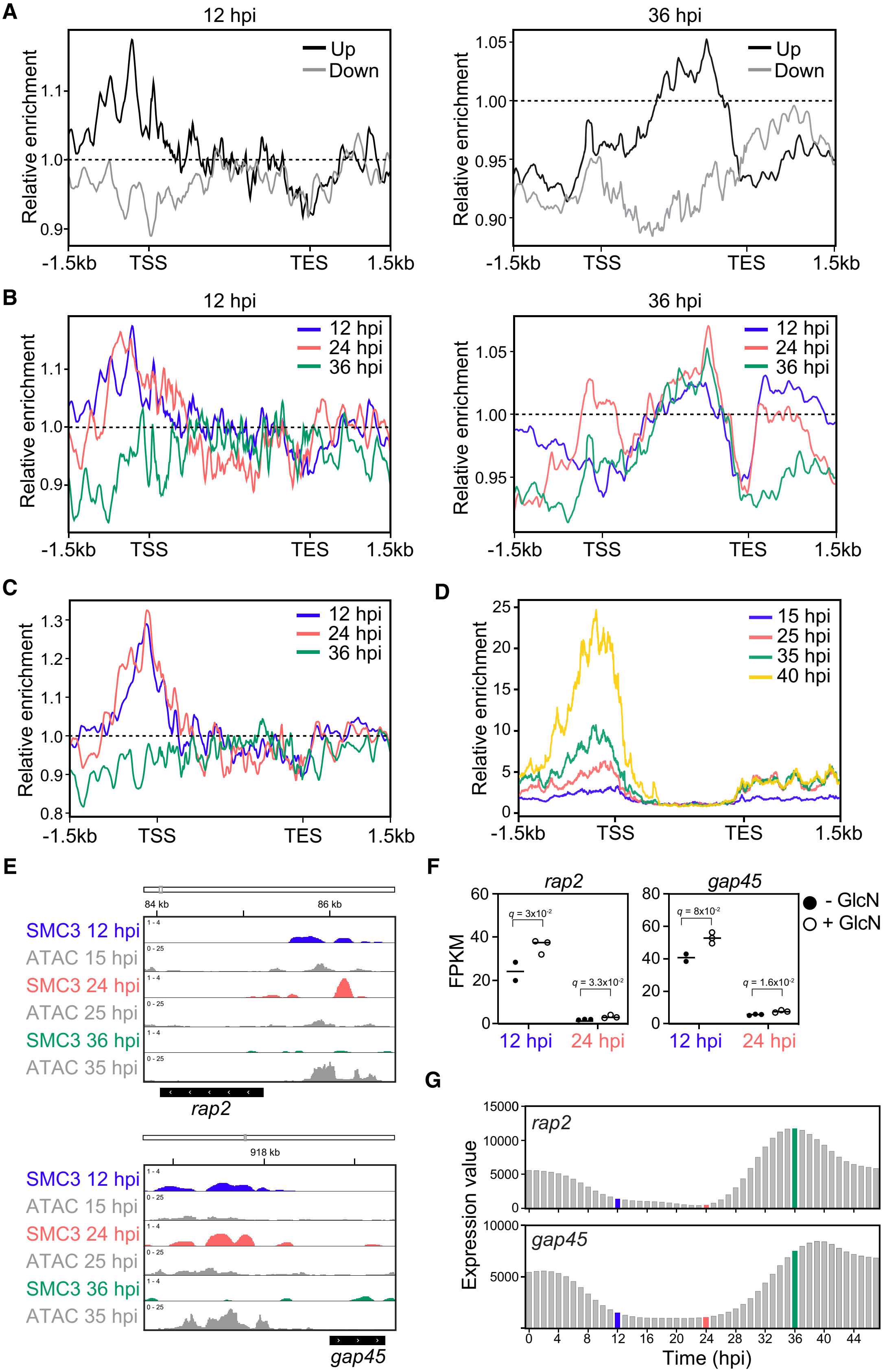

Figure 6.

© 2023 The Authors

et al, 2018a; Toenhake et al, 2018b) and mRNA dynamics data (Data ref: Painter et al, 2018a; Painter et al, 2018b) from similar time points in the IDC revealed that SMC3 binding at the promoter regions of *rap2* and *gap45* inversely correlates with chromatin accessibility (Figs 6E and EV5H) and their mRNA levels (Fig 6G), which both peak in schizont stages. These data are consistent with a role of SMC3 in repressing this gene subset until their appropriate time of expression in the IDC.

Discussion

Genome organization is key to transcriptional control and genome integrity. The human malaria parasite *P. falciparum* executes complex transcriptional programs and has a sophisticated genome organization considering that it encodes relatively few specific transcription factors and lacks key canonical genome organizing factors such as CTCF and lamins (Batsios *et al*, 2012; Heger *et al*, 2012; Ay *et al*, 2014; Bunnik *et al*, 2019). To investigate potential links between transcription and genome organization in this parasite, we have characterized SMC3, a key and conserved subunit of the multiprotein ring-shaped complex cohesin. In the organisms studied so far, cohesin plays diverse roles in genome organization such as sister chromatid cohesion during mitosis, transcription, and DNA damage repair (reviewed in Perea-Resa *et al*, 2021). Here, we used genome-wide approaches to elucidate the function of SMC3 in transcription during the IDC of *P. falciparum*.

Immunoprecipitation followed by LC–MS/MS (Dataset EV1) and co-immunoprecipitation studies (Fig 1D and E) confirmed the interaction of SMC3 with the other members of the core cohesin ring complex: SMC1 and Rad21. In addition, our studies detected interaction with a STAG domain-containing protein, which associates with the core cohesin complex and is involved in cohesin loading onto chromatin in other eukaryotic systems (Hu *et al*, 2011; Murayama & Uhlmann, 2014). Because the *P. falciparum* genome does not encode components of the canonical cohesin loading or unloading complexes, future studies with STAG may elucidate the mechanisms whereby cohesin is loaded onto chromatin in this parasite.

ChIP-seq over the course of the IDC revealed that SMC3 is most enriched in centromeric and pericentromeric regions (Figs 3A–C and EV2B and C). In other eukaryotes, cohesin is also mostly enriched around the centromeres relative to the chromosome arms (Tanaka *et al*, 1999; Tomonaga *et al*, 2000; Holzmann *et al*, 2019). Our observation that SMC3 depletion (to the extent we were able to achieve with the *glmS* system) does not inhibit parasite growth agrees with reports in *S. cerevisiae* and *D. melanogaster* in which normal growth and sister chromatid cohesion were achieved despite an 87 and 80% decrease, respectively, in Rad21, a core component of the cohesin complex (Heidinger-Pauli *et al*, 2010; Carvalhal *et al*, 2018). These studies and ours suggest that only a small fraction of cohesin is needed to successfully complete mitosis.

Centromeric clustering in interphase nuclei has been observed in several eukaryotes including *S. cerevisiae*, *D. melanogaster*, and *H. sapiens* (reviewed in Muller *et al*, 2019). The functional importance of this spatial arrangement remains poorly understood; however, it has been shown that centromeric clustering is a relevant topological constraint that can affect transcription by preventing intrachromosomal arm interactions (Tolhuis *et al*, 2011). Studies in *P. falciparum* have demonstrated centromere clustering before and during schizogony, suggesting that this organization is needed during interphase and mitosis (Hoeijmakers *et al*, 2012; Ay *et al*, 2014). One architectural factor, *Pf*HMGB1, was recently shown to play a direct role in centromere organization in the nucleus (Lu *et al*, 2021). Although *Pf*HMGB1 binds predominantly to centromeres, its depletion led to the de-regulation of many different genes to which it was not bound, suggesting that global genome organization is important for transcriptional control at the local chromatin level (Lu *et al*, 2021). *Pf*HMGB1 knockout did not lead to blood stage parasite growth inhibition, indicating that other proteins, such as cohesin or *Pf*CenH3, play a role in centromere organization and mitosis.

In addition to its potential centromeric role, we discovered that SMC3 plays a direct, extra-centromeric role in the transcriptional control of specific genes during interphase. SMC3 bound dynamically at extra-centromeric genomic locations over the course of the IDC (Figs 3 and EV2, Dataset EV4). We observed stage-specific SMC3 binding across the genome, including at the promoters of genes that were then upregulated upon SMC3 depletion in interphase parasites (Figs 3B and D, and 6, Datasets EV7, EV15, and EV16). In contrast, genes that were downregulated upon SMC3 depletion in early and late-stage parasites were not enriched for SMC3 at their promoter regions, suggesting an indirect effect (Fig 6A). And while SMC3 peak-associated genes were significantly represented by GO terms related to antigenic variation at 24 and 36 hpi, significantly up- or downregulated genes at 24 and 36 hpi did not show significant *q*-values for these or related GO terms.

Genes that were upregulated upon SMC3 depletion during interphase are generally most highly expressed in late-stage parasites (Figs 5C and 6G), a time when we observed natural depletion of SMC3 at their promoters (Figs 6B,C, and E and EV5C,H, and I). Moreover, genes that were upregulated upon SMC3 knockdown at 36 hpi, which were significantly represented by GO enrichment terms pertaining to translation and ribosome biogenesis, showed no obvious pattern of SMC3 enrichment over the IDC (Fig 6B). These data suggest that SMC3 is specifically recruited to and evicted from specific subsets of genes to facilitate their repression and transcription, respectively, over the course of the IDC.

Genes that were significantly upregulated upon SMC3 depletion during interphase were enriched for GO terms related to egress from and invasion of the RBC (Datasets EV18 and EV19). Indeed, out of a list of 63 invasion-related genes (Hu *et al*, 2010), 50% were among the genes that were upregulated upon SMC3 depletion during interphase, but not in late-stage parasites (Dataset EV21). Thus, the repressive function of SMC3 at these genes seems to be specific to interphase. The observation that growth rate was not affected by SMC3 knockdown (Fig 4B) indicates that deregulation of SMC3-bound genes did not affect normal cell cycle progression. It is possible that SMC3 is simply one factor that helps to repress invasion-related genes in interphase parasites. Future studies will be needed to investigate the contribution of other cohesin subunits such as SMC1, RAD21, and STAG in this transcriptional role.

The mechanism by which SMC3 could repress specific genes in a stage-specific manner is unclear. In the context of interaction with CTCF, cohesin has been shown to impact transcription in opposite ways, by either preventing enhancer-promoter interactions (Wendt *et al*, 2008; Nativio *et al*, 2009) or by mediating specific enhancer-

© 2023 The Authors

promoter loops (Kubo *et al*, 2021; Oh *et al*, 2021). In *P. falciparum*, the current genome-wide chromosome conformation capture (Hi-C) datasets do not provide evidence of typical enhancer-promoter interactions found in other eukaryotes (Ay *et al*, 2014; Bunnik *et al*, 2019). Moreover, in *S. cerevisiae*, a cohesin mutant resulted in the de-repression of genes located in subtelomeric regions, perhaps via disruption of local chromatin structure (Kothiwal & Laloraya, 2019). However, the invasion-related genes affected by *Pf*SMC3 are scattered across the genome (Dataset EV21). One possibility is that cohesin binding to the promoter of a gene merely inhibits the transcriptional machinery from assembling. Considering the ability of cohesin to entrap multiple DNA molecules, another intriguing possibility is that it tethers invasion-related genes together in a cluster that renders their promoters inaccessible to specific activating factors until the appropriate time of transcription. Future high-resolution chromosome conformation capture studies will reveal a potential link between spatial association and transcriptional regulation of these SMC3-controlled genes.

It is also unclear how *Pf*SMC3 achieves binding specificity and how it is evicted from binding sites at specific times in the IDC. In other organisms studied, cohesin appears to need a DNA-binding factor to achieve sequence-specific binding (Kagey *et al*, 2010; Sasca *et al*, 2019). A search for a specific binding motif within the promoter sequences of invasion-related genes bound by *Pf*SMC3 yielded no results, indicating that *Pf*SMC3 may associate with multiple factors to achieve specific binding. In *P. falciparum*, the AP2-I transcription factor (PF3D7_1007700) is involved in transcription of invasion-related genes via binding to the GTGCA motif, likely by interaction with the bromodomain protein 1 (*Pf*BDP1, PF3D7_1033700; Santos *et al*, 2017). This complex could evict SMC3 from gene promoters or simply bind in its absence. Importantly, neither *ap2-I* nor *bdp1* are bound by SMC3 or are upregulated upon SMC3 depletion, suggesting that the observed upregulation of invasion-related genes upon SMC3 depletion in early-stage parasites is a direct effect. In addition, SMC3 depletion resulted in the upregulation of AP2-I-independent invasion-related genes such as RONs, EBLs and Rhs, which have an ACAACT motif in their promoter regions (Young *et al*, 2008) and may be activated by an as-yet unidentified transcription factor. Future studies will reveal the molecular machinery that regulates the stage-specific binding of cohesin.

The present study offers insight into the role of cohesin in the temporal regulation of genes in *P. falciparum*. While the role of H3K9me3/HP1 has been well established in the transcriptional repression of clonally variant gene families and *ap2-g*, this study identifies a new factor – SMC3 – involved in the repression of HP1-independent, stage-specific genes. Given the architectural nature of cohesin, this research provides a potential link between genome organization and transcriptional control in *P. falciparum*.

Materials and Methods

Parasite culture

Blood stage 3D7 *P. falciparum* parasites were cultured as previously described in Lopez-Rubio *et al* (2009). Briefly, parasites were cultured in human RBCs supplemented with 10% v/v Albumax I (Thermo Fisher 11020), hypoxanthine (0.1 mM final concentration, C.C.Pro Z-41-M) and 10 mg gentamicin (Sigma G1397) at 4% hematocrit and under 5% O2, 5% CO_2 at 37°C. Parasites were synchronized by sorbitol (5%, Sigma S6021) lysis during ring stage followed by a plasmagel (Plasmion, Fresenius Kabi) enrichment for late blood stages 24 h later. Another sorbitol treatment 6 h afterward places the 0 h time point 3 h after the plasmagel enrichment. Thus, the window of synchronicity for cultures is ± 3 h. Parasite development was monitored by Giemsa staining. Parasites were harvested at 1–5% parasitemia.

Generation of strains

The SMC3-3HA-*glms* strain was generated using a two-plasmid system (pUF1 and pL7) based on the CRISPR/Cas9 system previously described in Ghorbal *et al* (2014). A 3D7 wild-type bulk ring stage culture was transfected with 25 μg pUF1-Cas9 and 25 μg of pL7-*Pf*SMC3-3HA-*glmS* containing the single guide RNA (sgRNA) -encoding sequence 5′-CCTAGAAAATTAGAACAATT-3′ targeting the 3′ UTR of PF3D7_0414000. The pL7-*Pf*SMC3-3HA-*glmS* plasmid also contained the homology repair construct 5′-AGATAGAGAGAGTTAT ATATCTAAAGGAACAAAGAATGAGGCCTACGAAATTATTAGCATT GTATAAAAAAAAAAAGAAAAAAAAAAGAAAAAAAAAAAAAGATT ATATATATAATATATGTTGACAATTAATAAATATATTTGTATAT ATCTGTTAACTAATTATGAAAATTTTTGAATCAATAAATTTTTT AAATAACAAAAAAAAAAAAAATATATATATTATATATATATTTT ATATTTTATATTTTCTTGTAATTTTTGTTTTTTTAGGAGGAAAAAC ATGCCCTAGAAAATggcggtggaTACCCTTACGATGTGCCTGATTACG CGTAtCCcTAtGAcGTaCCaGAcTAtGCGTACCCtTAtGAcGTtCCgG ATTAtGCtcacggggtgTAAGCGGCCGCGGTCTTGTTCTTATTTTCTC AATAGGAAAAGAAGACGGGATTATTGCTTTACCTATAATTATAGC GCCCGAACTAAGCGCCCGGAAAAAGGCTTAGTTGACGAGGATGGA GGTTATCGAATTTTCGGCGGATGCCTCCCGGCTGAGTGTGCAGAT CACAGCCGTAAGGATTTCTTCAAACCAAGGGGGTGACTCCTTGAA CAAAGAGAAATCACATGATCTTCCAAAAAACATGTAGGAGGGGA CAACAATTTGGTTTTGTTTTTTTCTTTAGGTTTTGAGAAAAACAAA TAGGAAATACAAAAAAAAAAAAAAAAAAAAAAAAAAAAAAAAAA AAAAATGTATTTTTACATATGCACTTGGATTATTTTATTTTTATTA TTTTTCTTTATATAAAGTAAAAATATACATAAGTATGCTTATTTA TTACATAAGAGTTTATTTAAGAAAGGTTTCTTTTTCATAATATTG TGTGCATGAGTTTTTTTTTATTTTATTTTTTTTTTTTTATTTCTGTAA CGAAAAGGATATTAAAAAAAAATAATAAAA-3′ (synthesized by GenScript Biotech [Piscataway, NJ, USA]). This homology repair construct comprises a 3 × Hemaglutinin (3HA) – encoding sequence followed by a *glmS* ribozyme-encoding sequence (Prommana *et al*, 2013), which are flanked by 300 bp homology repair regions upstream and downstream of the Cas9 cut site, excluding the gene STOP codon. The sgRNA sequence was designed using Protospacer (MacPherson & Scherf, 2015). The sgRNA sequence uniquely targeted a single sequence in the genome. As the sgRNA sequence encompasses the STOP codon, its modification via the addition of the 3HA and *glmS*-encoding sequences renders the modified parasites refractory to further Cas9 cleavage at this locus. After transfection, drug selection was applied for 5 days at 2.67 nM WR99210 (Jacobus Pharmaceuticals) and 1.5 μM DSM1 (MR4/BEI Resources). Parasites reappeared approximately 3 weeks after transfection, and 5-fluorocytosine was used to negatively select the pL7 plasmid.

The SMC1-3HA-dd and STAG-3HA-dd strains were generated using the method of selection-linked integration (SLI) previously described in Birnbaum *et al* (2017). Homology regions

 © 2023 The Authors

corresponding to the 894 and 639 bp at the 3′ ends of *smc1* (PF3D7_1130700) and *stag* (PF3D7_1456500), respectively, were amplified using either SMC1-HR-FW/RV or STAG-HR-FW/RV (Dataset EV22). The obtained PCR fragments were fused to sequences encoding a 3HA epitope tag and a ligand-regulatable FKBP protein destabilization domain (Armstrong & Goldberg, 2007), followed by a skip peptide and the ubiquinone-independent dihydroorotate dehydrogenase (yDHODH) resistance marker. A 3D7 wild-type bulk ring stage culture was transfected with 50 μg of either pSLI-SMC1-3HA-dd or pSLI-STAG-3HA-dd and selected first with 2.67 nM WR99210 (Jacobus Pharmaceuticals), cultured in the constant presence of Shield-1 (Aoubious INC) at 500 nM. Parasites reappeared approximately 5 weeks after transfection and positive selection for integration was performed via the addition 0.75 μM DSM1 (MR4/BEI Resources).

All cloning was performed using KAPA HiFi DNA Polymerase (Roche 07958846001), In-Fusion HD Cloning Kit (Clontech 639649), and XL10-Gold Ultracompetent *E. coli* (Agilent Technologies 200315). All the parasite lines were cloned by limiting dilution, and integration at the targeted genomic locus was confirmed by PCR (Dataset EV22, Fig EV1A–C) and Sanger sequencing.

SMC3 immunoprecipitation and mass spectrometry

An SMC3-3HA-*glms* clone (n = 3 technical replicates) and WT culture (n = 3 technical replicates), as a negative control, were synchronized. Late stage parasites (1.5×10^9 parasites) were enriched using Percoll density gradient separation and then cross-linked with 1 ml 0.5 mM dithiobissuccinimidyl propionate (DSP; Thermo Fisher 22585) in DPBS for 60 min at 37°C (as in Mesén-Ramírez *et al*, 2016). Cross-linked parasites were centrifuged at 4,000 *g* for 5 min at 4°C, and the pellet was washed twice with DPBS at 4°C. The pellet was lysed with 10 volumes of RIPA buffer (10 mM Tris–HCl pH 7.5, 150 mM NaCl, 0.1% SDS, 1% Triton) containing protease and phosphatase inhibitor cocktail (Thermo Fisher 78440) and 1 U/μl of Benzonase (Merck 71206). The lysates were cleared by centrifugation at 16,000 *g* for 10 min at 4°C. Supernatants were incubated with 25 μl of anti-HA Dynabeads (Thermo Fisher 88836) overnight with rotation at 4°C. Beads were collected with a magnet and washed five times with 1 ml RIPA buffer, then five times with 1 ml DPBS, and then once with 1 ml 25 mM NH_4HCO_3 (Sigma 09830). The beads were reduced with 100 mM dithiothreitol (Sigma D9779), alkylated with 55 mM iodoacetamide (Sigma I1149), and subjected to on-bead digestion using 1 μg of trypsin (Thermo Fisher 90059). The resulting peptides were desalted using C18 ziptips (Merck ZTC04S096) and sent for MS analysis.

Peptides were separated by reverse phase HPLC (Thermo Fisher Easy-nLC1000) using an EASY-Spray column, 50 cm × 75 μm ID, PepMap RSLC C18, 2 μm (Thermo Fisher ES803A) over a 70-min gradient before nanoelectrospray using a Q Exactive HF-X mass spectrometer (Thermo Fisher). The mass spectrometer was operated in a data-dependent mode. The parameters for the full scan MS were as follows: resolution of 60,000 across 350–1,500 m/z, AGC $1e^5$ (as in Kensche *et al*, 2016), and maximum injection time (IT) 150 ms. The full MS scan was followed by MS/MS for the top 15 precursor ions in each cycle with an NCE of 30 and dynamic exclusion of 30 s and maximum IT of 96 ms. Raw mass spectral data files (.raw) were searched using Proteome Discoverer 2.3.0.523 (Thermo Fisher) with the SEQUEST search engine. The search parameters were as follows: 10 ppm mass tolerance for precursor ions; 0.8 Da fragment ion mass tolerance; two missed cleavages of trypsin; fixed modification was carbamidomethylation of cysteine; and variable modifications were methionine oxidation, CAMthiopropanoyl on lysine or protein N-terminal, and serine, threonine, and tyrosine phosphorylation. Only peptide spectral matches (PSMs) with an XCorr score greater than or equal to 2 and an isolation interference less than or equal to 30 were included in the data analysis.

Protein fractionation and western blot analysis

Parasites were washed once with Dulbecco's phosphate-buffered saline (DPBS, Thermo Fisher 14190), then resuspended in cytoplasmic lysis buffer (25 mM Tris–HCl pH 7.5, 10 mM NaCl, 1.5 mM $MgCl_2$, 1% IGEPAL CA-630, and 1× protease inhibitor cocktail ["PI", Roche 4693132001]) at 4°C and incubated on ice for 30 min. The cytoplasmic lysate was cleared with centrifugation (13,500 *g*, 10 min, 4°C). The pellet (containing the nuclei) was resuspended in 3.3 times less volume of nuclear extraction buffer (25 mM Tris–HCl pH 7.5, 600 mM NaCl, 1.5 mM $MgCl_2$, 1% IGEPAL CA-630, PI) than cytoplasmic lysis buffer at 4°C, transferred to 1.5 ml sonication tubes (Diagenode C30010016, 300 μl per tube), and sonicated for 5 min total (10 cycles of 30 s on/off) in a Diagenode Pico Bioruptor at 4°C. This nuclear lysate was cleared with centrifugation (13,500 *g*, 10 min, 4°C). Protein samples were supplemented with NuPage Sample Buffer (Thermo Fisher NP0008) and NuPage Reducing Agent (Thermo Fisher NP0004) and denatured for 10 min at 70°C. Proteins were separated on a 4–12% Bis-Tris NuPage gel (Thermo Fisher NP0321) and transferred to a PVDF membrane with a Trans-Blot Turbo Transfer system (Bio-Rad). The membrane was blocked for 1 h with 1% milk in PBST (PBS, 0.1% Tween 20) at 25°C. HA-tagged proteins and histone H3 were detected with anti-HA (Abcam ab9110, 1:1,000 in 1% milk-PBST) and anti-H3 (Abcam ab1791, 1:2,500 in 1% milk-PBST) primary antibodies, respectively, followed by donkey anti-rabbit secondary antibody conjugated to horseradish peroxidase ("HRP", Sigma GENA934, 1:5,000 in 1% milk-PBST). SMC3 was detected with an in-house-generated anti-SMC3 antibody (see below, 1:500 in 1% milk-PBST), followed by sheep HRP anti-mouse secondary antibody (Cytiva NA931, 1:5,000 in 1% milk-PBST). HRP signal was developed with SuperSignal West Pico Plus or Femto chemiluminescent substrate (Thermo Fisher 34580 or 34096, respectively) and imaged with a ChemiDoc XRS+ (Bio-Rad).

SMC3 antibody generation

The coding sequence for the N-terminal region (amino acids 1-253) of *Pf*SMC3 (PF3D7_0414000) was amplified from cDNA of 3D7 wild-type parasites and inserted into the pGEX expression vector. The plasmid was verified by sequencing and used to transform SHuffle T7 expression bacteria (New England BioLabs C3026J). Recombinant protein expression was induced with 100 μM IPTG overnight with shaking at 16°C, and the pellet was frozen at −80°C until use. Soluble GST-tagged recombinant protein was purified using Glutathione Sepharose 4 Fast Flow (Cytiva GE17-5132-03), according to manufacturer's instructions. Briefly, the bacterial pellet was resuspended in cold lysis buffer (50 mM Tris–HCl pH 7.4, 50 mM NaCl, 5 mM EDTA with protease inhibitor cocktail ["PI", Roche 4693132001]). The lysate was sonicated on ice with a probe sonicator (VibraCell, USA; 10 cycles of 10 s on and

© 2023 The Authors

1 min off). The sample was centrifuged at 18,000 *g* for 1 h at 4°C, and the soluble lysate was incubated with Glutathione Sepharose beads (Cytiva GE17-5132-03) overnight with gentle tumbling at 4°C. The next day, the lysate with beads was loaded onto chromatography columns (Poly-Prep, BioRad), and the beads were washed with cold PBS containing 5 mM EDTA. Recombinant protein was eluted with elution buffer (50 mM Tris–HCl pH 8, 10 mM reduced glutathione), buffer exchanged into PBS (Vivaspin, GE Healthcare), and stored at −20°C until use. Final protein concentration was obtained using Protein Assay (BioRad).

Purified recombinant protein was used to immunize C57BL/6 mice. Ten micrograms were used for the first immunization in Freund's Complete Adjuvant (Sigma F5881) and three subsequent boosters in Freund's Incomplete Adjuvant (Sigma F5506). Animals were housed in the Institute Pasteur animal facilities, accredited by the French Ministry of Agriculture for performing experiments on live rodents. Work on animals was performed in compliance with French and European regulations on care and protection of laboratory animals (EC Directive 2010/63, French Law 2013-118, February 6th, 2013). All experiments were approved by the Ethics Committee CETEA Institute Pasteur and registered under the reference Permit Number dap180040, issued in 2018. The *Pf*SMC3 antibody was purified from pooled mouse serum using rProtein G Agarose (Invitrogen 15920) according to manufacturer's instructions. The final purified antibody was dialyzed in PBS using a Float-A-Lyzer (Repligen). Purified antibodies were stored at −20°C in 50% Glycerol.

Co-immunoprecipitation and western blot analysis

SMC1-3HA-dd and STAG-3HA-dd parasites were synchronized and harvested at 36 hpi (1.1×10^8 parasites). Parasite cultures were centrifuged at 800 *g* for 3 min at 25°C. Medium was removed and the RBCs were lysed with 10 ml 0.075% saponin (Sigma S7900) in DPBS at 37°C. The parasites were centrifuged at 3,250 *g* for 3 min at 25°C and washed twice with 10 ml DPBS at 4°C. The supernatant was removed, and the parasite pellet was resuspended in 1 ml 0.5 mM dithiobissuccinimidyl propionate (DSP; Thermo Fisher 22585) in DPBS for 30 min at 25°C (as in Mesén-Ramírez *et al*, 2016). The reaction was quenched with 6 ml 25 mM Tris–HCl pH 7.5 for 10 min at 25°C. Cross-linked parasites were centrifuged at 4,000 *g* for 5 min at 4°C, and the pellet was washed with DPBS at 4°C. The supernatant was removed, and the cross-linked parasite pellets were snap-frozen and stored at -80°C.

For each IP, 25 µl of Protein G Dynabeads (Invitrogen 10004D) were washed twice with ChIP dilution buffer (16.7 mM Tris–HCl pH 8, 150 mM NaCl, 1.2 mM EDTA pH 8, 1% Triton X-100, 0.01% SDS) using a DynaMag magnet (Thermo Fisher 12321D). The beads were resuspended in 200 µl 0.02% Tween-20 in DPBS with 1 µg of anti-HA antibody (Abcam ab9110) or IgG (Diagenode C15410206) and incubated with rotation at 4°C for 6 h.

All further steps were performed at 4°C. The cross-linked parasites were resuspended in 900 µl of cytoplasmic lysis buffer (10 mM Tris–HCl pH 7.5, 10 mM NaCl, 1 mM EDTA, 0.65% Nonidet-P40, PI) and rotated for 30 min. The lysates were centrifuged for 5 min at 2,000 *g* and the supernatant was removed. The pellet was resuspended in 1 ml cytoplasmic wash buffer (10 mM Tris–HCl pH 7.5, 150 mM NaCl, 1 mM EDTA, PI) and rotated for 10 min. The lysates were centrifuged for 5 min at 5,000 *g*, and the supernatant was removed. The pellet was resuspended in 200 µl nuclear lysis buffer (10 mM Tris–HCl pH 7.5, 500 mM NaCl, 1 mM EDTA pH 8, 1% sodium deoxycholate, 1% IGEPAL, 0.1% SDS, PI), transferred to 1.5 ml sonication tubes (Diagenode C30010016), and sonicated for 2.5 min total (5 cycles of 30 s on/off) in a Diagenode Pico Bioruptor. The sonicated extracts were centrifuged at 13,500 *g* for 10 min. The supernatant was diluted with 400 µl dilution buffer 2 (10 mM Tris–HCl pH 7.5, 0.5 mM EDTA).

Using a DynaMag magnet, the antibody-conjugated Dynabeads were washed once with 200 µl of dilution buffer 1 (10 mM Tris pH 7.5, 0.5 mM EDTA, 150 mM NaCl) and resuspended in 200 µl dilution buffer 2 (10 mM Tris–HCl pH 7.5, 0.5 mM EDTA). The washed antibody-conjugated Dynabeads were added to the diluted nuclear lysate and incubated overnight with rotation at 4°C. The beads were collected on a DynaMag magnet and the supernatant was removed and kept as flow through. The beads were washed three times with 200 µl of wash buffer (10 mM Tris–HCl pH 7.5, 150 mM NaCl, 0.5 mM EDTA, 0.05% IGEPAL). The beads were resuspended in 30 µl of 2× NuPage Sample Buffer (Thermo Fisher NP0008) and 3.4 µl of NuPage Reducing Agent (Thermo Fisher NP0004) and denatured for 5 min at 95°C. The eluate was separated from the beads with the DynaMag magnet. Lysates and the bead eluate were separated on a 3-8% Tris-Acetate NuPage gel (Thermo Fisher EA0375) and transferred to a PVDF membrane with a Trans-Blot Turbo Transfer system (Bio-Rad). Membranes were blocked with 1% milk-PBST (PBS, 0.1% Tween 20). HA-tagged SMC1 and STAG proteins were detected with anti-HA conjugated to HRP (Cell Signaling 14031, C29F4, 1:5,000 in 1% milk-PBST). SMC3 was detected with anti-*Pf*SMC3 antibody (1:500 in 1% milk-PBST) followed by sheep HRP anti-mouse secondary antibody (Cytiva NA931, 1:5,000 in 1% milk-PBST). Histone H3 was detected with Anti-H3 (Abcam ab1791, 1:1000 in 1% milk-PBST), followed by donkey HRP anti-rabbit secondary antibody (Sigma GENA934, 1:5,000 in 1% milk-PBST). HRP signal was developed with SuperSignal West Pico Plus or West Femto chemiluminescent substrate (Thermo Fisher 34580 or 34096, respectively) and imaged with a ChemiDoc XRS+ (Bio-Rad).

Immunofluorescence assays and image acquisition

iRBCs were washed once with DPBS (Thermo Fisher 14190) at 37°C and fixed in suspension in 4% paraformaldehyde (EMS 15714) with 0.0075% glutaraldehyde (EMS 16220) in PBS for 20 min at 25°C, as described previously (Tonkin *et al*, 2004). The subsequent steps were performed at 25°C as described in Mehnert *et al* (2019), with minor changes. After washing once with PBS, cells were permeabilized with 0.1% Triton-X 100 for 10 min followed by three PBS washes. Free aldehyde groups were quenched with 50 mM NH_4Cl for 10 min, followed by two washes with PBS. Cells were blocked with 3% bovine serum albumin (BSA; Sigma A4503-50G) in PBS for 30 min. Cells were incubated with anti-HA (Abcam ab9110, 1:1,000 in 3% BSA in PBS) and/or anti-HP1 [from (Chen *et al*, 2016; Genscript, 1:1,000 in 3% BSA in PBS)] primary antibody for 1 h followed by three 10 min washes with 0.5% Tween® 20/PBS. Cells were incubated with anti-rabbit Alexa Fluor 488- or 633-conjugated secondary antibodies (Invitrogen A-11008 or A-21070, 1:2,000 in 3% BSA in PBS) with DAPI (FluoProbes FP-CJF800, 1 µg/ml) for 45 min followed by three 10 min washes with 0.5% Tween®20/PBS. Cells were washed once more with PBS and placed onto polyethyleneimine-coated slides (Thermo Scientific 30-42H-RED-

 © 2023 The Authors

CE24). Once adhered to the slide, cells were washed twice with PBS and mounted with VectaShield® (Vector Laboratories). Images were acquired using a Deltavision Elite imaging system (GE Healthcare), and Fiji (http://fiji.sc) was used for analysis using the least manipulation possible.

SMC3 chromatin immunoprecipitation sequencing and data analysis

Two clonal populations (clone A and B) of SMC3-3HA-*glmS* parasites were tightly synchronized and harvested at 12 (10^{10} of clone A and B parasites), 24 (4.3×10^8 and 6.8×10^8 of clone A and B parasites, respectively) and 36 hpi (3.6×10^8 and 3×10^8 of clone A and B parasites, respectively). Parasite culture was centrifuged at 800 *g* for 3 min at 25°C. Medium was removed and the RBCs were lysed with 10 ml 0.075% saponin (Sigma S7900) in DPBS at 37°C. The parasites were centrifuged at 3,250 *g* for 3 min at 25°C and washed with 10 ml DPBS at 37°C. The supernatant was removed, and the parasite pellet was resuspended in 10 ml of PBS at 25°C. The parasites were cross-linked by adding methanol-free formaldehyde (Thermo Fisher 28908; final concentration 1%) and incubating with gentle agitation for 10 min at 25°C. The cross-linking reaction was quenched by adding glycine (final concentration 125 mM, Sigma G8899) and incubating with gentle agitation for 5 min at 25°C. Parasites were centrifuged at 3,250 *g* for 5 min at 4°C and the supernatant removed. The pellet was washed with DPBS and centrifuged at 3,250 *g* for 5 min at 4°C. The supernatant was removed, and the cross-linked parasite pellets were snap-frozen.

For each time-point, 200 µl of Protein G Dynabeads (Invitrogen 10004D) were washed twice with 1 ml ChIP dilution buffer (16.7 mM Tris–HCl pH 8, 150 mM NaCl, 1.2 mM EDTA pH 8, 1% Triton X-100, 0.01% SDS) using a DynaMag magnet (Thermo Fisher 12321D). The beads were resuspended in 1 ml ChIP dilution buffer with 8 µg of anti-HA antibody (Abcam ab9110) and incubated on a rotator at 4°C for 6 h.

The cross-linked parasites were resuspended in 4 ml of lysis buffer (10 mM HEPES pH 8, 10 mM KCl, 0.1 mM EDTA pH 8, PI) at 4°C, and 10% Nonidet-P40 was added (final concentration 0.25%). The parasites were lysed in a prechilled dounce homogenizer (200 strokes for 12 hpi parasites and 100 strokes for 24 and 36 hpi parasites). The lysates were centrifuged for 10 min at 13,500 *g* at 4°C, the supernatant was removed, and the pellet was resuspended in SDS lysis buffer (50 mM Tris–HCl pH 8, 10 mM EDTA pH 8, 1% SDS, PI) at 4°C (3.6 ml for the 12 hpi sample and 1.8 ml for the 24 ad 36 hpi samples). The liquid was distributed into 1.5 ml sonication tubes (Diagenode C30010016, 300 µl per tube) and sonicated for 12 min total (24 cycles of 30 s on/off) in a Diagenode Pico Bioruptor at 4°C. The sonicated extracts were centrifuged at 13,500 *g* for 10 min at 4°C and the supernatant, corresponding to the chromatin fraction, was kept. The DNA concentration for each time point was determined using the Qubit dsDNA High Sensitivity Assay Kit (Thermo Fisher Scientific Q32851) with a Qubit 3.0 Fluorometer (Thermo Fisher Scientific). For each time point, chromatin lysate corresponding to 100 ng of DNA was diluted in SDS lysis buffer (final volume 200 µl) and kept as "input" at −20°C. For clones A and B, chromatin lysate corresponding to 19 and 18 µg (12 hpi), 2 and 1.4 µg (24 hpi) and 3 and 5.2 µg (36 hpi) of DNA, respectively, was diluted 1:10 in ChIP dilution buffer at 4°C.

Using a DynaMag magnet, the antibody-conjugated Dynabeads were washed twice with 1 ml ChIP dilution buffer and resuspend in 100 µl of ChIP dilution buffer at 4°C. Then the washed antibody-conjugated Dynabeads were added to the diluted chromatin sample and incubated overnight with rotation at 4°C. The beads were collected on a DynaMag into eight different tubes per sample, the supernatant was removed, and the beads in each tube were washed for 5 min with gentle rotation with 1 ml of the following buffers, sequentially:

- Low salt wash buffer (20 mM Tris–HCl pH 8, 150 mM NaCl, 2 mM EDTA pH 8, 1% Triton X-100, 0.1% SDS) at 4°C.
- High salt wash buffer (20 mM Tris–HCl pH 8, 500 mM NaCl, 2 mM EDTA pH 8, 1% Triton X-100, 0.1% SDS) at 4°C.
- LiCl wash buffer (10 mM Tris–HCl pH 8, 250 mM LiCl, 1 mM EDTA pH 8, 0.5% IGEPAL CA-630, 0.5% sodium deoxycholate) at 4°C.
- TE wash buffer (10 mM Tris–HCl pH 8, 1 mM EDTA pH 8) at 25°C.

After the washes, the beads were collected on a DynaMag, the supernatant was removed, and the beads for each time point were resuspended in 800 µl of elution buffer and incubated at 65°C for 30 min with agitation (1,000 rpm 30 s on/off). The beads were collected on a DynaMag and the eluate, corresponding to the "ChIP" samples, was transferred to a different tube.

For purification of the DNA, both "ChIP" and "Input" samples were incubated for approximately 10 h at 65°C to reverse the cross-linking. Two hundred microliters of TE buffer followed by 8 µl of RNaseA (Thermo Fisher EN0531; final concentration of 0.2 mg/ml) were added to each sample, which was then incubated for 2 h at 37°C. Four microliters Proteinase K (New England Biolabs P8107S; final concentration of 0.2 mg/ml) were added to each sample, which was then incubated for 2 h at 55°C. Four hundered microliters phenol:chloroform:isoamyl alcohol (25:24:1; Sigma, 77617) were added to each sample, which was then mixed with vortexing and centrifuged for 10 min at 13,500 *g* at 4°C to separate phases. The aqueous top layer was transferred to another tube and mixed with 30 µg glycogen (Thermo Fisher 10814) and 5 M NaCl (200 mM final concentration). Eight hundred microliters 100% EtOH at 4°C were added to each sample, which was then incubated at −20°C for 30 min. The DNA was pelleted by centrifugation for 10 min at 13,500 *g* at 4°C, washed with 500 µl 80% EtOH at 4°C, and centrifuged for 5 min at 13,500 *g* at 4°C. After removing the EtOH, the pellet was dried at 25°C and all DNA for each sample was resuspended in 30 µl 10 mM Tris–HCl, pH 8 total. The DNA concentration and average size of the sonicated fragments was determined using a DNA high sensitivity kit (Agilent 5067-4626) and the Agilent 2100 Bioanalyzer. Libraries for Illumina Next Generation Sequencing were prepared with the MicroPlex library preparation kit (Diagenode C05010014), with KAPA HiFi polymerase (KAPA biosystems) substituted for the PCR amplification. Libraries were sequenced on the NextSeq 500 platform (Illumina).

Sequenced reads (150 bp paired end) were mapped to the *P. falciparum* genome (Gardner *et al*, 2002; plasmoDB.org, version 3, release 55; Amos *et al*, 2021) using "bwa mem" (Li & Durbin, 2009) allowing a read to align only once to the reference genome (option "–c 1"). Alignments were subsequently filtered for duplicates and a mapping quality ≥ 20 using samtools (Li *et al*, 2009). The paired end

deduplicated ChIP and input BAM files were used as treatment and control, respectively, for peak calling with the macs2 command callpeak default settings (Zhang *et al*, 2008). Significant consensus peaks shared between clones A and B at each time point were defined using the bedtools intersect command (Quinlan & Hall, 2010). Consensus peaks with *q*-value cutoff 0.05 for each time point were visualized in Integrative Genomics Viewer (Robinson *et al*, 2011) along with ChIP-Input ratio coverage obtained from deeptool's bamCompare command (Ramírez *et al*, 2016).

SMC3 binding peaks were associated to the nearest protein-coding genes using bedtools closest command (Quinlan & Hall, 2010) along with the *P. falciparum* reference genome feature file (gff; plasmoDB.org, version 3, release 56; Amos *et al*, 2021). Only regions 500 bp upstream or downstream of the protein coding genes were considered further for downstream analysis. To perform functional analysis on the genes closest to the SMC3 bound peaks, the Gene Ontology Enrichment tool from PlasmoDB web interface (plasmoDB.org, version 3, release 56; Amos *et al*, 2021) was used for Ontology term – Biological Process with a *P*-Value cutoff of 0.05. Centromeric regions from Hoeijmakers *et al* (2012) were corrected for changes in genome annotation. These regions were overlapped with the SMC3 peaks dataset using the bedtools intersect command (Quinlan & Hall, 2010). Fold change quantification and statistical analysis for all peaks and peaks in centromeric regions was performed in R (R Core Team, 2021).

Significant SMC3 peaks shared between the two ChIP-seq replicates were overlapped with the convergent and divergent regions between genes using bedtools intersect (Quinlan & Hall, 2010) and peak enrichment values were extracted from the macs2 output.

Metagene plots were generated using the deeptool's suite (Ramírez *et al*, 2016). In brief, ChIP/Input ratios were calculated genome-wide using 'bamCompare' and normalized across regions of interest using 'computeMatrix'. Final matrices were plotted using 'plotProfile'. Processed bedgraph files of ATAC-seq data from (Data ref: Toenhake *et al*, 2018a; Toenhake *et al*, 2018b) were downloaded from the Gene Expression Ominbus (GSE104075) and processed as described above. Gene sets used for the metagene plots in Fig EV5E and F were based on data from (Data ref: Zanghì *et al*, 2018a; Zanghì *et al*, 2018b) and (Data ref: Baumgarten *et al*, 2019a; Baumgarten *et al*, 2019b).

RNA extraction, stranded RNA sequencing, and analysis

A WT and SMC3-3HA-*glmS* clone were synchronized simultaneously and each culture was split into two at 12 hpi. Glucosamine (Sigma G1514, final concentration 2.5 mM) was added to one half of the culture for two rounds of parasite replication (approximately 96 h). Parasites were then re-synchronized and three technical replicates (with and without glucosamine) were harvested at 12, 24, and 36 hpi. RBCs were lysed in 0.075% saponin (Sigma S7900) in PBS at 37°C, centrifuged at 3,250 *g* for 5 min, washed in PBS, centrifuged at 3,250 g for 5 min, and resuspended in 700 µl QIAzol reagent (Qiagen 79306). RNA was extracted using an miRNeasy Mini kit (Qiagen 1038703) with the recommended on-column DNase treatment. Total RNA was poly (A) selected using the Dynabeads mRNA Purification Kit (Thermo Fischer Scientific 61006). Library preparation was performed with the NEBNext® Ultra™ II Directional RNA Library Prep Kit for Illumina® (New England Biolabs E7760S) and paired end sequencing was performed on the Nextseq 550 platform (Illumina). Sequenced reads were mapped to the *P. falciparum* genome (plasmoDB.org, version 3, release 55; Amos *et al*, 2021) using "bwa mem" (Li & Durbin, 2009), allowing a read to align only once to the reference genome (option "–c 1"). Alignments were subsequently filtered for duplicates and a mapping quality ≥ 20 using samtools (Li *et al*, 2009). A single replicate from the untreated SMC3-3HA-*glmS* parasites at 12hpi was excluded due to low quality of the sequenced DNA library. Gene counts were quantified with htseq-count (Anders *et al*, 2015) and differentially expressed genes were identified in R (R Core Team, 2021) using package DESeq2 (Love *et al*, 2014). For filtering genes that were differentially expressed in WT parasites from SMC3-HA-*glmS* parasites at each time point, genes with a *q*-value < 0.01 in the WT were subtracted from the list of genes in SMC3-HA-*glmS* parasites with a *q*-value < 0.1. Gene Ontology enrichment analysis was performed on filtered differentially expressed genes ($q < 0.05$) using the built-in tool at PlasmoDB.org (version 3, release 56; Amos *et al*, 2021) with default settings for Biological Process (*P*-value < 0.05).

Estimation of cell cycle progression

RNA-seq-based cell cycle progression was estimated in R by comparing the normalized expression values (i.e., RPKM, reads per kilobase per exon per one million mapped reads) of each sample to the microarray data from (Data ref: Bozdech *et al*, 2003a; Bozdech *et al*, 2003b) using the statistical model as in Lemieux *et al* (2009).

Parasite growth assay

Parasite growth was measured as described previously (Vembar *et al*, 2015). Briefly, two SMC3-3HA-*glmS* clones and a WT culture were tightly synchronized. Each culture was split and glucosamine (Sigma G1514, 2.5 mM final concentration) was added to one half for approximately 96 h before starting the growth curve. The parasites were tightly re-synchronized and diluted to ~0.2% parasitemia (5% hematocrit) at ring stage. The growth curve was performed in a 96-well plate (200 µl culture per well) with three technical replicates per condition per blood. Every 24 h, 5 µl of the culture were fixed in 45 µl of 0.025% glutaraldehyde in PBS for 1 h at 4°C. After centrifuging at 800 *g* for 5 min, free aldehyde groups were quenched by re-suspending the iRBC pellet in 200 µl of 15 mM NH_4Cl in PBS. A 1:10 dilution of the quenched iRBC suspension was incubated with Sybr Green I (Sigma S9430) to stain the parasite nuclei. Quantification of the iRBCs was performed in a CytoFLEX S cytometer (Beckman Coulter), and analysis with was done with FlowJo Software. Growth rate was calculated and expressed as the parasitemia at Day X divided by the starting average parasitemia (Day 1) of the correspondent culture.

Graphics

The synopsis image for this manuscript was created with BioRender.com.

Data availability

All data sets generated in this study are available in the following databases:

© 2023 The Authors

- ChIP-seq and RNA-seq data: NCBI BioProject accession # PRJNA854331 (https://www.ncbi.nlm.nih.gov/bioproject/PRJNA854331)
- SMC3-3HA Proteomics data: PRIDE repository accession # PXD035225 (http://www.ebi.ac.uk/pride/archive/projects/PXD035225)

Expanded View for this article is available online.

Acknowledgements

This work was supported by the Agence Nationale de la Recherche (grant ANR-21-CE15-0010 PlasmoVarOrg to JMB and grant ANR-11-LABEX-0024-01 ParaFrap to ASc); the European Research Council (grant PlasmoSilencing 670301 to ASc and grant PlasmoEpiRNA 947819 to SB); Emerging Infectious Diseases junior seed grant from the Pasteur Institute to CR; the Academic Research Fund (Tier 2) of the Ministry of Education, Singapore (MOE2018-T2-2-131 to PRP); and the Merlion Project (6.11.18 to PRP). Work in the laboratories of PRP and PCD was funded by the National Research Foundation of Singapore through the Singapore-MIT Alliance for Research and Technology Antimicrobial Resistance Interdisciplinary Research Group. JMB was supported by a European Molecular Biology Organization long-term postdoctoral fellowship (EMBO ALTF 180-2015), the Institut Pasteur Roux-Cantarini postdoctoral fellowship, and a ParaFrap fellowship. SB was supported by a European Molecular Biology Organization long-term postdoctoral fellowship (EMBO ALTF 1444-2016) and advanced fellowship (EMBO aALTF 632-2018). PS was supported by the Institut Pasteur Roux-Cantarini postdoctoral fellowship. ASi was supported by the Singapore-MIT Alliance (SMA) Graduate Fellowship from the Ministry of Education of Singapore. The authors would like to acknowledge the use of the Biomics and Flow Cytometry platforms at the Institut Pasteur, as well as the mass spectrometer facilities at A*STAR Institute of Molecular and Cell Biology in the laboratory of Dr. Radoslaw Sabota with the aid of Dr. Wint Wint Phoo.

Author contributions

Catarina Rosa: Conceptualization; investigation; visualization; writing – original draft; writing – review and editing. **Parul Singh:** Conceptualization; data curation; formal analysis; validation; visualization; writing – original draft; writing – review and editing. **Patty Chen:** Investigation; visualization. **Ameya Sinha:** Investigation. **Aurélie Claës:** Investigation. **Peter R Preiser:** Supervision; funding acquisition. **Peter C Dedon:** Supervision; funding acquisition. **Sebastian Baumgarten:** Conceptualization; formal analysis; supervision; validation; visualization; writing – original draft; writing – review and editing. **Artur Scherf:** Supervision; funding acquisition. **Jessica M Bryant:** Conceptualization; formal analysis; supervision; funding acquisition; visualization; writing – original draft; project administration; writing – review and editing.

Disclosure and competing interest statement

The authors declare that they have no conflict of interest.

References

Amos B, Aurrecoechea C, Barba M, Barreto A, Basenko EY, Bażant W, Belnap R, Blevins AS, Böhme U, Brestelli J *et al* (2021) VEuPathDB: the eukaryotic pathogen, vector and host bioinformatics resource center. *Nucleic Acids Research* 50: D898 – D911. https://doi.org/10.1093/nar/gkab929

Anders S, Pyl PT, Huber W (2015) HTSeq-A Python framework to work with high-throughput sequencing data. *Bioinformatics* 31: 166 – 169

Armstrong CM, Goldberg DE (2007) An FKBP destabilization domain modulates protein levels in *Plasmodium falciparum*. *Nat Methods* 4: 1007 – 1009

Arnot DE, Ronander E, Bengtsson DC (2011) The progression of the intra-erythrocytic cell cycle of *Plasmodium falciparum* and the role of the centriolar plaques in asynchronous mitotic division during schizogony. *Int J Parasitol* 41: 71 – 80

Aurrecoechea C, Barreto A, Basenko EY, Brestelli J, Brunk BP, Cade S, Crouch K, Doherty R, Falke D, Fischer S *et al* (2017) EuPathDB: the eukaryotic pathogen genomics database resource. *Nucleic Acids Res* 45: D581 – D591

Ay F, Bunnik EM, Varoquaux N, Bol SM, Prudhomme J, Vert JP, Noble WS, Le Roch KG (2014) Three-dimensional modeling of the *P. falciparum* genome during the erythrocytic cycle reveals a strong connection between genome architecture and gene expression. *Genome Res* 24: 974 – 988

Balaji S, Madan Babu M, Iyer LM, Aravind L (2005) Discovery of the principal specific transcription factors of Apicomplexa and their implication for the evolution of the AP2-integrase DNA binding domains. *Nucleic Acids Res* 33: 3994 – 4006

Batsios P, Peter T, Baumann O, Stick R, Meyer I, Gräf R (2012) A lamin in lower eukaryotes? *Nucleus* 3: 237 – 243

Batugedara G, Lu XM, Saraf A, Sardiu ME, Cort A, Abel S, Prudhomme J, Washburn MP, Florens L, Bunnik EM *et al* (2020) The chromatin bound proteome of the human malaria parasite. *Microb Genom* 6: e000327

Baumgarten S, Bryant JM, Sinha A, Reyser T, Preiser PR, Dedon PC, Scherf A (2019a) Gene Expression Omnibus GSE123839 (https://www.ncbi.nlm.nih.gov/geo/query/acc.cgi?acc=GSE123839). [DATASET]

Baumgarten S, Bryant JM, Sinha A, Reyser T, Preiser PR, Dedon PC, Scherf A (2019b) Transcriptome-wide dynamics of extensive m6A mRNA methylation during *Plasmodium falciparum* blood-stage development. *Nat Microbiol* 4: 2246 – 2259

Baumgarten S, Bryant J, Chromatin JB (2022a) BioSample SAMN27914429 (https://www.ncbi.nlm.nih.gov/biosample/?term=SAMN27914429). [DATASET]

Baumgarten S, Bryant J, Chromatin JB (2022b) Chromatin structure can introduce systematic biases in genome-wide analyses of *Plasmodium falciparum*. *Open Res Europe* 2: 75

Birnbaum J, Flemming S, Reichard N, Soares AB, Mesén-Ramírez P, Jonscher E, Bergmann B, Spielmann T (2017) A genetic system to study *Plasmodium falciparum* protein function. *Nat Methods* 14: 450 – 456

Bozdech Z, Llinás M, Pulliam BL, Wong ED, Zhu J & DeRisi JL (2003a) (https://doi.org/10.1371/journal.pbio.0000005.sd002). [DATASET]

Bozdech Z, Llinás M, Pulliam BL, Wong ED, Zhu J, DeRisi JL (2003b) The transcriptome of the intraerythrocytic developmental cycle of *Plasmodium falciparum*. *PLoS Biol* 1: e5

Bunnik EM, Cook KB, Varoquaux N, Batugedara G, Prudhomme J, Cort A, Shi L, Andolina C, Ross LS, Brady D *et al* (2018) Changes in genome organization of parasite-specific gene families during the Plasmodium transmission stages. *Nat Commun* 9: 1910

Bunnik EM, Venkat A, Shao J, McGovern KE, Batugedara G, Worth D, Prudhomme J, Lapp SA, Andolina C, Ross LS *et al* (2019) Comparative 3D genome organization in apicomplexan parasites. *Proc Natl Acad Sci U S A* 116: 3183 – 3192

Campbell TL, de Silva EK, Olszewski KL, Elemento O, Llinás M (2010) Identification and genome-wide prediction of DNA binding specificities for the ApiAP2 family of regulators from the malaria parasite. *PLoS Pathog* 6: e1001165

© 2023 The Authors

Caro F, Ahyong V, Betegon M, DeRisi JL (2014) Genome-wide regulatory dynamics of translation in the *Plasmodium falciparum* asexual blood stages. *Elife* 3: e04106

Carvalhal S, Tavares A, Santos MB, Mirkovic M, Oliveira RA (2018) A quantitative analysis of cohesin decay in mitotic fidelity. *J Cell Biol* 217: 3343 – 3353

Chen PB, Ding S, Zanghì G, Soulard V, DiMaggio PA, Fuchter MJ, Mecheri S, Mazier D, Scherf A, Malmquist NA (2016) *Plasmodium falciparum* PfSET7: enzymatic characterization and cellular localization of a novel protein methyltransferase in sporozoite, liver and erythrocytic stage parasites. *Sci Rep* 6: 21802

Cowman AF, Berry D, Baum J (2012) The cellular and molecular basis for malaria parasite invasion of the human red blood cell. *J Cell Biol* 198: 961 – 971

Cowman AF, Healer J, Marapana D, Marsh K (2016) Malaria: biology and disease. *Cell* 167: 610 – 624

Cowman AF, Tonkin CJ, Tham WH, Duraisingh MT (2017) The molecular basis of erythrocyte invasion by malaria parasites. *Cell Host Microbe* 22: 232 – 245

Davidson IF, Bauer B, Goetz D, Tang W, Wutz G, Peters J-M (2019) DNA loop extrusion by human cohesin. *Science* 366: 1338 – 1345

Dekker J, Heard E (2015) Structural and functional diversity of topologically associating domains. *FEBS Lett* 589: 2877 – 2884

Dixon JR, Selvaraj S, Yue F, Kim A, Li Y, Shen Y, Hu M, Liu JS, Ren B (2012) Topological domains in mammalian genomes identified by analysis of chromatin interactions. *Nature* 485: 376 – 380

Dixon JR, Gorkin DU, Ren B (2016) Chromatin domains: the unit of chromosome organization. *Mol Cell* 62: 668 – 680

Dorsett D, Ström L (2012) The ancient and evolving roles of cohesin in gene expression and DNA repair. *Curr Biol* 22: R240 – R250

Eichinger CS, Kurze A, Oliveira RA, Nasmyth K (2013) Disengaging the Smc3/ kleisin interface releases cohesin from *Drosophila* chromosomes during interphase and mitosis. *EMBO J* 32: 656 – 665

Ganter M, Goldberg JM, Dvorin JD, Paulo JA, King JG, Tripathi AK, Paul AS, Yang J, Coppens I, Jiang RHY *et al* (2017) *Plasmodium falciparum* CRK4 directs continuous rounds of DNA replication during schizogony. *Nat Microbiol* 2: 17017

Gardner MJ, Hall N, Fung E, White O, Berriman M, Hyman RW, Carlton JM, Pain A, Nelson KE, Bowman S *et al* (2002) Genome sequence of the human malaria parasite *Plasmodium falciparum*. *Nature* 419: 498 – 511

Gerlich D, Koch B, Dupeux F, Peters JM, Ellenberg J (2006) Live-cell imaging reveals a stable cohesin-chromatin interaction after but not before DNA replication. *Curr Biol* 16: 1571 – 1578

Ghorbal M, Gorman M, MacPherson CR, Martins RM, Scherf A, Lopez-Rubio JJ (2014) Genome editing in the human malaria parasite *Plasmodium falciparum* using the CRISPR-Cas9 system. *Nat Biotechnol* 32: 819 – 821

Gligoris TG, Scheinost JC, Bürmann F, Petela N, Chan KL, Uluocak P, Beckouët F, Gruber S, Nasmyth K, Löwe J (2014) Closing the cohesin ring: structure and function of its Smc3-kleisin interface. *Science* 346: 963 – 967

Haase S, Cabrera A, Langer C, Treeck M, Struck N, Herrmann S, Jansen PW, Bruchhaus I, Bachmann A, Dias S *et al* (2008) Characterization of a conserved rhoptry-associated leucine zipper-like protein in the malaria parasite *Plasmodium falciparum*. *Infect Immun* 76: 879 – 887

Heger P, Marin B, Bartkuhn M, Schierenberg E, Wiehe T (2012) The chromatin insulator CTCF and the emergence of metazoan diversity. *Proc Natl Acad Sci U S A* 109: 17507 – 17512

Heidinger-Pauli JM, Mert O, Davenport C, Guacci V, Koshland D (2010) Systematic reduction of cohesin differentially affects chromosome segregation, condensation, and DNA repair. *Curr Biol* 20: 957 – 963

Hillier C, Pardo M, Yu L, Bushell E, Sanderson T, Metcalf T, Herd C, Anar B, Rayner JC, Billker O *et al* (2019) Landscape of the Plasmodium interactome reveals both conserved and species-specific functionality. *Cell Rep* 28: 1635 – 1647

Hoeijmakers WAM, Flueck C, Françoijs KJ, Smits AH, Wetzel J, Volz JC, Cowman AF, Voss T, Stunnenberg HG, Bártfai R (2012) *Plasmodium falciparum* centromeres display a unique epigenetic makeup and cluster prior to and during schizogony. *Cell Microbiol* 14: 1391 – 1401

Holzmann J, Politi AZ, Nagasaka K, Hantsche-Grininger M, Walther N, Koch B, Fuchs J, Dürnberger G, Tang W, Ladurner R *et al* (2019) Absolute quantification of cohesin, CTCF and their regulators in human cells. *Elife* 8: e46269

Hu G, Cabrera A, Kono M, Mok S, Chaal BK, Haase S, Engelberg K, Cheemadan S, Spielmann T, Preiser PR *et al* (2010) Transcriptional profiling of growth perturbations of the human malaria parasite *Plasmodium falciparum*. *Nat Biotechnol* 28: 91 – 98

Hu B, Itoh T, Mishra A, Katoh Y, Chan KL, Upcher W, Godlee C, Roig MB, Shirahige K, Nasmyth K (2011) ATP hydrolysis is required for relocating cohesin from sites occupied by its Scc2/4 loading complex. *Curr Biol* 21: 12 – 24

Huis in 't Veld P, Herzog F, Ladurner R, Davidson IF, Piric S, Kreidl E, Bhaskara V, Aebersold R, Peters J-M (2014) Characterization of a DNA exit gate in the human cohesin ring. *Science* 346: 968 – 972

Kagey MH, Newman JJ, Bilodeau S, Zhan Y, Orlando DA, Van Berkum NL, Ebmeier CC, Goossens J, Rahl PB, Levine SS *et al* (2010) Mediator and cohesin connect gene expression and chromatin architecture. *Nature* 467: 430 – 435

Kensche PR, Hoeijmakers WAM, Toenhake CG, Bras M, Chappell L, Berriman M, Bártfai R (2016) The nucleosome landscape of *Plasmodium falciparum* reveals chromatin architecture and dynamics of regulatory sequences. *Nucleic Acids Res* 44: 2110 – 2124

Kim Y, Shi Z, Zhang H, Finkelstein IJ, Yu H (2019) Human cohesin compacts DNA by loop extrusion. *Science* 366: 1345 – 1349

Klaus S, Binder P, Kim J, Machado M, Funaya C, Schaaf V, Klaschka D, Kudulyte A, Cyrklaff M, Laketa V *et al* (2022) Asynchronous nuclear cycles in multinucleated *Plasmodium falciparum* facilitate rapid proliferation. *Sci Adv* 8: 5362

Kothiwal D, Laloraya S (2019) A SIR-independent role for cohesin in subtelomeric silencing and organization. *Proc Natl Acad Sci U S A* 116: 5659 – 5664

Kubo N, Ishii H, Xiong X, Bianco S, Meitinger F, Hu R, Hocker JD, Conte M, Gorkin D, Yu M *et al* (2021) Promoter-proximal CTCF binding promotes distal enhancer-dependent gene activation. *Nat Struct Mol Biol* 28: 152 – 161

Lemieux JE, Gomez-Escobar N, Feller A, Carret C, Amambua-Ngwa A, Pinches R, Day F, Kyes SA, Conway DJ, Holmes CC *et al* (2009) Statistical estimation of cell-cycle progression and lineage commitment in *Plasmodium falciparum* reveals a homogeneous pattern of transcription in *ex vivo* culture. *Proc Natl Acad Sci U S A* 106: 7559 – 7564

Lengronne A, Katou Y, Mori S, Yokabayashi S, Kelly GP, Ito T, Watanabe Y, Shirahige K, Uhlmann F (2004) Cohesin relocation from sites of chromosomal loading to places of convergent transcription. *Nature* 430: 573 – 578

Li H, Durbin R (2009) Fast and accurate short read alignment with Burrows-Wheeler transform. *Bioinformatics* 25: 1754 – 1760

Li H, Handsaker B, Wysoker A, Fennell T, Ruan J, Homer N, Marth G, Abecasis G, Durbin R (2009) The Sequence Alignment/Map format and SAMtools. *Bioinformatics* 25: 2078 – 2079

 © 2023 The Authors

Lopez-Rubio JJ, Mancio-Silva L, Scherf A (2009) Genome-wide analysis of heterochromatin associates clonally variant gene regulation with perinuclear repressive centers in malaria parasites. *Cell Host Microbe* 5: 179 – 190

Love MI, Huber W, Anders S (2014) Moderated estimation of fold change and dispersion for RNA-seq data with DESeq2. *Genome Biol* 15: 550

Lu B, Liu M, Gu L, Li Y, Shen S, Guo G, Wang F, He X, Zhao Y, Shang X *et al* (2021) The architectural factor hmgb1 is involved in genome organization in the human malaria parasite *Plasmodium falciparum*. *MBio* 12: e00148-21

MacPherson CR, Scherf A (2015) Flexible guide-RNA design for CRISPR applications using Protospacer Workbench. *Nat Biotechnol* 33: 805 – 806

Matthews H, Duffy CW, Merrick CJ (2018) Checks and balances? DNA replication and the cell cycle in Plasmodium. *Parasites & Vectors* 11: 216. https://doi.org/10.1186/s13071-018-2800-1

Mehnert A-K, Simon CS, Guizetti J (2019) Immunofluorescence staining protocol for STED nanoscopy of Plasmodium-infected red blood cells. *Mol Biochem Parasitol* 229: 47 – 52

Mesén-Ramírez P, Reinsch F, Blancke Soares A, Bergmann B, Ullrich AK, Tenzer S, Spielmann T (2016) Stable translocation intermediates jam global protein export in *Plasmodium falciparum* parasites and link the PTEX component EXP2 with translocation activity. *PLoS Pathog* 12: e1005618

Michaelis C, Ciosk R, Nasmyth K (1997) Cohesins: chromosomal proteins that prevent premature separation of sister chromatids. *Cell* 91: 35 – 45

Mirkovic M, Oliveira RA (2017) Centromeric cohesin: molecular glue and much more. *Prog Mol Subcell Biol* 485 – 513

Mistry J, Chuguransky S, Williams L, Qureshi M, Salazar GA, Sonnhammer ELL, Tosatto SCE, Paladin L, Raj S, Richardson LJ *et al* (2021) Pfam: the protein families database in 2021. *Nucleic Acids Res* 49: D412 – D419

Muller H, Gil J, Drinnenberg IA (2019) The impact of centromeres on spatial genome architecture. *Trends Genet* 35: 565 – 578

Murayama Y, Uhlmann F (2014) Biochemical reconstitution of topological DNA binding by the cohesin ring. *Nature* 505: 367 – 371

Nativio R, Wendt KS, Ito Y, Huddleston JE, Uribe-Lewis S, Woodfine K, Krueger C, Reik W, Peters JM, Murrell A (2009) Cohesin is required for higher-order chromatin conformation at the imprinted IGF2-H19 locus. *PLoS Genet* 5: e1000739

Nuebler J, Fudenberg G, Imakaev M, Abdennur N, Mirny LA (2018) Chromatin organization by an interplay of loop extrusion and compartmental segregation. *Proc Natl Acad Sci U S A* 115: E6697 – E6706

O'Donnell RA, Saul A, Cowman AF, Crabb BS (2000) Functional conservation of the malaria vaccine antigen MSP-119 across distantly related *Plasmodium* species. *Nat Med* 6: 91 – 95

O'Donnell RA, de Koning-Ward TF, Burt RA, Bockarie M, Reeder JC, Cowman AF, Crabb BS (2001) Antibodies against Merozoite surface protein (Msp)-119 are a major component of the invasion-inhibitory response in individuals immune to malaria. *J Exp Med* 193: 1403 – 1412

Oh S, Shao J, Mitra J, Xiong F, D'Antonio M, Wang R, Garcia-Bassets I, Ma Q, Zhu X, Lee JH *et al* (2021) Enhancer release and retargeting activates disease-susceptibility genes. *Nature* 595: 735 – 740

Painter HJ, Chung NC, Sebastian A, Albert I, Storey JD, Llinás M (2018a) Gene Expression Omnibus GSE66669 (https://www.ncbi.nlm.nih.gov/geo/query/acc.cgi?acc=GSE66669). [DATASET]

Painter HJ, Chung NC, Sebastian A, Albert I, Storey JD, Llinás M (2018b) Genome-wide real-time *in vivo* transcriptional dynamics during *Plasmodium falciparum* blood-stage development. *Nat Commun* 9: 2656

Perea-Resa C, Wattendorf L, Marzouk S, Blower MD (2021) Cohesin: behind dynamic genome topology and gene expression reprogramming. *Trends Cell Biol* 31: 760 – 773

Peters JM, Nishiyama T (2012) Sister chromatid cohesion. *Cold Spring Harb Perspect Biol* 4: a011130

Prommana P, Uthaipibull C, Wongsombat C, Kamchonwongpaisan S, Yuthavong Y, Knuepfer E, Holder AA, Shaw PJ (2013) Inducible knockdown of *Plasmodium* gene expression using the glmS ribozyme. *PloS One* 8: e73783

Quinlan AR, Hall IM (2010) BEDTools: a flexible suite of utilities for comparing genomic features. *Bioinformatics* 26: 841 – 842

R Core Team (2021) *R: A language and environment for statistical computing*. Vienna: R Foundation for Statistical Computing

Ralph SA, Scheidig-Benatar C, Scherf A (2005) Antigenic variation in *Plasmodium falciparum* is associated with movement of var loci between subnuclear locations. *Proc Natl Acad Sci U S A* 102: 5414 – 5419

Ramírez F, Ryan DP, Grüning B, Bhardwaj V, Kilpert F, Richter AS, Heyne S, Dündar F, Manke T (2016) deepTools2: a next generation web server for deep-sequencing data analysis. *Nucleic Acids Res* 44: 160 – 165

Rao SSP, Huang SC, Glenn St Hilaire B, Engreitz JM, Perez EM, Kieffer-Kwon KR, Sanborn AL, Johnstone SE, Bascom GD, Bochkov ID *et al* (2017) Cohesin loss eliminates all loop domains. *Cell* 171: 305 – 320

Robinson JT, Thorvaldsdóttir H, Winckler W, Guttman M, Lander ES, Getz G, Mesirov JP (2011) Integrative genomics viewer. *Nat Biotechnol* 29: 24 – 26

Rudlaff RM, Kraemer S, Marshman J, Dvorin JD (2020) Three-dimensional ultrastructure of *Plasmodium falciparum* throughout cytokinesis. *PLoS Pathog* 16: e1008587

Salcedo-Amaya AM, Van Driel MA, Alako BT, Trelle MB, Van Den Elzen AMG, Cohen AM, Janssen-Megens EM, Van De Vegte-Bolmer M, Selzer RR, Iniguez AL *et al* (2009) Dynamic histone H3 epigenome marking during the intraerythrocytic cycle of *Plasmodium falciparum*. *Proc Natl Acad Sci U S A* 106: 9655 – 9660

Santos JM, Josling G, Ross P, Joshi P, Campbell T, Schieler A, Cristea IM (2017) Red blood cell invasion by the malaria parasite is coordinated by the PfAP2-I transcription factor. *Cell Host Microbe* 21: 731 – 741

Sasca D, Yun H, Giotopoulos G, Szybinski J, Evan T, Wilson NK, Gerstung M, Gallipoli P, Green AR, Hills R *et al* (2019) Cohesin-dependent regulation of gene expression during differentiation is lost in cohesin-mutated myeloid malignancies. *Blood* 134: 2195 – 2208

Scherf A, Lopez-Rubio JJ, Riviere L (2008) Antigenic variation in *Plasmodium falciparum*. *Annu Rev Microbiol* 62: 445 – 470

Sherling ES, Knuepfer E, Brzostowski JA, Miller LH, Blackman MJ, Van Ooij C (2017) The *Plasmodium falciparum* rhoptry protein RhopH3 plays essential roles in host cell invasion and nutrient uptake. *Elife* 6: e23239

Stanojcic S, Kuk N, Ullah I, Sterkers Y, Merrick CJ (2017) Single-molecule analysis reveals that DNA replication dynamics vary across the course of schizogony in the malaria parasite *Plasmodium falciparum*. *Sci Rep* 7: 4003

Tanaka T, Cosma MP, Wirth K, Nasmyth K (1999) Identification of cohesin association sites at centromeres and along chromosome arms. *Cell* 98: 847 – 858

Toenhake CG, Fraschka SA-K, Vijayabaskar MS, Westhead DR, van Heeringen SJ, Bártfai R (2018a) Gene Expression Omnibus GSE104075 (https://www.ncbi.nlm.nih.gov/geo/query/acc.cgi?acc=GSE104075). [DATASET]

Toenhake CG, Fraschka SA-K, Vijayabaskar MS, Westhead DR, van Heeringen SJ, Bártfai R (2018b) Chromatin accessibility-based characterization of the gene regulatory network underlying *Plasmodium falciparum* blood-stage development. *Cell Host Microbe* 23: 557 – 569

Tolhuis B, Blom M, Kerkhoven RM, Pagie L, Teunissen H, Nieuwland M, Simonis M, de Laat W, van Lohuizen M, van Steensel B (2011) Interactions among polycomb domains are guided by chromosome architecture. *PLoS Genet* 7: e1001343

© 2023 The Authors

Tomonaga T, Nagao K, Kawasaki Y, Furuya K, Murakaini A, Morishita J, Yuasa T, Sutani T, Kearsey SE, Uhlmann F *et al* (2000) Characterization of fission yeast cohesin: essential anaphase proteolysis of Rad21 phosphorylated in the S phase. *Genes Dev* 14: 2757 – 2770

Tonkin CJ, van Dooren GG, Spurck TP, Struck NS, Good RT, Handman E, Cowman AF, McFadden GI (2004) Localization of organellar proteins in *Plasmodium falciparum* using a novel set of transfection vectors and a new immunofluorescence fixation method. *Mol Biochem Parasitol* 137: 13 – 21

Trelle MB, Salcedo-Amaya AM, Cohen AM, Stunnenberg HG, Jensen ON (2009) Global histone analysis by mass spectrometry reveals a high content of acetylated lysine residues in the malaria parasite *Plasmodium falciparum*. *J Proteome Res* 8: 3439 – 3450

Uhlmann F (2016) SMC complexes: from DNA to chromosomes. *Nat Rev Mol Cell Biol* 17: 399 – 412

Vembar SS, Macpherson CR, Sismeiro O, Coppée JY, Scherf A (2015) The PfAlba1 RNA-binding protein is an important regulator of translational timing in *Plasmodium falciparum* blood stages. *Genome Biol* 16: 212

Wendt KS, Yoshida K, Itoh T, Bando M, Koch B, Schirghuber E, Tsutsumi S, Nagae G, Ishihara K, Mishiro T *et al* (2008) Cohesin mediates transcriptional insulation by CCCTC-binding factor. *Nature* 451: 796 – 801

World Health Organization (2020) *World malaria report 2020 – 20 years of global progress & challenges*. Geneva: World Health Organization

Wutz G, Várnai C, Nagasaka K, Cisneros DA, Stocsits RR, Tang W, Schoenfelder S, Jessberger G, Muhar M, Hossain MJ *et al* (2017) Topologically associating domains and chromatin loops depend on cohesin and are regulated by CTCF, WAPL, and PDS5 proteins. *EMBO J* 36: 3573 – 3599

Young JA, Johnson JR, Benner C, Yan SF, Chen K, Le Roch KG, Zhou Y, Winzeler EA (2008) In silico discovery of transcription regulatory elements in *Plasmodium falciparum*. *BMC Genomics* 9: 70

Zanghì G, Vembar SS, Baumgarten S, Ding S, Guizetti J, Bryant JM, Mattei D, Jensen ATR, Rénia L, Goh YS *et al* (2018a) BioSample SAMN06209423 (https://www.ncbi.nlm.nih.gov/biosample/?term=SAMN06209423). [DATASET]

Zanghì G, Vembar SS, Baumgarten S, Ding S, Guizetti J, Bryant JM, Mattei D, Jensen ATR, Rénia L, Goh YS *et al* (2018b) A specific PfEMP1 is expressed in *P. falciparum* sporozoites and plays a role in hepatocyte infection. *Cell Rep* 22: 2951 – 2963

Zhang Y, Liu T, Meyer CA, Eeckhoute J, Johnson DS, Bernstein BE, Nussbaum C, Myers RM, Brown M, Li W *et al* (2008) Model-based analysis of ChIP-Seq (MACS). *Genome Biol* 9: R137

Zhang Q, Huang Y, Zhang Y, Fang X, Claes A, Duchateau M, Namane A, Lopez-Rubio JJ, Pan W, Scherf A (2011) A critical role of perinuclear filamentous actin in spatial repositioning and mutually exclusive expression of virulence genes in malaria parasites. *Cell Host Microbe* 10: 451 – 463

License: This is an open access article under the terms of the Creative Commons Attribution License, which permits use, distribution and reproduction in any medium, provided the original work is properly cited.

Expanded View Figures

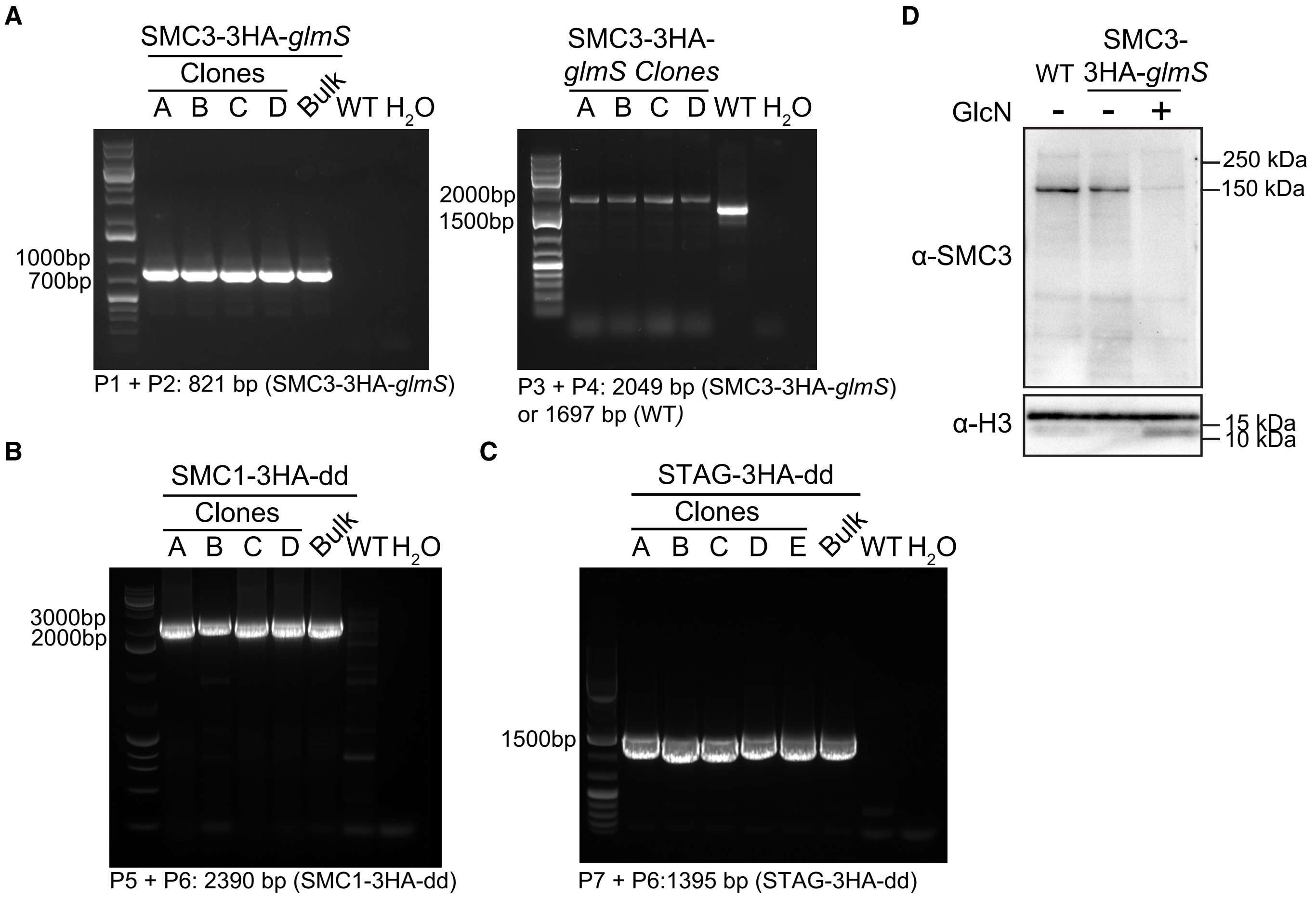

Figure EV1. Validation of strains and SMC3 antibody.

A DNA gels showing PCR validation of the SMC3-3HA-*glmS* strain with the indicated primers (Dataset EV22) shows integration of the 3HA-*glmS*-encoding sequence at the 3′ end of the endogenous *smc3* gene in the bulk transfection parasite population, as well as clones used in this study. No genomic DNA (H_2O) and genomic DNA from WT parasites (WT) are used as controls. DNA size is indicated with a ladder at the left side of each gel, and expected band sizes are indicated at the bottom of each gel.

B,C DNA gels showing PCR validation of the SMC1-3HA-dd (B) and STAG-3HA-dd (C) strains. PCR with the indicated primers (Dataset EV22) shows integration of the 3HA-dd-encoding sequence at the 3′ end of the endogenous *smc1* and *stag* genes in the bulk transfection parasite population and clones. No genomic DNA (H_2O) and genomic DNA from WT parasites (WT) are used as controls. DNA size is indicated with a ladder at the left side of each gel, and expected band sizes are indicated at the bottom of each gel.

D Western blot analysis of nuclear extracts from synchronous clonal populations of WT and SMC3-3HA-*glmS* trophozoite parasites in the absence (−) or presence (+) of glucosamine (GlcN). SMC3 is detected with an in-house generated anti-SMC3 antibody. An antibody against histone H3 is used as a loading control. Molecular weights are shown to the right.

© 2023 The Authors

Figure EV2. Extended SMC3-3HA-glmS ChIP-seq analysis.

A Venn diagrams showing overlap between significant ChIP-seq peaks called with macs2 (q-value < 0.05, Dataset EV4) from clones A and B at 12, 24, and 36 hpi.
B Circos plots of ChIP-seq data from clone B showing genome-wide SMC3 binding across the IDC. For 12 (blue), 24 (coral), and 36 (green) hpi, the 14 chromosomes are represented circularly by the outer gray bars, with chromosome number indicated in roman numerals and chromosome distances (Mbp) indicated in Arabic numerals. Enrichment ratio (ChIP/input) is shown as average reads per million (RPM) over bins of 1,000 nucleotides. The maximum y-axis value is 50 for 12 and 24 hpi and 80 for 36 hpi. Centromeric regions are represented by red bars in the innermost circle.
C Zoomed-in view of SMC3 ChIP-seq data from clone B corresponding to chromosome 4 (604,345–709,167 bp), including the centromere (represented with dark red line below the x-axis). For 12 (blue), 24 (coral), and 36 (green) hpi, the y-axis is enrichment (ChIP/Input), with vertical lines below representing significant peaks obtained from peak calling algorithm macs2 (present in both clones, q-value < 0.05). The x-axis is DNA sequence, with genes represented by black boxes indented to delineate introns and labeled with white arrowheads to indicate transcription direction.
D Plot comparing SMC3 peak enrichment [$\log_2$ (ChIP/Input)] in regions between convergent ("Converg.") and divergent ("Diverg.") genes at 12, 24, and 36 hpi (defined in Dataset EV6). Data shown are for consensus peaks between clone A and B that were called with macs2 (q-value < 0.05, Dataset EV4). Center black dot, median; central vertical line, standard deviation.

 © 2023 The Authors

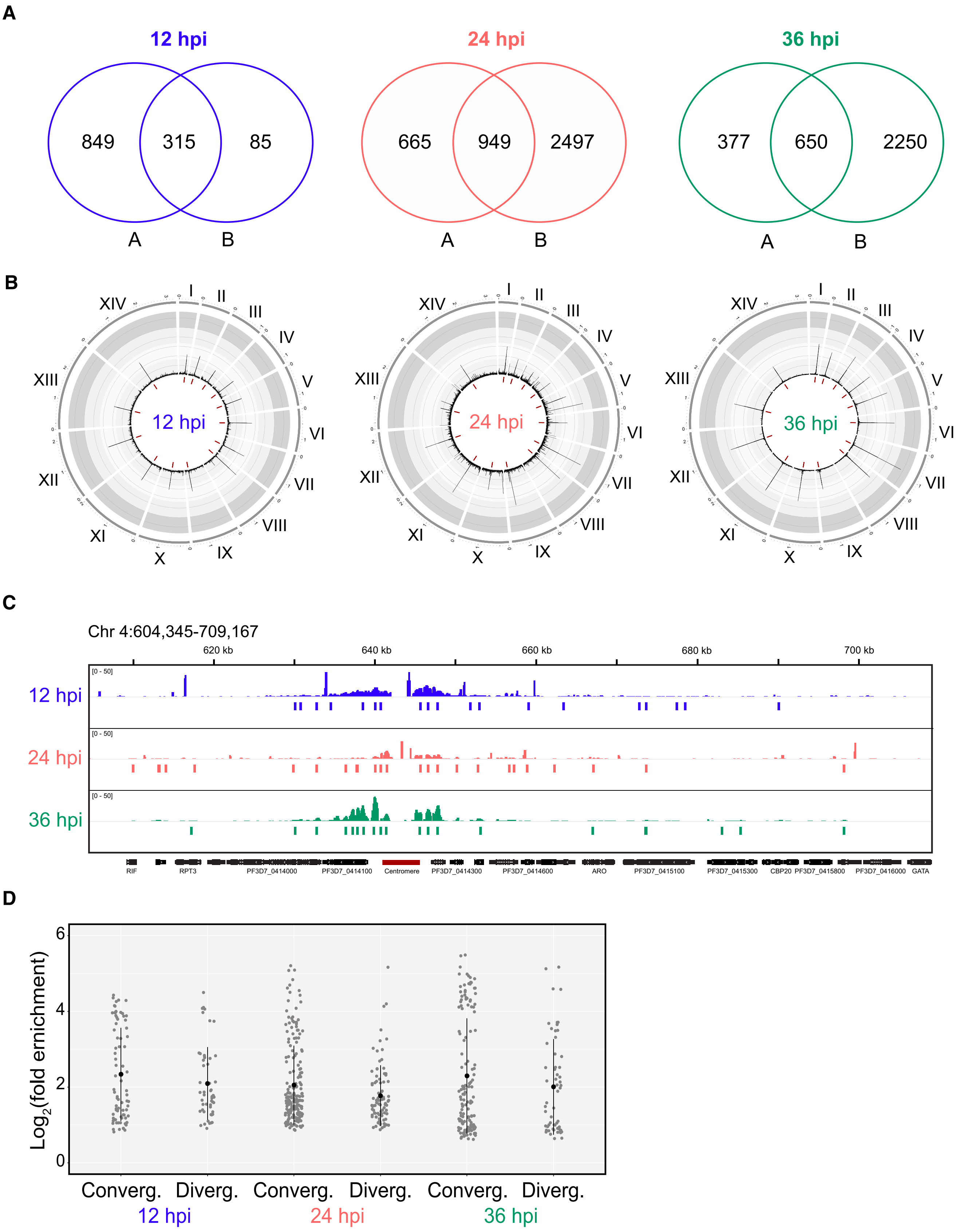
A
12 hpi
849
315
85
A
B
24 hpi
665
949
2497
A
B
36 hpi
377
650
2250
A
B
B
12 hpi
24 hpi
36 hpi
I
II
III
IV
V
VI
VII
VIII
IX
X
XI
XII
XIII
XIV
C
Chr 4:604,345-709,167
620 kb
640 kb
660 kb
680 kb
700 kb
12 hpi
24 hpi
36 hpi
[0 - 50]
RIF
RPT3
PF3D7_0414000
PF3D7_0414100
Centromere
PF3D7_0414300
PF3D7_0414600
ARO
PF3D7_0415100
PF3D7_0415300
CBP20
PF3D7_0415800
PF3D7_0416000
GATA
D
Log2(fold ernichment)
0
2
4
6
Converg.
Diverg.
Converg.
Diverg.
Converg.
Diverg.
12 hpi
24 hpi
36 hpi

Figure EV2.

Figure EV3. Analysis of SMC3-3HA-glmS knockdown.

A Western blot analysis of nuclear extracts from a synchronous clonal population of SMC3-3HA-*glmS* ring stage parasites in the absence (−) or presence (+) of glucosamine (GlcN) for 48 and 96 h (one and two IDC cycles, respectively). SMC3-3HA is detected with an anti-HA antibody. An antibody against histone H3 is used as a control. Molecular weights are shown to the right.

B RNA-seq of a WT and SMC3-3HA-*glmS* clone shows *smc3* transcript levels (FPKM) at 12, 24, and 36 hpi in the absence of glucosamine. Circles represent technical replicates of WT parasites and squares represent technical replicates of SMC3-3HA-*glmS* parasites. Asterisk indicates significance ($P < 0.05$).

C Giemsa-stained synchronous, clonal WT and SMC3-3HA-*glmS* parasite cultures in the absence (−GlcN) or presence (+GlcN) of glucosamine at the time points harvested for RNA-seq: 12, 24, and 36 hpi. Scale bar equals 10 μm.

D Cell cycle progression (hours post invasion on *x*-axis) estimation of synchronous, clonal WT and SMC3-3HA-*glmS* populations in the absence or presence of glucosamine (GlcN). RNA-seq data from synchronized parasites harvested at 12 (blue), 24 (coral), and 36 (green) hpi were compared to microarray data from (Data ref: Bozdech *et al*, 2003a; Bozdech *et al*, 2003b) as in (Lemieux *et al*, 2009) to determine the approximate time point in the IDC (*x*-axis). Replicates are represented with filled (−GlcN) or empty (+GlcN) circles.

 © 2023 The Authors

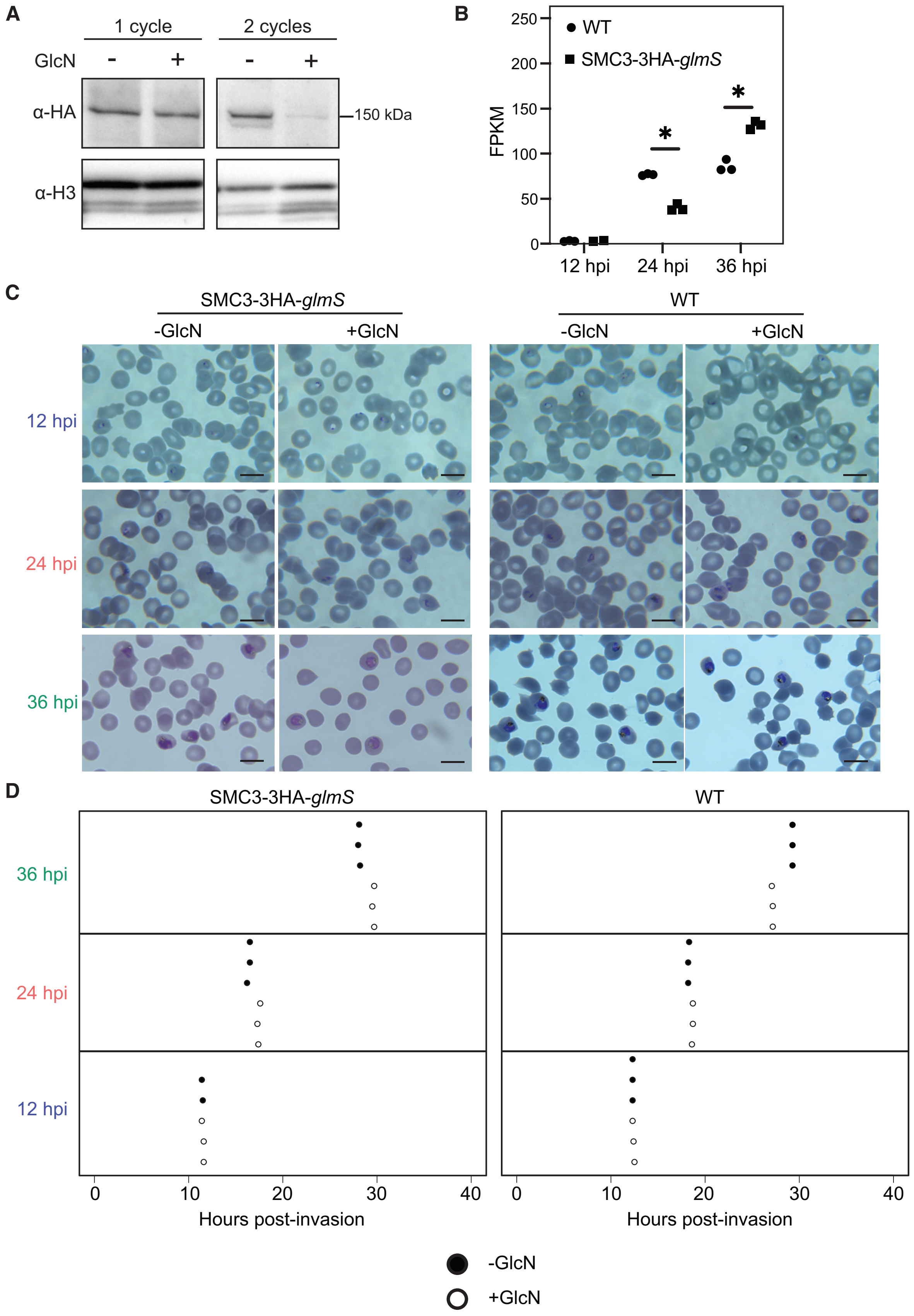
A
1 cycle
2 cycles
GlcN - + - +
α-HA
α-H3
150 kDa
B
FPKM
WT
SMC3-3HA-glmS
250
200
150
100
50
0
12 hpi
24 hpi
36 hpi
C
SMC3-3HA-glmS
WT
-GlcN
+GlcN
12 hpi
24 hpi
36 hpi
D
SMC3-3HA-glmS
WT
Hours post-invasion
0
10
20
30
40
-GlcN
+GlcN

Figure EV3.

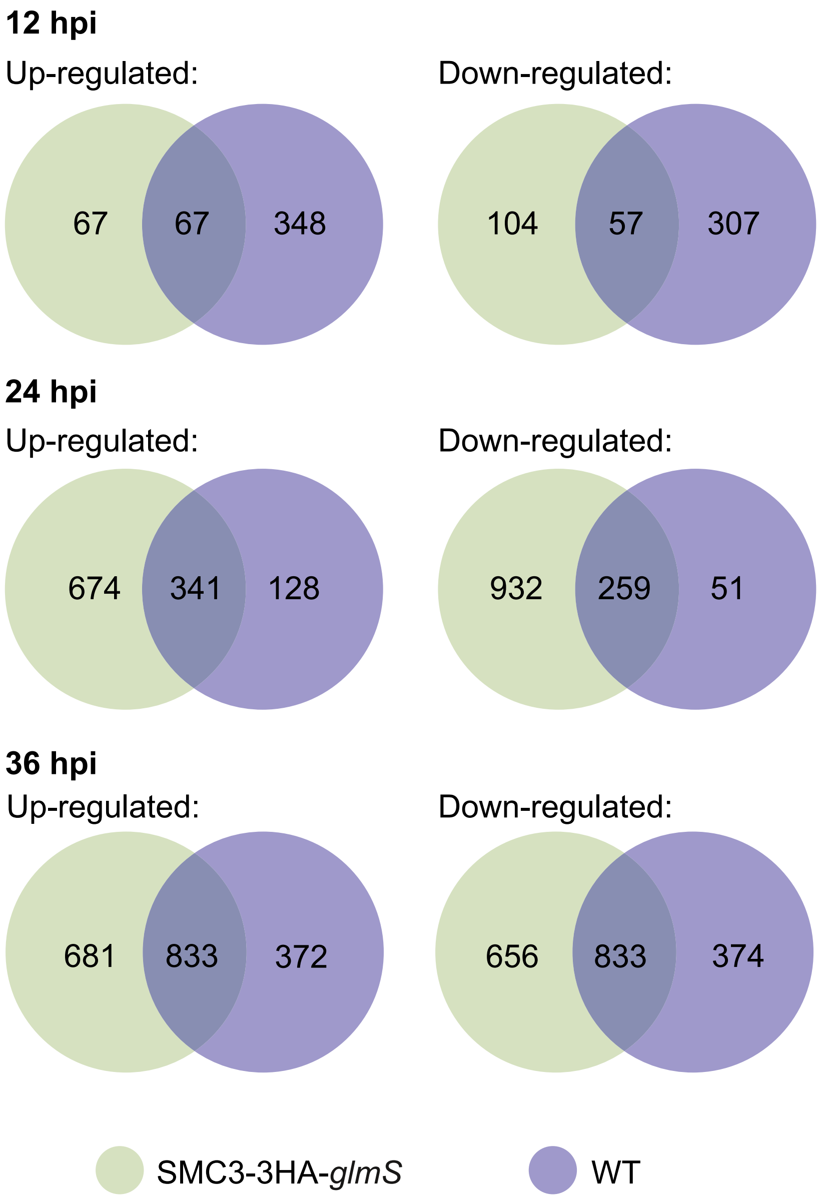

Figure EV4. Strategy for determining expression changes due to SMC3-3HA-glmS knockdown versus glucosamine treatment.

Venn diagram showing the number of unique or shared significantly up- or downregulated genes after two cycles of glucosamine treatment in synchronous, clonal populations of SMC3-3HA-glmS (green) and WT (purple) parasites at 12, 24, and 36 hpi.

 © 2023 The Authors

Figure EV5. SMC3 ChIP-seq analysis at invasion- or egress-related genes.

A Metagene plot showing average SMC3 enrichment (*y*-axis = ChIP/Input) in clonal SMC3-3HA-*glmS* parasites at 12 hpi from 1.5 kb upstream of the transcription start site (TSS) to 1.5 kb downstream of the transcription end site (TES) for genes that are significantly down- (gray) or upregulated (black) upon SMC3 knockdown. One replicate (clone B) was used for the SMC3 ChIP-seq. Dashed line represents a ChIP/Input ratio of 1.

B Metagene plot for a control ChIP-seq experiment (Data ref: Baumgarten *et al*, 2022a; Baumgarten *et al*, 2022b) showing average anti-HA enrichment (*y*-axis = ChIP/Input) in WT parasites at 12 hpi from 1.5 kb upstream of the transcription start site (TSS) to 1.5 kb downstream of the transcription end site (TES) for genes that are significantly down- (gray) or upregulated (black) upon SMC3 knockdown. One replicate was used for the anti-HA ChIP-seq. Dashed line represents a ChIP/Input ratio of 1.

C Metagene plot showing average SMC3 enrichment (*y*-axis = ChIP/Input) in clonal SMC3-3HA-*glmS* parasites at 12 (blue), 24 (coral), and 36 hpi (green) from 1.5 kb upstream of the transcription start site (TSS) to 1.5 kb downstream of the transcription end site (TES) for genes that are significantly upregulated upon SMC3 knockdown at 12 hpi. One replicate (clone B) was used for the SMC3 ChIP-seq. Dashed line represents a ChIP/Input ratio of 1.

D Metagene plot showing average SMC3 enrichment (*y*-axis = ChIP/Input) in clonal SMC3-3HA-*glmS* parasites at 12 (blue), 24 (coral), and 36 hpi (green) from 1.5 kb upstream of the transcription start site (TSS) to 1.5 kb downstream of the transcription end site (TES) for invasion-related genes, as defined in Hu *et al* (2010). One replicate (clone B) was used for the SMC3 ChIP-seq. Dashed line represents a ChIP/Input ratio of 1.

E Metagene plot showing average SMC3 enrichment (*y*-axis = ChIP/Input) in clonal SMC3-3HA-*glmS* parasites at 12 (blue), 24 (coral), and 36 hpi (green) from 1.5 kb upstream of the transcription start site (TSS) to 1.5 kb downstream of the transcription end site (TES) for randomly chosen genes that reach peak transcription in early-stage parasites. One replicate (clone A) was used for the SMC3 ChIP-seq. Dashed line represents a ChIP/Input ratio of 1.

F Metagene plot showing average SMC3 enrichment (*y*-axis = ChIP/Input) in clonal SMC3-3HA-*glmS* parasites at 12 (blue), 24 (coral), and 36 hpi (green) from 1.5 kb upstream of the transcription start site (TSS) to 1.5 kb downstream of the transcription end site (TES) for genes that reach peak expression in gametocytes. One replicate (clone A) was used for the SMC3 ChIP-seq. Dashed line represents a ChIP/Input ratio of 1.

G Metagene plot for a control ChIP-seq experiment (Data ref: Baumgarten *et al*, 2022a; Baugarten *et al*, 2022b) showing average anti-HA enrichment (*y*-axis = ChIP/Input) in WT parasites at 12 hpi from 1.5 kb upstream of the transcription start site (TSS) to 1.5 kb downstream of the transcription end site (TES) for invasion-related genes (black) as defined in Hu *et al* (2010), randomly chosen genes that reach peak transcription in early-stage parasites (gray), and genes that reach peak expression in gametocytes (gold). One replicate was used for the anti-HA ChIP-seq. Dashed line represents a ChIP/Input ratio of 1.

H ChIP-seq data showing enrichment of SMC3 (*y*-axis = ChIP/Input) at 12 (blue), 24 (coral), and 36 (green) hpi in clonal SMC3-3HA-*glmS* parasites at the *rhoptry-associated protein 2* (*rap2*, PF3D7_0501600) and the *glideosome-associated protein 45* (*gap45*, PF3D7_1222700) gene loci. The *x*-axis is DNA sequence, with the gene represented by a black box with white arrowheads to indicate transcription direction. One replicate (clone B) was used for ChIP-seq. ATAC-seq data from closely corresponding time points (15, 25, and 35 hpi) from Data ref: Toenhake *et al* (2018a); Toenhake *et al* (2018b) are shown in gray, with the *y*-axis representing ATAC-seq (RPM)/gDNA (RPM).

I ChIP-seq data showing enrichment of SMC3 (*y*-axis = ChIP/Input) at 12 (blue), 24 (coral), and 36 (green) hpi in clonal SMC3-3HA-*glmS* parasites at the *merozoite surface protein 1* (*msp1*, PF3D7_0930300) and the *merozoite surface protein 9* (*msp9*, PF3D7_1228600) gene loci. The *x*-axis is DNA sequence, with the gene represented by a black box with white arrowheads to indicate transcription direction. Two biological replicates (clones A and B) were used for ChIP-seq.

© 2023 The Authors

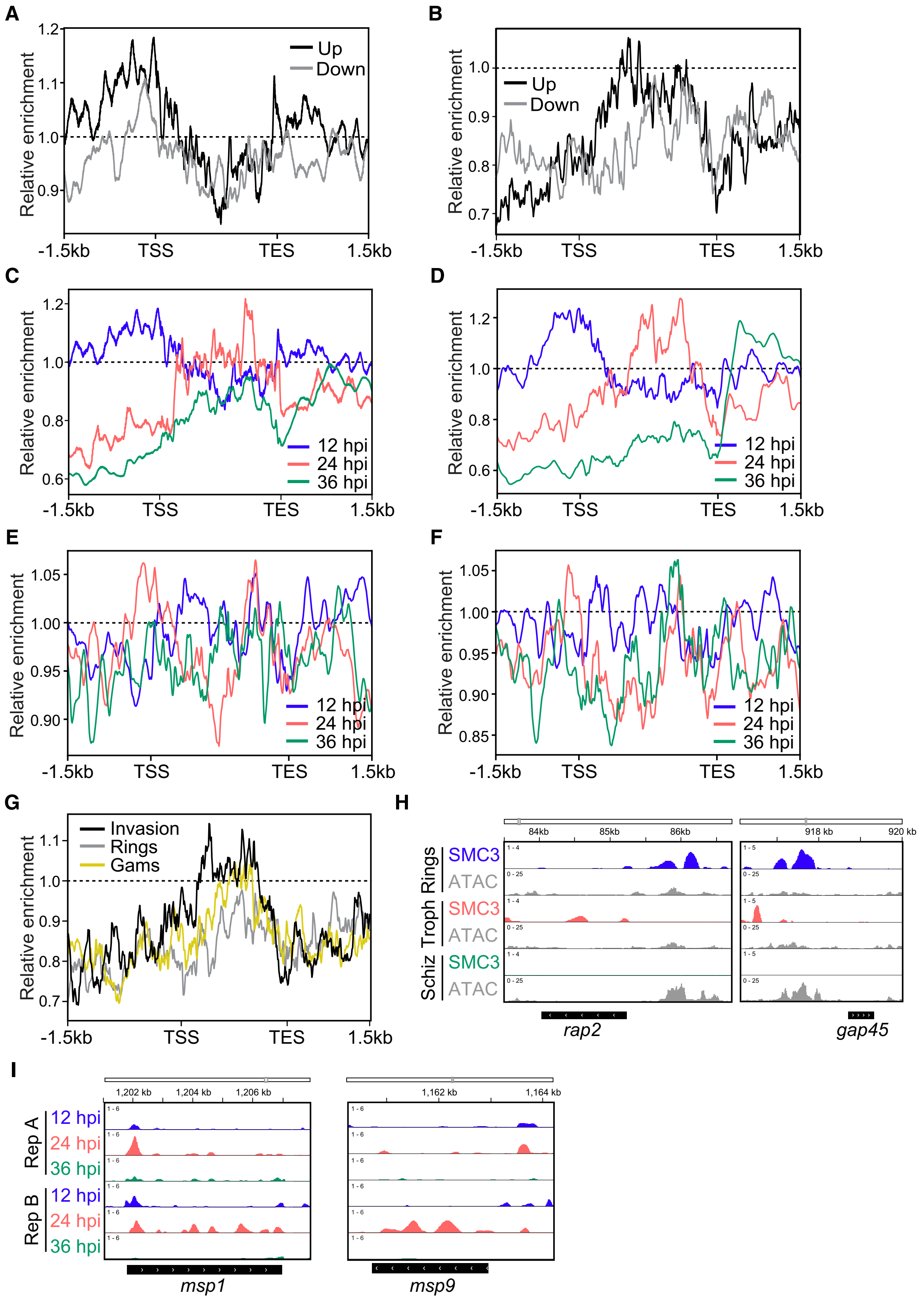

Figure EV5.

 © 2023 The Authors
