# Supplementary figures and images for: Cohesin contributes to transcriptional repression of stage‐specific genes in the human malaria parasite
Source: EMBO Rep. 2023 Aug 18;24(10):e57090. doi: 10.15252/embr.202357090 (PMC10561359; doi:10.15252/embr.202357090)

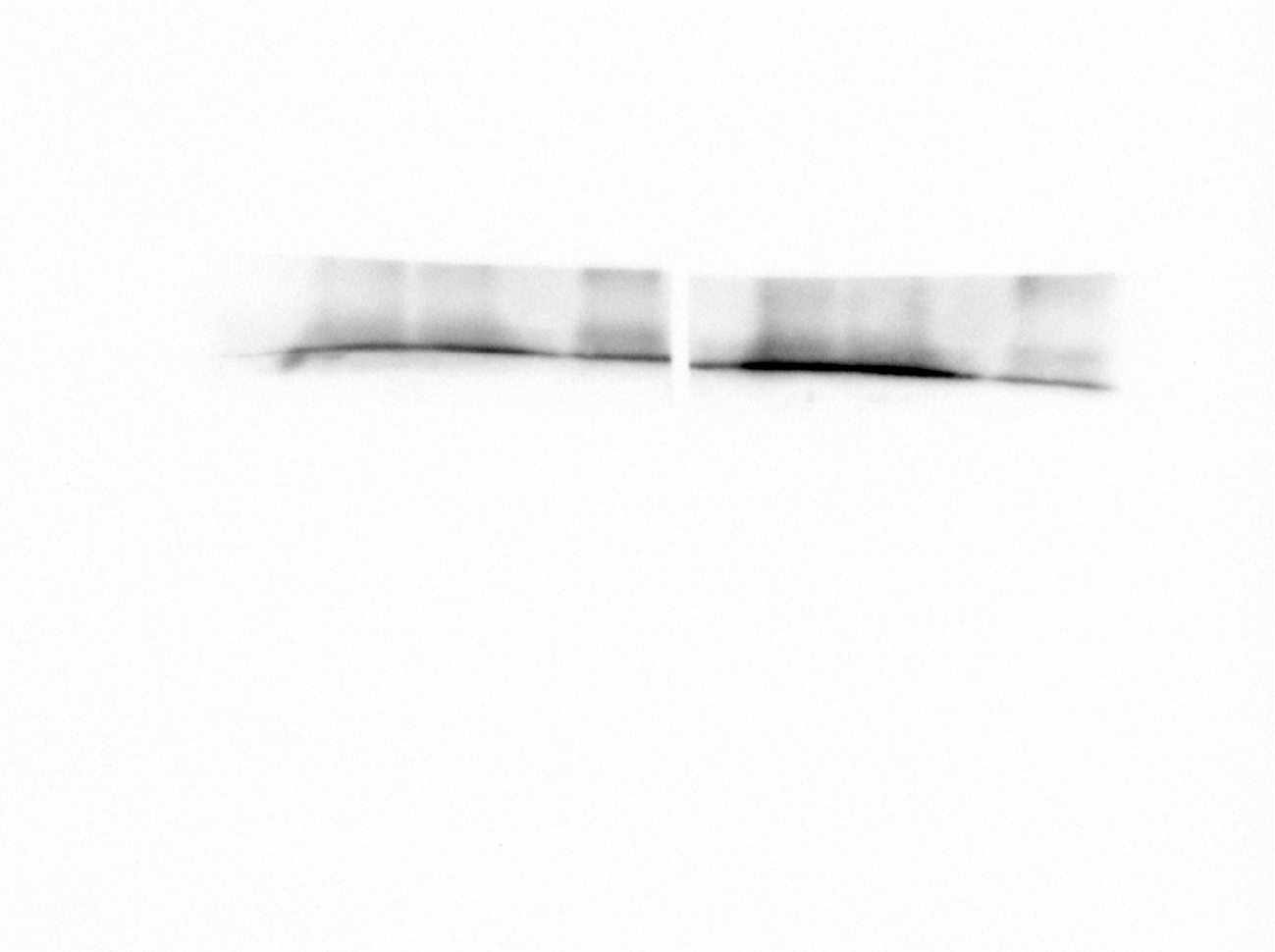

Supplement: Supplementary file 25 — Source Data for Figure 1 [file EMBR-24-e57090-s013.zip › Figure1/1D/EMBOR-2023-57090V2-Fig1D_antiH3_right-sd.tif]

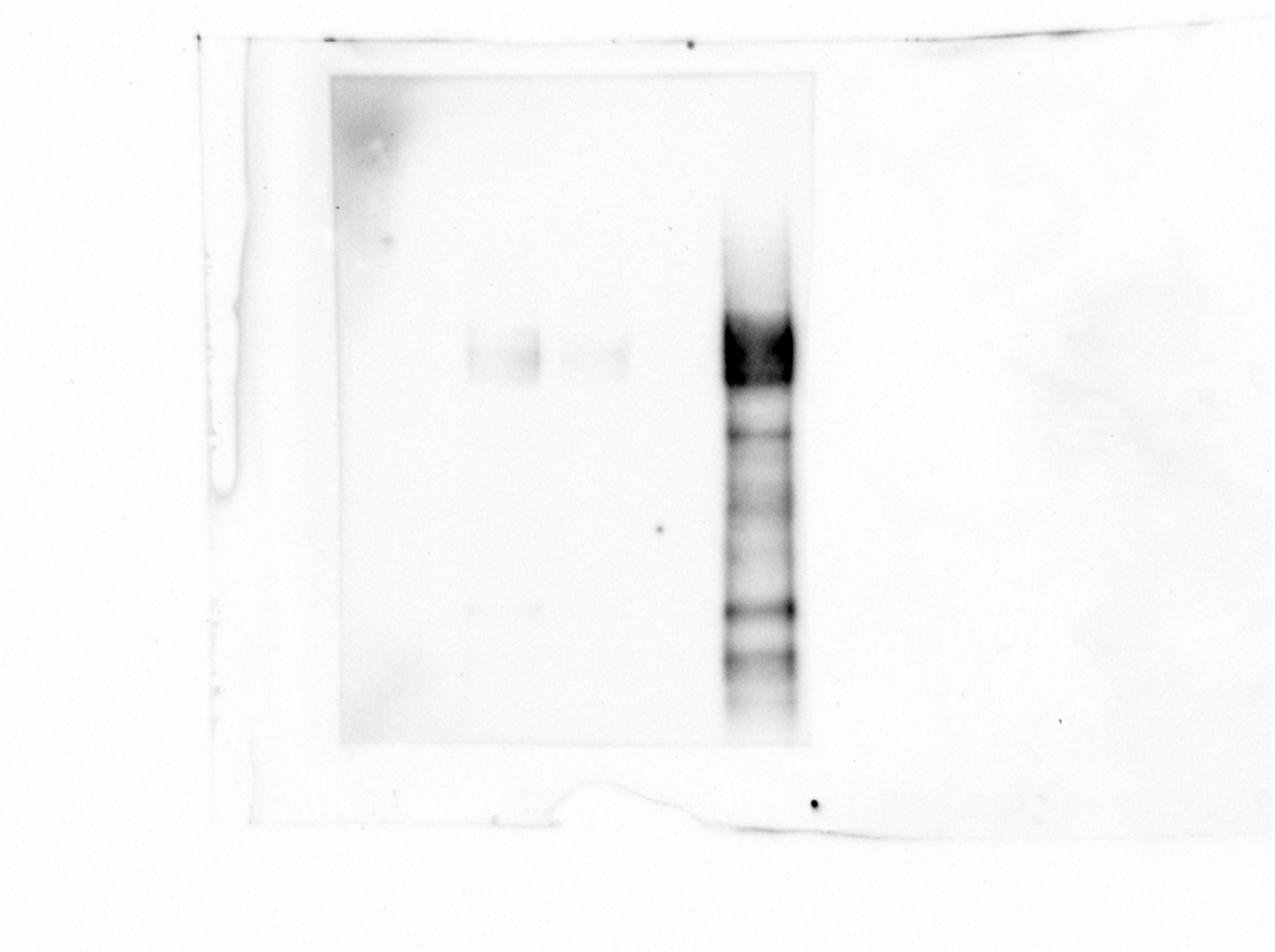

Supplement: Supplementary file 25 — Source Data for Figure 1 [file EMBR-24-e57090-s013.zip › Figure1/1D/EMBOR-2023-57090V2-Fig1D_antiHA-sd.tif]

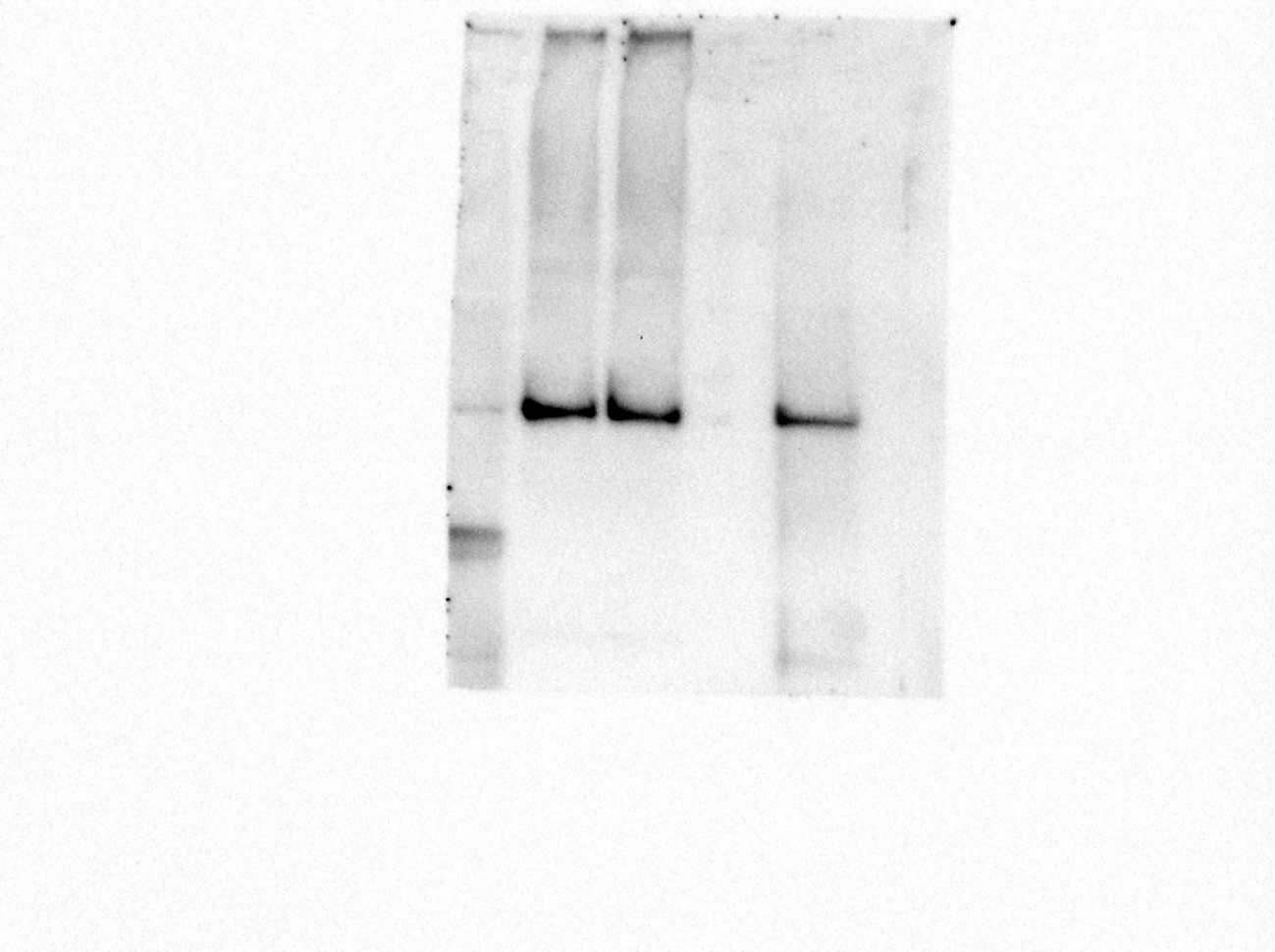

Supplement: Supplementary file 25 — Source Data for Figure 1 [file EMBR-24-e57090-s013.zip › Figure1/1D/EMBOR-2023-57090V2-Fig1D_antiSMC3-sd.tif]

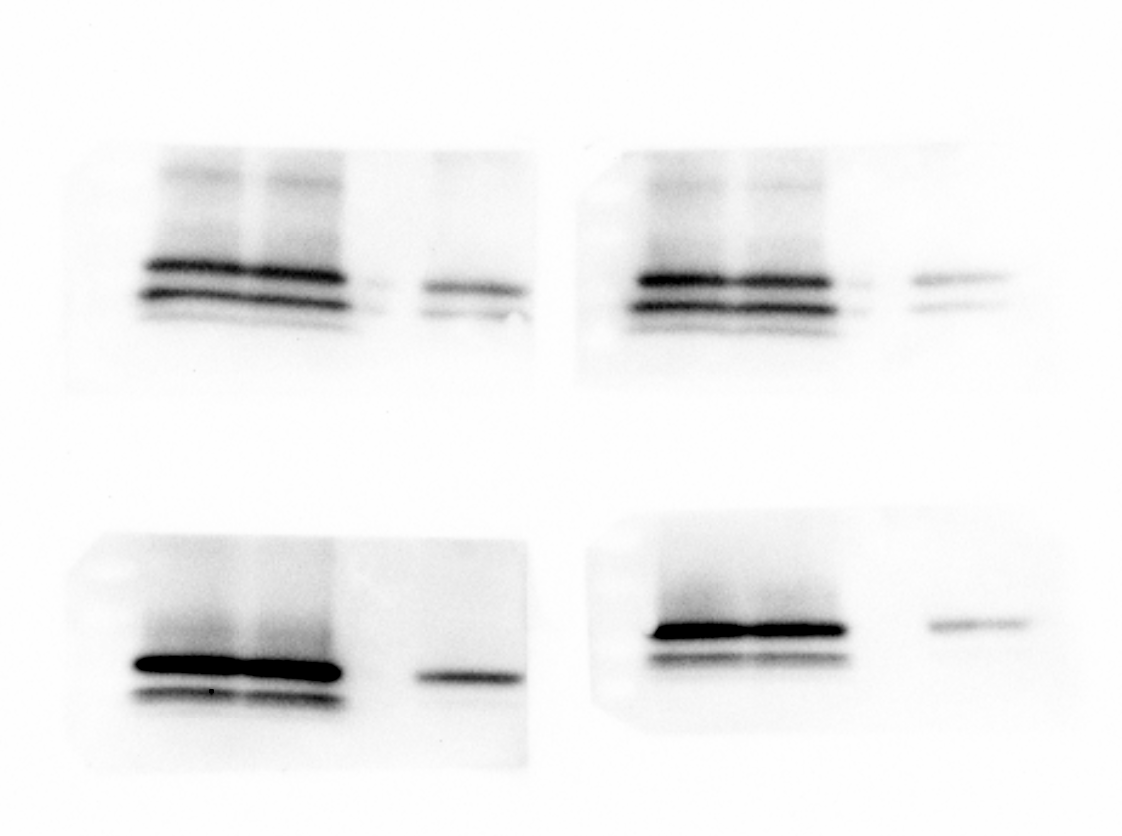

Supplement: Supplementary file 25 — Source Data for Figure 1 [file EMBR-24-e57090-s013.zip › Figure1/1E/EMBOR-2023-57090V2-Fig1E_antiH3_topright-sd.tif]

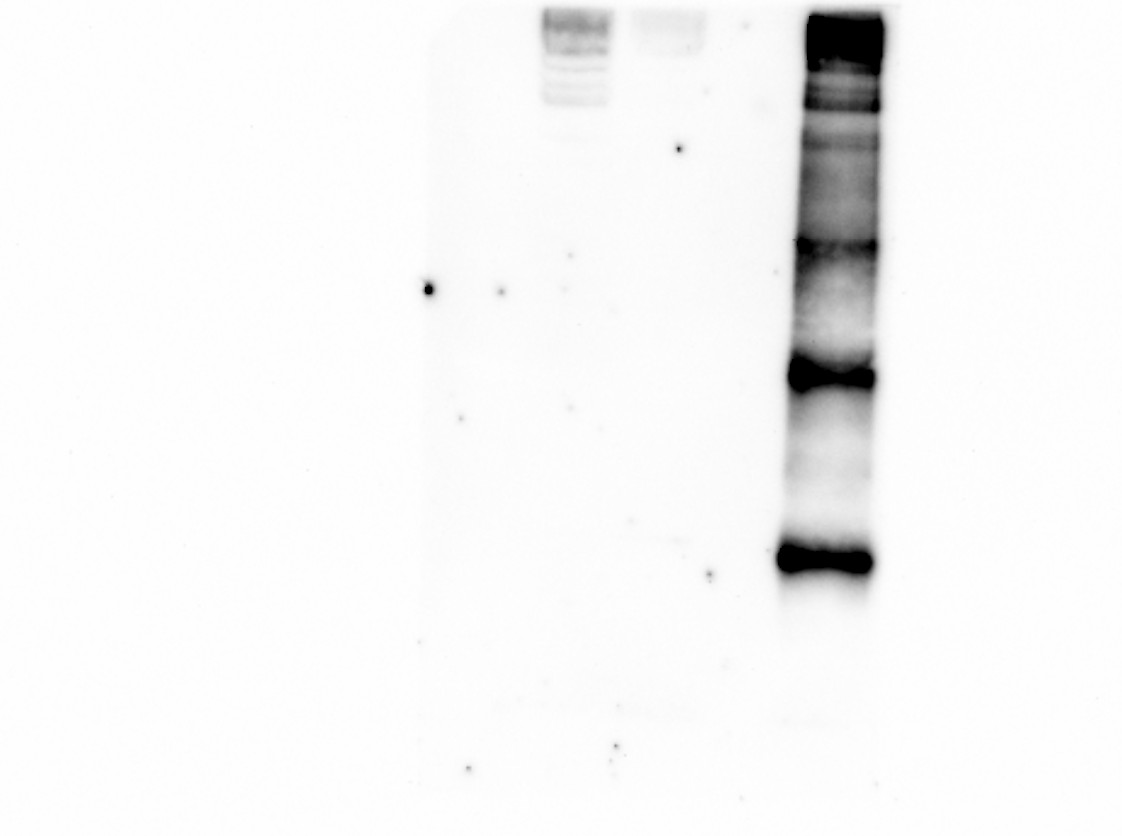

Supplement: Supplementary file 25 — Source Data for Figure 1 [file EMBR-24-e57090-s013.zip › Figure1/1E/EMBOR-2023-57090V2-Fig1E_antiHA-sd.tif]

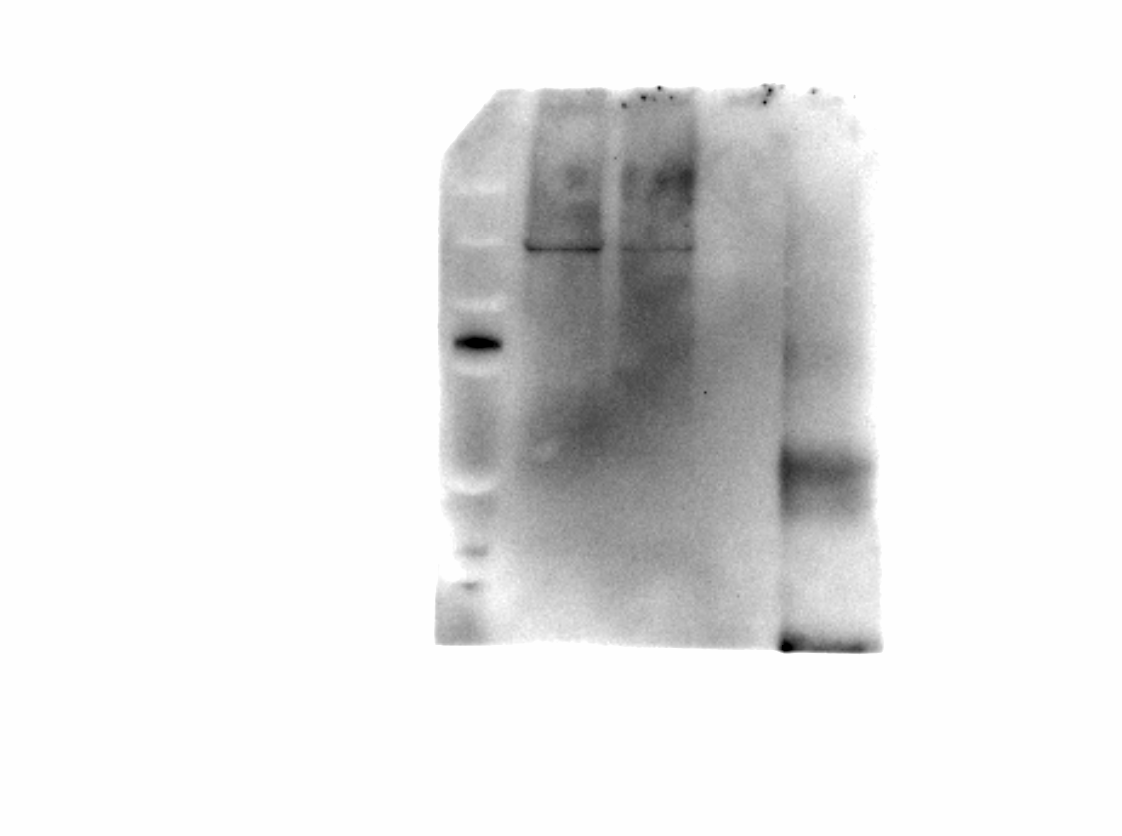

Supplement: Supplementary file 25 — Source Data for Figure 1 [file EMBR-24-e57090-s013.zip › Figure1/1E/EMBOR-2023-57090V2-Fig1E_IPIgG_antiSMC3-sd.tif]

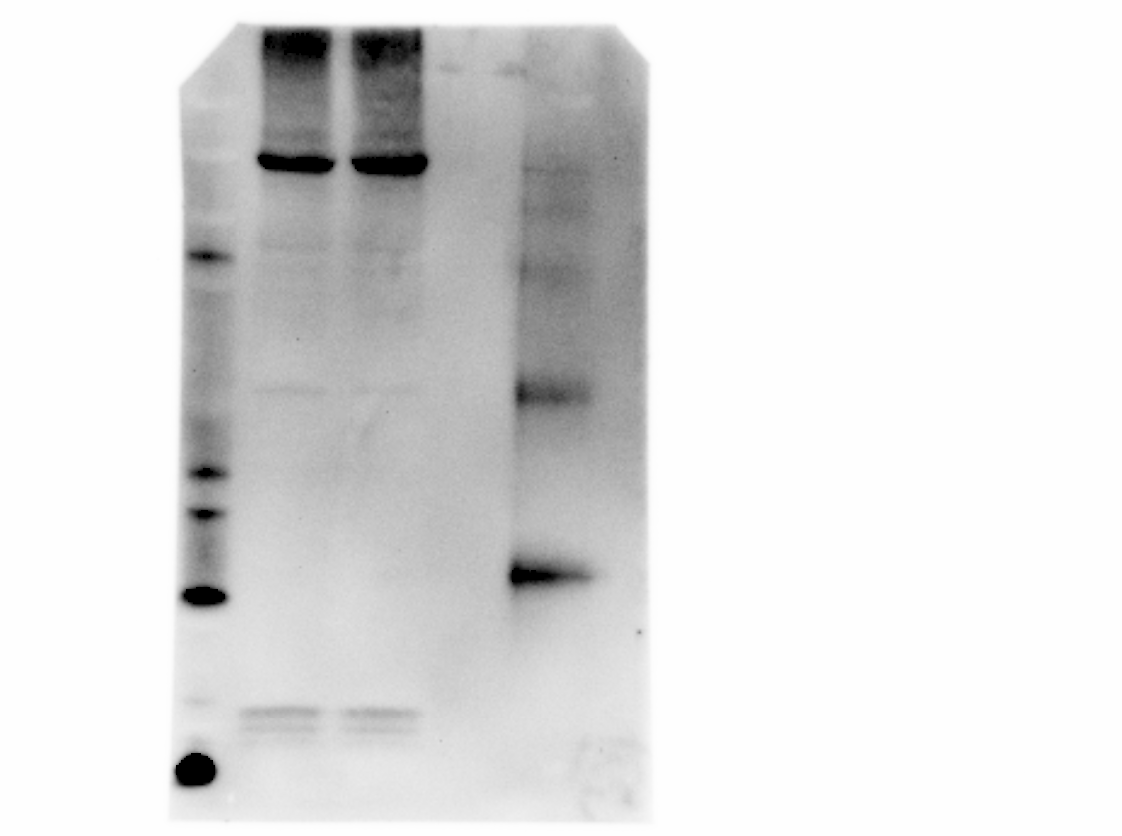

Supplement: Supplementary file 25 — Source Data for Figure 1 [file EMBR-24-e57090-s013.zip › Figure1/1E/EMBOR-2023-57090V2-Fig1E_IPSTAG_antiSMC3-sd.tif]

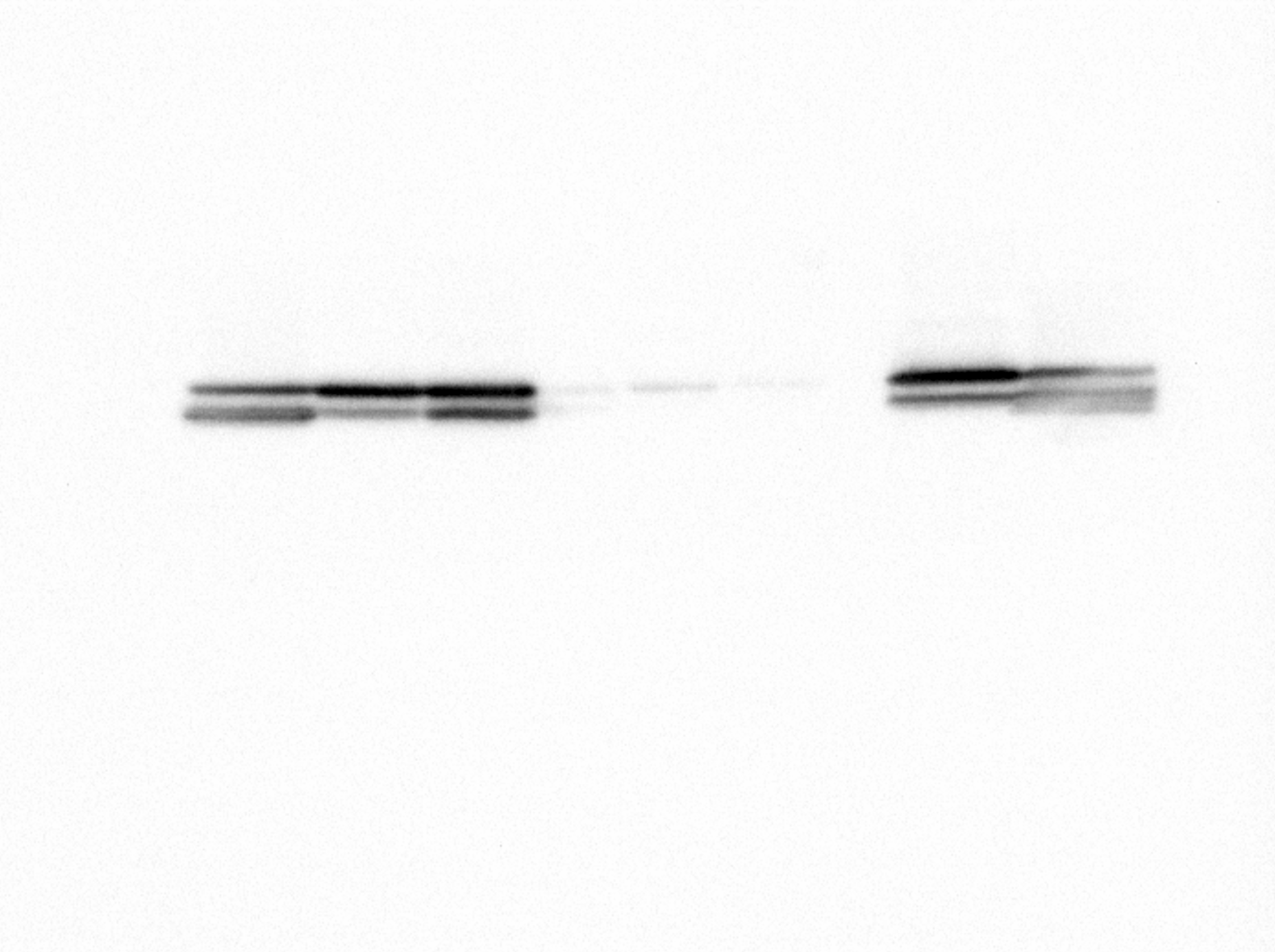

Supplement: Supplementary file 26 — Source Data for Figure 2 [file EMBR-24-e57090-s019.zip › Figure2/2A/EMBOR-2023-57090V2-Fig2A_antiH3-sd.tif]

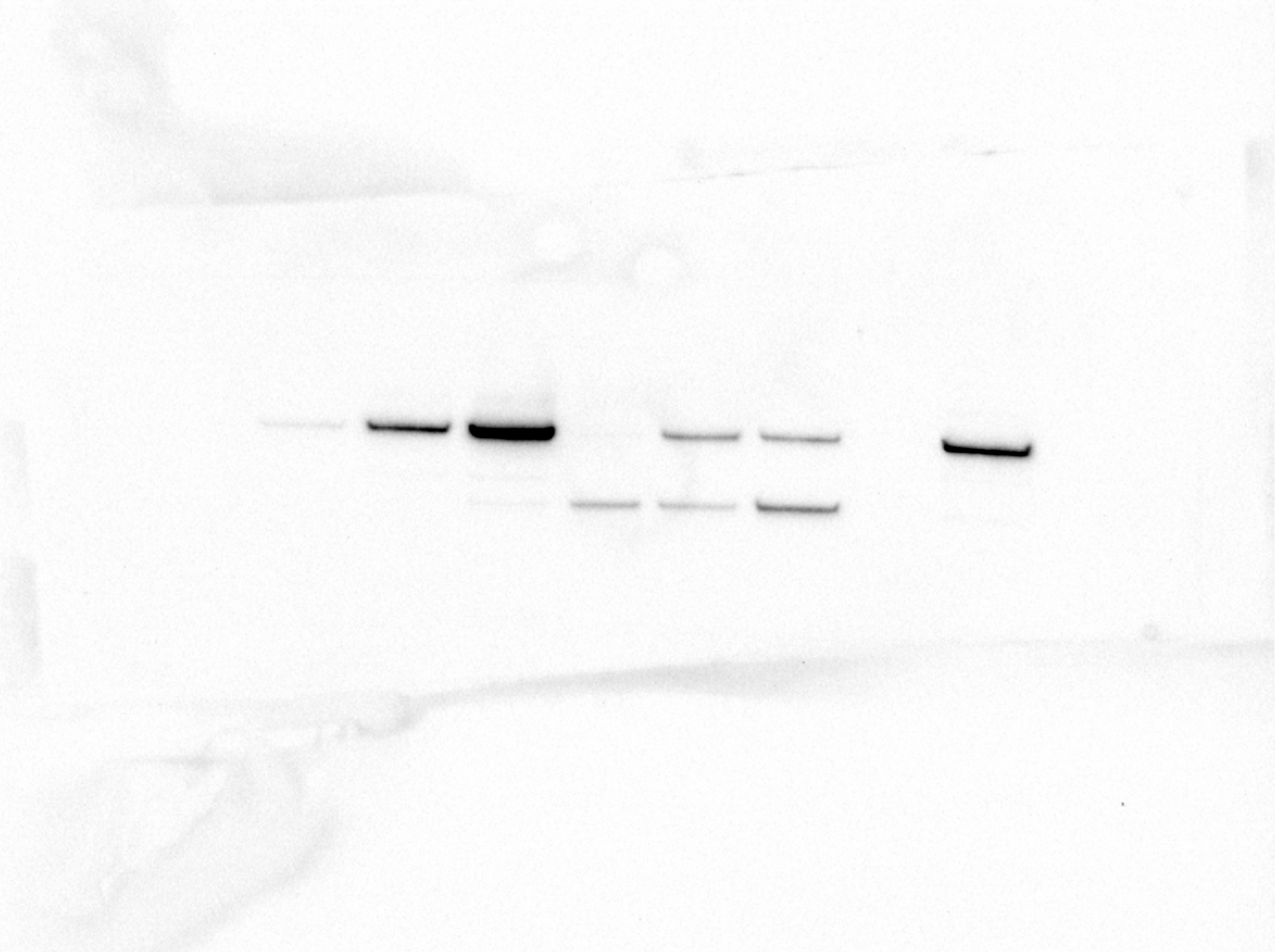

Supplement: Supplementary file 26 — Source Data for Figure 2 [file EMBR-24-e57090-s019.zip › Figure2/2A/EMBOR-2023-57090V2-Fig2A_antiHA-sd.tif]

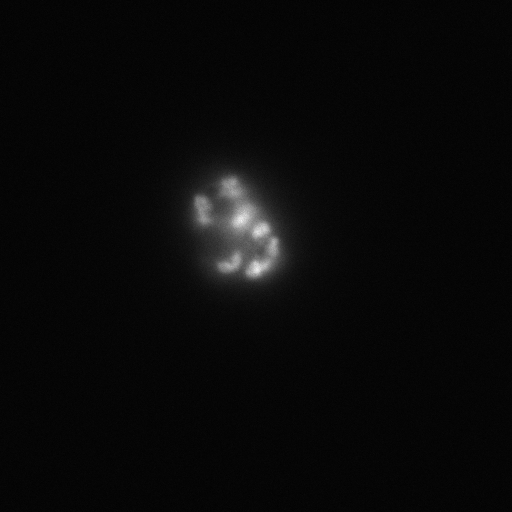

Supplement: Supplementary file 26 — Source Data for Figure 2 [file EMBR-24-e57090-s019.zip › Figure2/2B/2023-57090V2_SourceData_Fig2B_schizont_bottom_DAPI.tif]

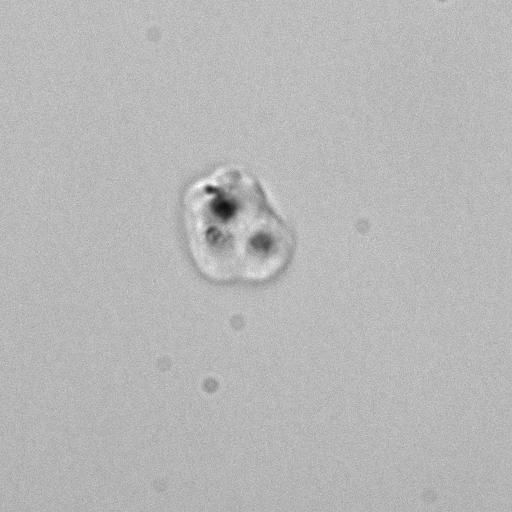

Supplement: Supplementary file 26 — Source Data for Figure 2 [file EMBR-24-e57090-s019.zip › Figure2/2B/2023-57090V2_SourceData_Fig2B_schizont_bottom_DIC.tif]

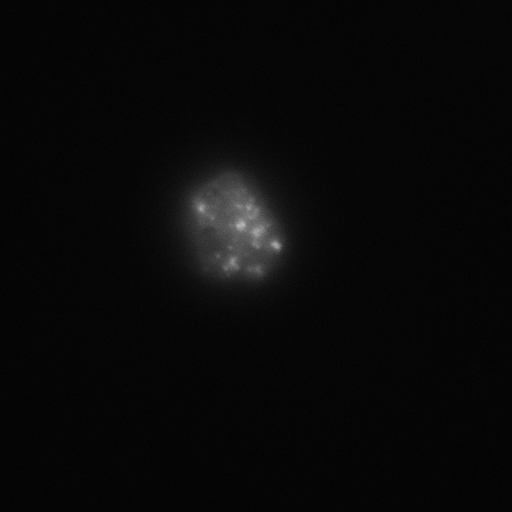

Supplement: Supplementary file 26 — Source Data for Figure 2 [file EMBR-24-e57090-s019.zip › Figure2/2B/2023-57090V2_SourceData_Fig2B_schizont_bottom_SMC3_3HA.tif]

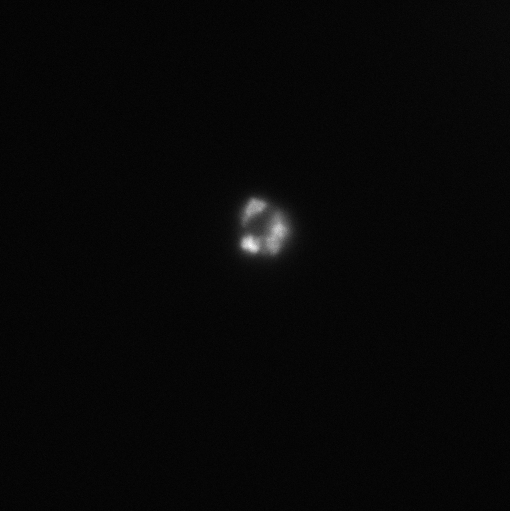

Supplement: Supplementary file 26 — Source Data for Figure 2 [file EMBR-24-e57090-s019.zip › Figure2/2B/2023-57090V2_SourceData_Fig2B_schizont_top_DAPI.tif]

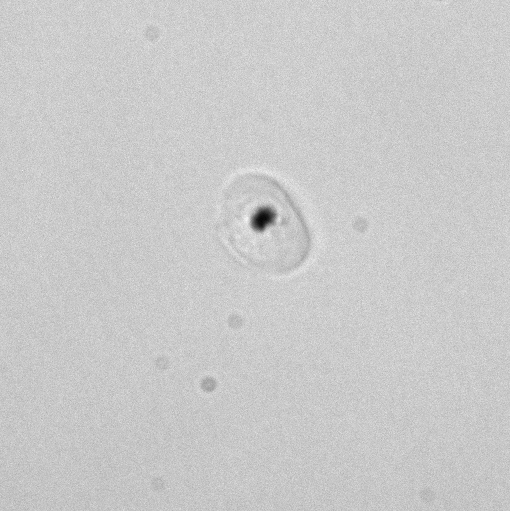

Supplement: Supplementary file 26 — Source Data for Figure 2 [file EMBR-24-e57090-s019.zip › Figure2/2B/2023-57090V2_SourceData_Fig2B_schizont_top_DIC.tif]

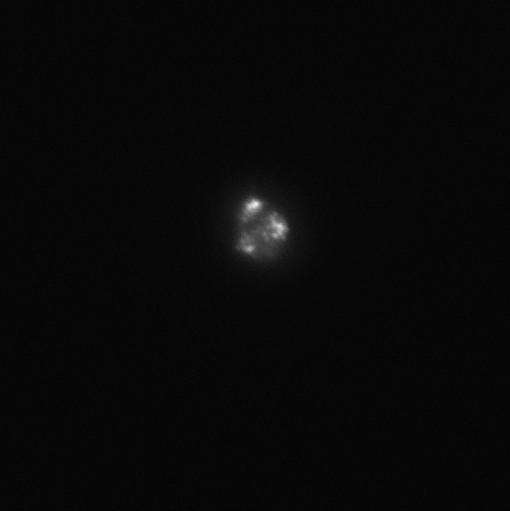

Supplement: Supplementary file 26 — Source Data for Figure 2 [file EMBR-24-e57090-s019.zip › Figure2/2B/2023-57090V2_SourceData_Fig2B_schizont_top_SMC3_3HA.tif]

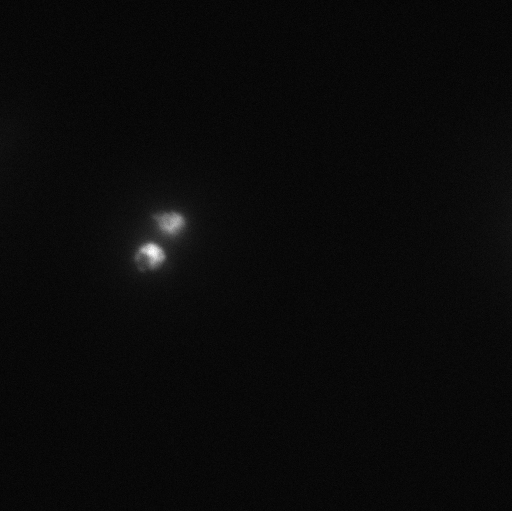

Supplement: Supplementary file 26 — Source Data for Figure 2 [file EMBR-24-e57090-s019.zip › Figure2/2B/2023-57090V2_SourceData_Fig2B_trophozoite_DAPI.tif]

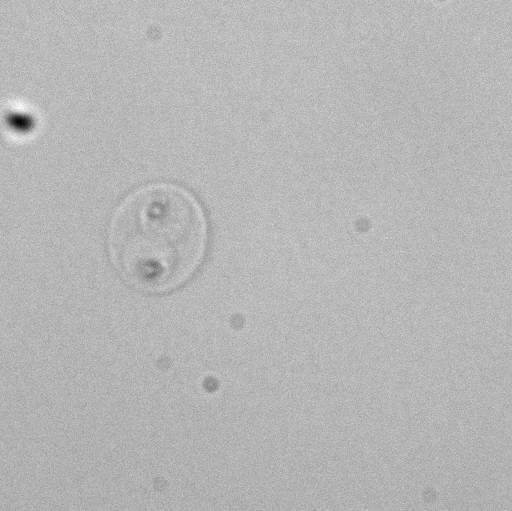

Supplement: Supplementary file 26 — Source Data for Figure 2 [file EMBR-24-e57090-s019.zip › Figure2/2B/2023-57090V2_SourceData_Fig2B_trophozoite_DIC.tif]

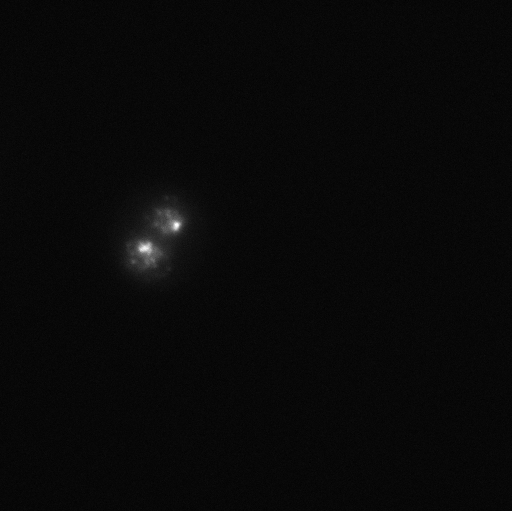

Supplement: Supplementary file 26 — Source Data for Figure 2 [file EMBR-24-e57090-s019.zip › Figure2/2B/2023-57090V2_SourceData_Fig2B_trophozoite_SMC3_3HA.tif]

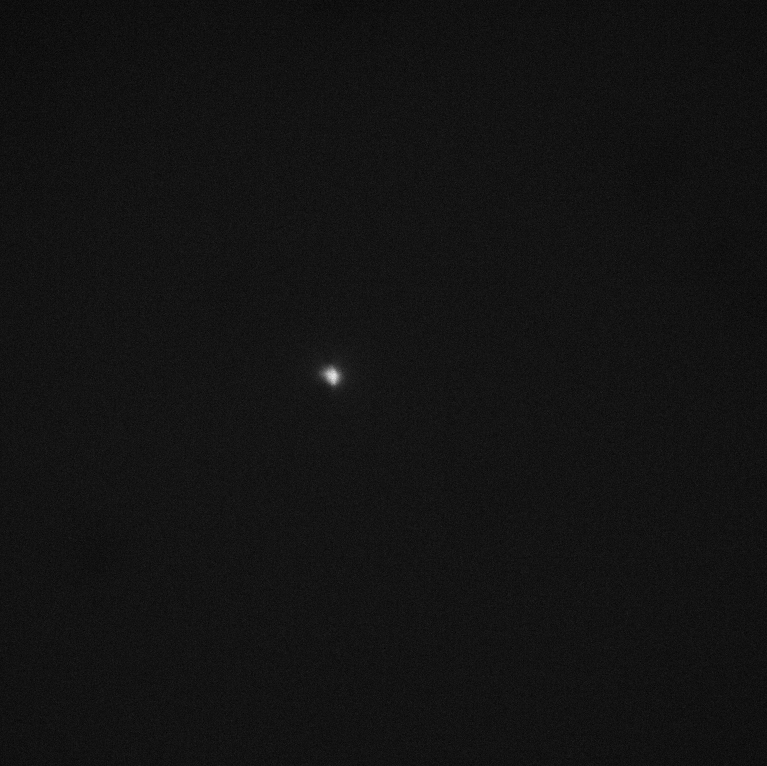

Supplement: Supplementary file 26 — Source Data for Figure 2 [file EMBR-24-e57090-s019.zip › Figure2/2C/EMBOR-2023-57090V2-Fig2C_DAPI-sd.tif]

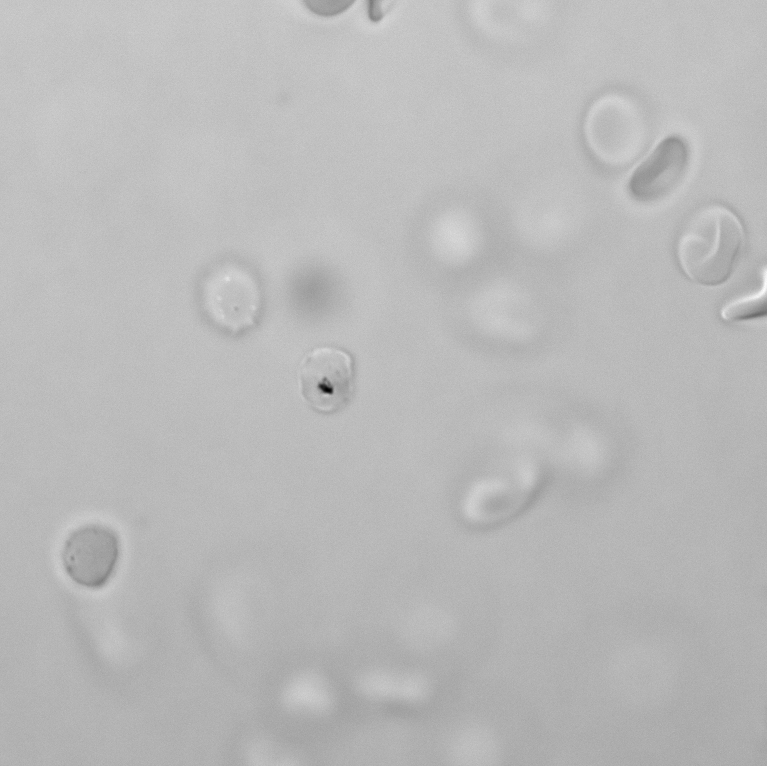

Supplement: Supplementary file 26 — Source Data for Figure 2 [file EMBR-24-e57090-s019.zip › Figure2/2C/EMBOR-2023-57090V2-Fig2C_DIC-sd.tif]

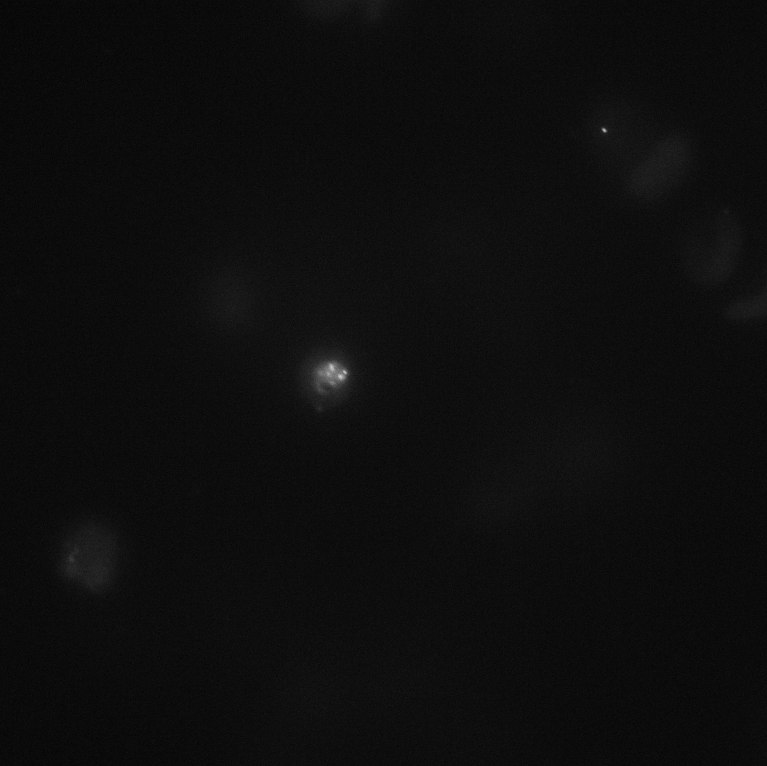

Supplement: Supplementary file 26 — Source Data for Figure 2 [file EMBR-24-e57090-s019.zip › Figure2/2C/EMBOR-2023-57090V2-Fig2C_HP1-sd.tif]

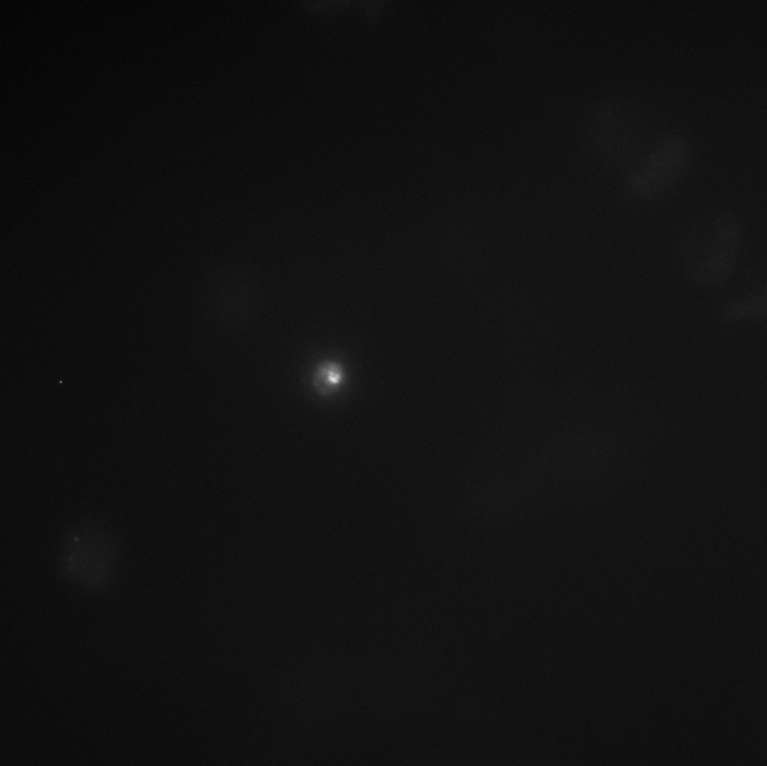

Supplement: Supplementary file 26 — Source Data for Figure 2 [file EMBR-24-e57090-s019.zip › Figure2/2C/EMBOR-2023-57090V2-Fig2C_SMC3_3HA-sd.tif]

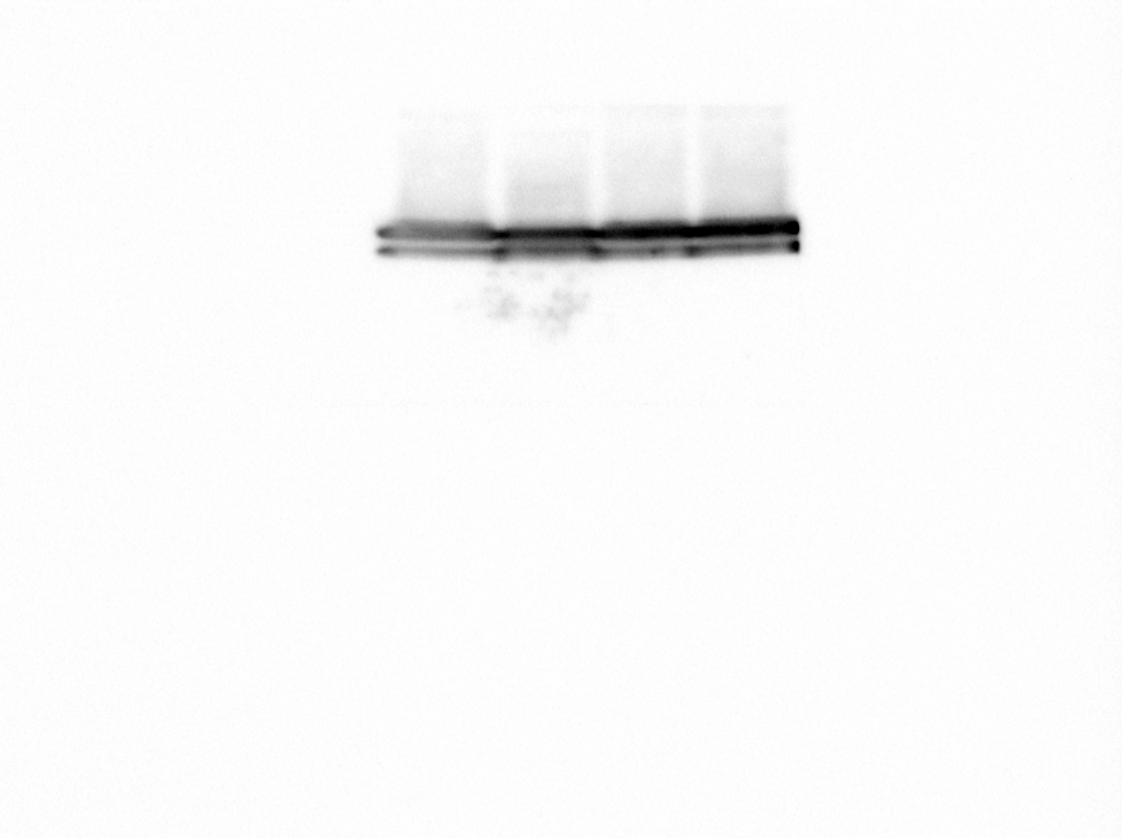

Supplement: Supplementary file 27 — Source Data for Figure 4 [file EMBR-24-e57090-s008.zip › Figure4/4A/EMBOR-2023-57090V2-Fig4A_12hpi_antiH3-sd.tif]

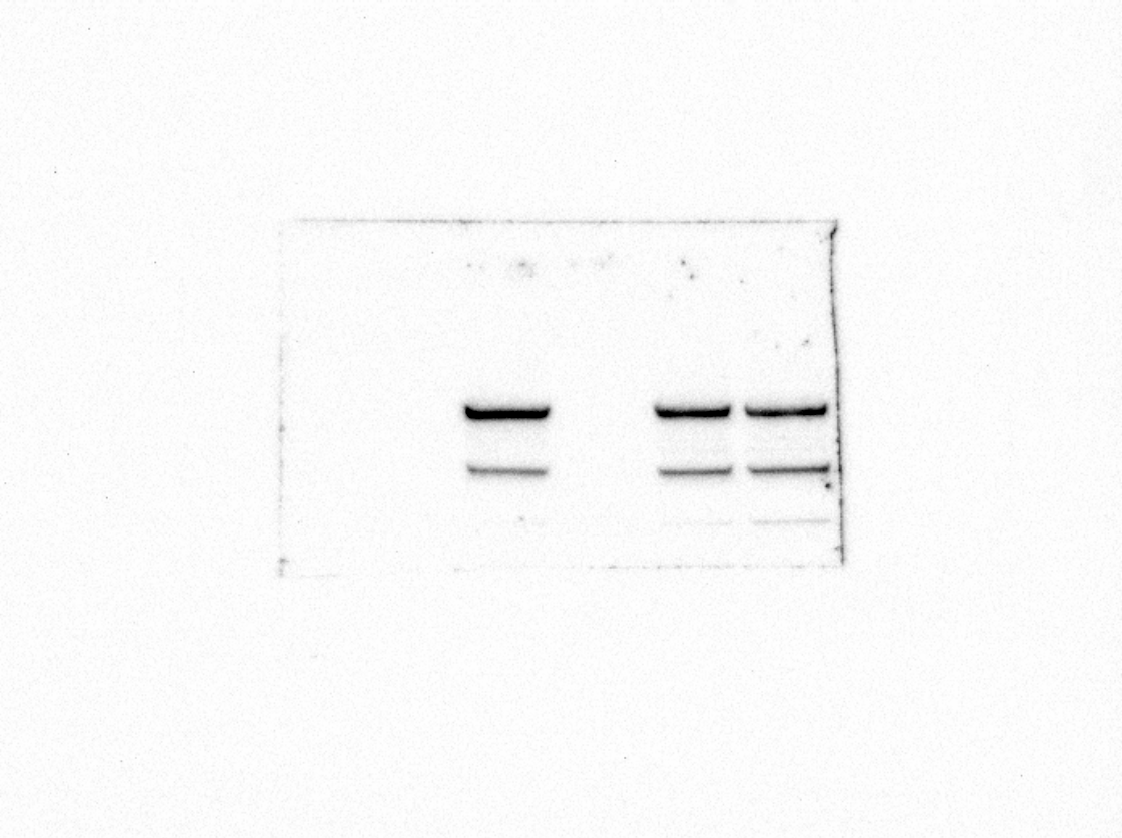

Supplement: Supplementary file 27 — Source Data for Figure 4 [file EMBR-24-e57090-s008.zip › Figure4/4A/EMBOR-2023-57090V2-Fig4A_12hpi_antiHA-sd.tif]

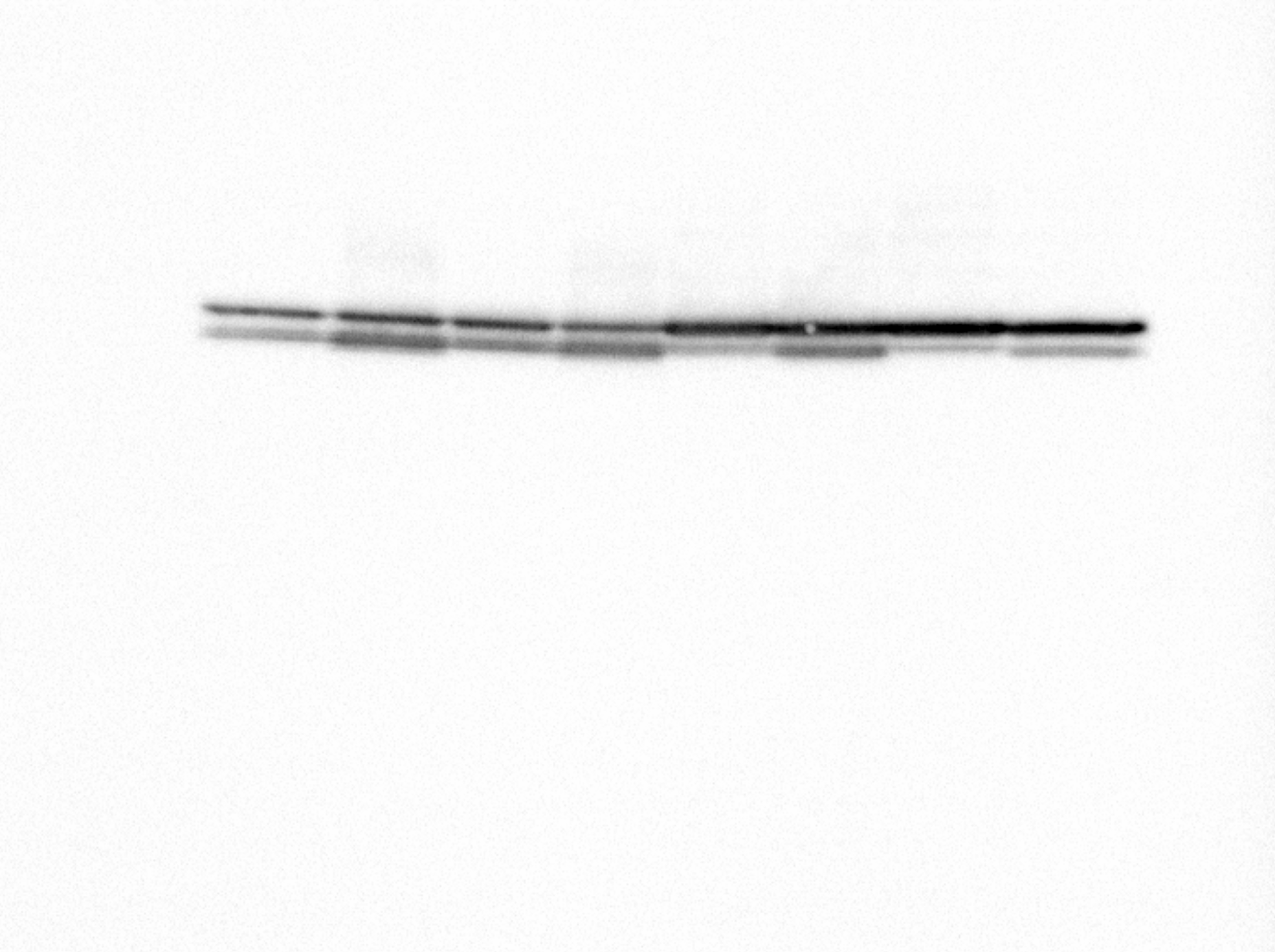

Supplement: Supplementary file 27 — Source Data for Figure 4 [file EMBR-24-e57090-s008.zip › Figure4/4A/EMBOR-2023-57090V2-Fig4A_24hpi_36hpi_antiH3-sd.tif]

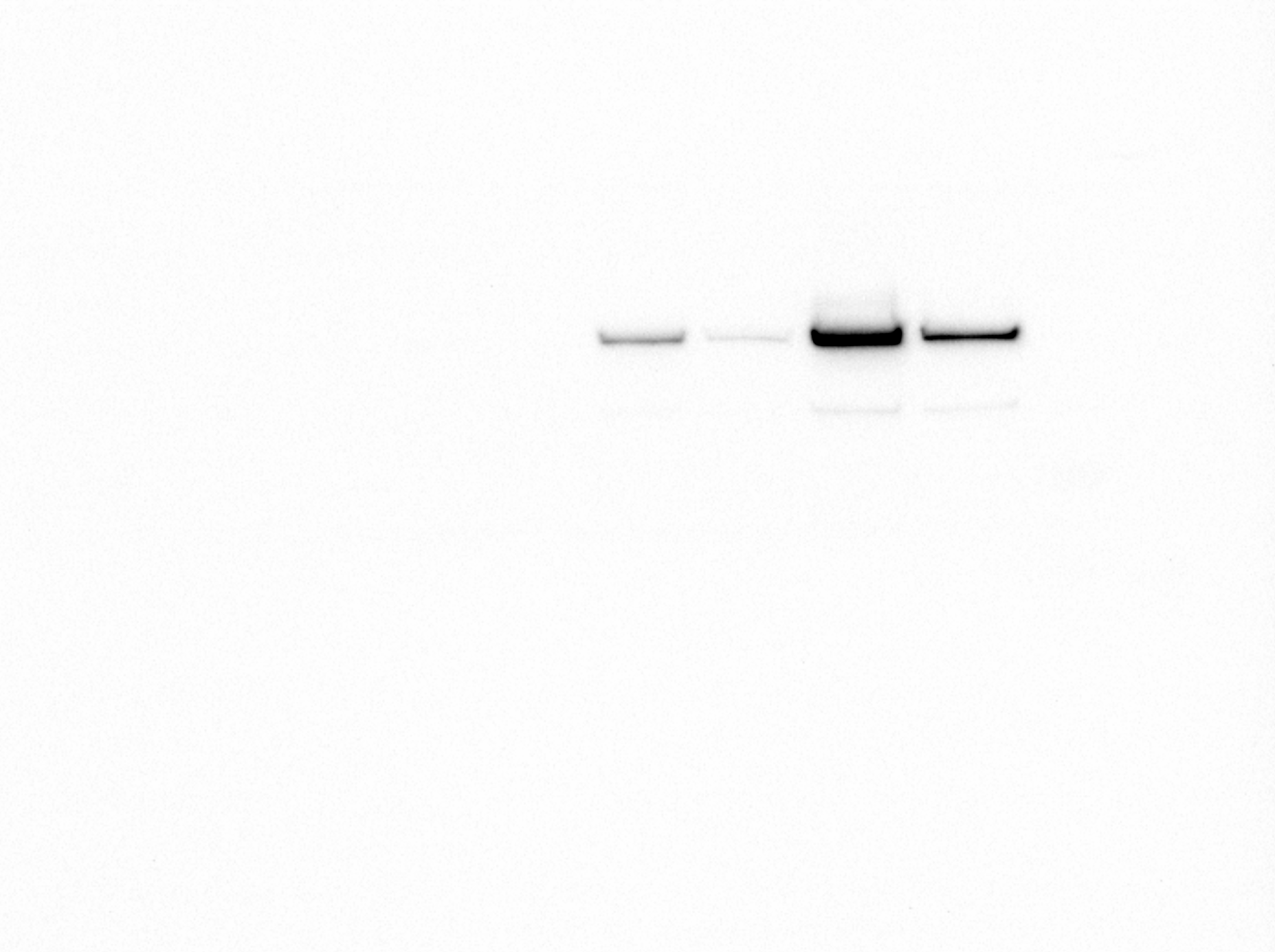

Supplement: Supplementary file 27 — Source Data for Figure 4 [file EMBR-24-e57090-s008.zip › Figure4/4A/EMBOR-2023-57090V2-Fig4A_24hpi_36hpi_antiHA-sd.tif]
