# Supplementary material for: Cohesin contributes to transcriptional repression of stage‐specific genes in the human malaria parasite
Source: EMBO Rep. 2023 Aug 18;24(10):e57090. doi: 10.15252/embr.202357090 (PMC10561359; doi:10.15252/embr.202357090)
Supplement: Supplementary file 28 [file EMBR-24-e57090-s012.pdf]

EMBO reports

Cohesin contributes to transcriptional repression of stage-specific genes in the human malaria parasite

Catarina Rosa, Parul Singh, Patty Chen, Ameya Sinha, Aurelie Claes, Peter Preiser, Peter Dedon, Sebastian Baumgarten, Artur Scherf, and Jessica Bryant
DOI: 10.15252/embr.202357090

Corresponding author(s): Jessica Bryant (jessica.bryant@pasteur.fr)

Review Timeline:

**Transfer from Review Commons:**	28th Feb 23
**Editorial Decision:**	20th Apr 23
**Revision Received:**	12th Jun 23
**Editorial Decision:**	12th Jul 23
**Revision Received:**	18th Jul 23
**Accepted:**	21st Jul 23

Editor: Esther Schnapp

Transaction Report: This manuscript was transferred to EMBO reports following peer review at Review Commons.

(Note: With the exception of the correction of typographical or spelling errors that could be a source of ambiguity, letters and reports are not edited. Depending on transfer agreements, referee reports obtained elsewhere may or may not be included in this compilation. Referee reports are anonymous unless the Referee chooses to sign their reports.)

Review #1

1. Evidence, reproducibility and clarity:

Evidence, reproducibility and clarity (Required)

Summary

The present work by Rosa et al., provides convincing data about the presence and functional relevance of the cohesin complex in Plasmodium falciparum blood stages. In accordance with other organisms, the composition of the cohesin complex containing SMC1, SMC3 RAD21 and putatively STAG could be confirmed via pulldown and mass spectrometry. Basic characterization of endogenous tagged SMC3 demonstrated the expression and nuclear localization during IDC, as well as the relatively stable accumulation at centromeric regions, consistent with the known cohesin function in chromatid separation. Furthermore, dynamic and stage-dependent binding to intergenic regions observed in ChIPseq and major transcriptome aberrations upon knockdown of SMC3 (<70% mRNA reduction via glmS-system) suggest an important function in transcriptional regulation. The underlying mechanism of gene regulation by cohesin via the creation of chromatin clusters or DNA loops as well as its specificity to target sites remain speculation.

Major criticism

- Accuracy of ChIPseq with only one replicate per time point (and lacking negative controls without antibody) is not convincing. The authors should include more replicates.
- Proposed mechanism of repressive effect of SMC3 early in IDC on genes, that get de-repressed in late stages: To claim this mode of function, it would be necessary to include a KD on late stage parasites. If there is an early repressive role of SMC3, upregulated genes should not be affected by late SMC3-KD. Furthermore, the hypothesized repressive effect of SMC3 does not explain the numerous genes downregulated in KD.
- Due to the fact, that the KD was induced at the exact same timepoint and analysed 12h and 24h after induction it is possible that identified, differentially expressed genes at 24h are not directly regulated by SMC3, but rather due to a general deregulation of gene expression. Did the authors attempt to analyse gene expression upon induction at ring, trophozoite and schizont stage?
- Based on rapid parasite growth, the authors hypothesize a higher invasion rate due to upregulation of invasion genes. This hypothesis is not supported by quantitative invasion assays or quantification of invasion factors on the protein level. An alternative explanation could be a shorter cell cycle (<48h), as the different cell cycle progression estimation of KD/WT could indicate (SuppFigure 3). Giemsa-stain images of KD/WT parasites should be included to show normal stage development over time.
- Correlation of SMC3-occupancy/ATAC/expression profile of the exemplary genes rap2 and gap45 (Figure 4C,D,E): is this representative for all upregulated genes?
- Given that SMC3 appears to be not essential for parasite growth, the authors could generate a null mutant for SMC3, which might allow for easier analysis of differences in gene regulation, cell cycle progression and/or invasion efficiency.

2. Significance:

Significance (Required)

Own opinion

The authors provide a basic characterization of the cohesin component SMC3 using NGS methods to investigate chromatin binding sites and its potential influence on gene expression. The localisation of SMC3 at centromers as described previously (Batugedara 2020) was confirmed. However, the dynamic binding to other regions in the genome, potentially mediated by other proteins, could not be resolved unequivocal with only one replicate of ChIPseq per time point.
Similarly, the RNAseq data demonstrate the relevance of SMC3 for gene expression, but no clear picture of a regulatory mechanism can be drawn at his point. Lacking information about the mode of binding as well as the setup of transcriptome analysis (only two time-shifted sampling points after simultaneous glmS treatment for 96h resulting in incomplete knockdown) cannot definitely elucidate, if SMC3/cohesin is a chromatin factor that affects transcription of genes in general or a specific repressor of stage-specific genes.

The work will be interesting to a general audience, interested in gene regulation and chromatin remodelling

The reviewers are experts in Plasmodium cell biology and epigenetic regulation.

3. How much time do you estimate the authors will need to complete the suggested revisions:

Estimated time to Complete Revisions (Required)

(Decision Recommendation)

Between 3 and 6 months

4. *Review Commons* values the work of reviewers and encourages them to get credit for their work. Select 'Yes' below to register your reviewing activity at Web of Science Reviewer Recognition Service (formerly Publons); note that the content of your review will not be visible on Web of Science.

Web of Science Reviewer Recognition

No

Review #2

1. Evidence, reproducibility and clarity:

Evidence, reproducibility and clarity (Required)

Rosa et al, Review Commons

The manuscript by Rosa et al. addresses the function of the cohesion subunit Smc3 in gene regulation during the asexual life cycle of P. falciparum. Cohesin is a conserved protein complex involved in sister chromatin cohesion during mitosis and meiosis in eukaryotic cells. Cohesin also modulates transcription and DNA repair by mediating long range DNA interactions and regulating higher order chromatin structure in mammals and yeast. In P. falciparum, the Cohesin complex remains largely uncharacterized. In this manuscript, the authors present mass spectrometry data from co-IPs showing that Smc3 interacts with Smc1 and a putative Rad21 orthologue (Pf3D7_1440100, consistent with published data from Batugedara et al and Hilliers et al), as well as a putative STAG domain protein orthologue (PF3D7_1456500). Smc3 protein appears to be most abundant in schizonts, but ChIPseq indicates predominant enrichment of Smc3 in centromers in ring and trophozoite stages. In addition, Smc3 dynamically binds with low abundance to other loci across the genome; however, the enrichment is rather marginal and only a single replicate was conducted for each time point making the data interpretation difficult. Conditional knock-down using a GlmS ribozyme approach indicates that parasites with reduced levels of Smc3 have a mild growth advantage, which is only evident after five asexual replication cycles and which the authors attribute to the transcriptional upregulation of invasion-linked genes following Smc3 KD. Indeed, Smc3 seems to be more enriched upstream of genes that are upregulated after Smc3 KD in rings than in downregulated genes, indicating that Smc3/cohesin may have a function in supressing transcription of these schizont specific genes until they are needed. The manuscript is concise and very well written, however it suffers from the lack of experimental replicates for ChIP experiments and a better characterization of the phenotype of conditional KD parasites.

Major comments

- In the mass spectrometry analysis, many seemingly irrelevant proteins are identified at similar abundance to the putative rad21 and ssc3 orthologues, and therefore the association with the cohesion complex seems to be based mostly on analogy to other species rather than statistical significance. Hence, it would be really nice to see a validation of the novel STAG domain and Rad21 proteins, for example by Co-IP using double transgenic parasites.
- The ChIPseq analysis presented here is based on single replicates for each of the three time points. The significance cutoffs for the peaks are rather high ($q < 0.05$). Therefore, the relevance of the marginally enriched dynamic peaks (average relative enrichment of

<1.2 fold for genes upregulated in rings 12 hpi in Figure 4A and B) does not appear to be very robust. Even in ChIPseq experiments using non-immune IgG, hundreds of peaks are usually called with MACS2 with a similar magnitude. So, to substantiate the data for extra-centromeric peaks convincingly, replicates and more stringent statistics are necessary. In addition, the authors should also compare their data to published PfSmc3 ChIP data from Batugedara et al 2020 (GSE116219).
- The authors argue that during schizogony, cohesin may no longer be required at centromers, explaining the low ChIPsignal at this stage (Line 301). However, during schizogony parasites undergo repeated rounds of DNA replication (S-phase) and mitosis (M-phase) to generate multinucleated parasites; and concentrated spots of Smc3 are observed in each nucleus in schizonts by IFA. In turn, the strong presence of Smc3 at centromers in ring stage parasites is surprising, particularly since the Western Blot in Figure 1D shows most expression of Smc3 in schizonts and least in rings; and Smc3 is undetectable in rings by IFA. Yet, the ChIP signal shows very strong enrichment at centromers, long before S phase produces sister chromatids. What could be the reason for this discrepancy? Again, ChIP replicates and controls would be helpful in distinguishing technical problems with the ChIP from biologically relevant differences.
- It is surprising that a conserved protein like Smc3 shows such a subtle phenotype, given that it is predicted to be essential and its orthologues have a function in mitosis. Generally, only limited data are presented to characterize the Smc3 KD parasites, and more detail should be included. For example validation of the parasite line using a PCR screen for integration and absence of wt, parasite morphology after KD, and/or analysis of the KD parasites for cell cycle status.
- Synchronization was performed at the beginning of the growth time course, which would be expected to result in a stepwise increase in parasitemia every 48 hours; however, the parasitemia according to Fig. 4F rises steadily, which would indicate that the parasites are actually not very synchronous.
- The question of whether Smc3 causes a shorter parasite life cycle (quicker progression) or more invasion is important and could be experimentally addressed by purifying synchronous schizont stage parasites and determining their invasion rates as well as morphological examination of the Giemsa smears over the time course.
- Please also compare Smc3 transcriptional levels in transgenic parasites to those in wt parasites to rule out that the genetic modification has lead to artificial upregulation of Smc3 transcription.
- According to Figure S2, even more genes were deregulated at the 12 hpi time point in the WT parasites than in Smc3 parasites, and even to a much higher extent. What "transcriptional age" did the WT control parasites have at each time point?
- A negative correlation with transcription is well established in S. cerevisiae, particularly at inducible genes. How does Smc3 enrichment generally look like for genes that show maximal expression at each of the time point?
- Line 590: according to the methods, a 36 hpi KD time point was also harvested. Why are the data not shown/analysed?

Minor Comments

- Line 103/104: the hinge domain and ATPase head domain are mentioned, please annotate these in Figure 1A.
- Figure 1D: the kDa scale is missing from the H3 WB.
- What is the scale indicated by different colors in Fig. 2A?

- Line 189: it would also be interesting how many peaks are "conserved" between the different time points studied, so not only to compare the gene lists of closest genes but also the intersecting peaks and then the closest genes to the intersecting peaks.
- What is the distribution of the peaks over diverse genetic elements, such as gene bodies, introns, convergent/ divergent/ tandem intergenic regions? In yeast, cohesion is particularly enriched in convergent intergenic regions, so it would be interesting to see how this behaves in P. falciparum.
- Line 130 intra-chromosomal interactions (word missing)
- Contrary to Figure 1D, the WB in Figure 3A indicates strong expression of Smc3 in rings. Please comment on this discrepancy.
- What time point after glucosamine addition represents the WB in Fig. 3A?
- Line 233 / Suppl Figure 3: Isn't it a bit concerning that the untreated control parasites at 24 hpi statistically corresponded to 18-19 hpi? And to what timepoint did the wt parasites correspond?
- Line 264: "whether naturally or via knockdown" - the meaning of this sentence is not entirely clear
- Figure 4 Legend: A, B, C etc. are mixed up.
- Figure 4D: the differences seem to be marginally significant, even not significant at all (q=0.8) for gap45 at 12hpi.
- Figure 4F shows FACS data using SYBR green as a DNA stain. The authors could exploit this data to look at the relative DNA content per cell as a measure of parasite stage, since more mature parasites will have more DNA (mean fluorescence intensity). How did the corresponding parasite cultures look in Giemsa smears?
- Are RNAseq replicates biological replicates from independent experiments or technical replicates?
- Why does the number of genes analysed for differential gene expression differ between the comparisons?
- Line 372: Do you mean the proteins or the genes? AP2-I has a peak at 24 hpi and 36 hpi, and its interacting AP2 factor Pf3D7_0613800 at all time points.
- Line 480: no aldolase was shown.
- Line 838: include GO analysis in methods

Referees cross-commenting

All reviewers agree that the paper addresses an important topic and provides convincing evidence for enrichment of the cohesin component Smc3 at P. falciparum centromers. In contrast, evidence for a function of Smc3 as a transcriptional repressor of genes in the first part of the parasite life cycle is less well supported. All reviewers agree that the statistical significance of the ChIP experiments needs to be impoved by including biological replicates. In addition, the phenotype of the conditional knock-down should be analysed in more detail by clarifying whether faster cell cycle progression or higher invasion rate are responsible for the observed growth adavantage. Inclusion of transcriptional data from a later time point in addition to the presented data for 12 hpi and 24 hpi was also requested by all reviewers. Finally, several inconsistencies require clarification.

2. Significance:

Significance (Required)

The paper addresses the function of the cohesin complex in gene regulation of malaria parasites for the first time. Due to the conserved nature of the complex, the data may be interesting for a broad audience of scientists interested in nuclear biology and cell division/ gene regulation.

3. How much time do you estimate the authors will need to complete the suggested revisions:

Estimated time to Complete Revisions (Required)

(Decision Recommendation)

Between 3 and 6 months

4. *Review Commons* values the work of reviewers and encourages them to get credit for their work. Select 'Yes' below to register your reviewing activity at Web of Science Reviewer Recognition Service (formerly Publons); note that the content of your review will not be visible on Web of Science.

Web of Science Reviewer Recognition

Yes

Review #3

1. Evidence, reproducibility and clarity:

Evidence, reproducibility and clarity (Required)

Summary:

In the presented manuscript by Rosa et al. the authors investigate the longstanding question of how P. falciparum achieves the tight transcriptional regulation of its genome despite the apparent absence of many canonical sequence specific transcription factor families found in other eukaryotes. To do this the authors investigate the role of the spatial organization of the genome in this context, by performing a functional

characterization of the conserved cohesion subunit SMC3 and its putative role in transcriptional regulation in P. falciparum.

Using Cas9 mediated genome editing the authors generated a SMC3-3xHA-glmS parasite line, which they subsequently used to show expression of the protein over the asexual replication cycle by western blot and IFA analysis. In addition, using co-IP experiments coupled with mass spectrometry they identified the additional components of the cohesion complex also found in other eukaryotes as interaction partners of SMC3 in the parasite, thereby confirming the presence of the conserved cohesin complex in P. falciparum.
By using a combination of ChIP-seq and RNA-seq experiments in SMC3 knockdown parasites the authors furthermore show that a reduction of SMC3 resulted in the up-regulation of a specific set of genes involved in invasion and egress in the early stages of the asexual replication cycle and that this up-regulation in transcription is correlated with a loss of SMC3 enrichment at these genes. From these observations the authors conclude, that SMC3 binds dynamically to a subset of genes and works as a transcriptional repressor, ensuring the timely expression of the bound genes.

Overall, the presented data is intriguing, of high quality and very well presented. However, there are some points, which should be addressed to bolster the conclusions drawn by the authors.

Major points:

I was not able to find the deposited datasets in the BioProject database under the given accession number. This should obviously be addressed and would have been nice to be able to have a look at these datasets also for the review process.

SMC3-ChIP-seq experiments:

"168 were bound by SMC3 across all three time points (Fig. 2D). However, most SMC3-bound genes showed a dynamic binding pattern, with a peak present at only one or two time points (Fig. 2B,D)."

Here it would be interesting to actually have more than one replicate of each of these ChIP-seq time points. This could provide a better idea of how "dynamic" these binding patterns actually are. Furthermore, I was missing a list of these 168 genes, which are constantly bound by SMC3. Anything special about those? What actually happens to this subset of genes in the SMC3 knockdown parasites? Do they show similar transcriptional changes?

SMC3-knockdown experiments:

In Sup. Fig. 1 there is a double band in the HA-western blot in the 2nd cycle -GlcN. sample. This second band is absent in all other HA-western shown. Have the authors

any idea where that second band comes from?

In Figure 3A, the WB data shown is slightly contrasting the RNA-seq quantification (3B). The knock-down on protein level seems to be stronger in the 12 hpi samples here than in the 24 hpi samples. Although the band for HA-SMC3 is stronger at the 12 hpi TP there's no band visible in the + GlcN. sample. There's however in the 24 hpi samples. Could the authors comment on this?

"Comparison of our RNA-seq data to the time course transcriptomics data from (Painter et al., 2018) revealed that SMC3 depletion at 12 hpi caused downregulation of genes that normally reach their peak expression in the trophozoite stage (18-30 hpi), with the majority of upregulated genes normally reaching their peak expression in the schizont and very early ring stages (40-2 hpi) (Fig. 3E). At 24 hpi, a similar trend is observed, with most downregulated genes normally peaking in expression in trophozoite stage (24-32 hpi) and the majority of upregulated genes peaking in expression at very early ring stage (2 hpi) (Fig. 3F)."

I'm not fully convinced by these presented results/conclusions. This dataset would greatly benefit from the inclusion of additional later time points. The presented upregulation of the egress and invasion related genes is hard to pinpoint to be a direct effect of transcriptional changes due to the SMC3 knockdown. While there's a slight upregulation of these genes they still seem to be regulated in their normal overall transcriptional program as shown in Figure 4D/E. So the changes could in theory also be explained by the differences in cell cycle progression which are present between +/- GlcN. cultures (Sup. Fig. 3). The presented normalization to the microarray data is a well-established practice to correct for this but, as presented seems to have its limitation with these parasite lines (line 233, glucosamine treated parasites harvested at 24 hpi correspond statistically to approximately 18-19 hpi (Supp. Fig. 3).) By including additional later time points, one could actually follow the expression profiles over the whole cycle and elucidate if there's an actual transcriptional up-regulation of the genes, or if the + GlcN. parasites show a faster cell cycle progression, with a shifted peak expression timing compared to the - GlcN. parasites.

"These genes show SMC3 enrichment at their promoter regions at 12 and 24 hpi, but not at 36 hpi (Fig. 4C), and depletion of SMC3 resulted in upregulation at both 12 and 24 hpi (Fig. 4D). Comparison of the SMC3 ChIP-seq data with published Assay for Transposase-Accessible Chromatin using sequencing (ATAC-seq) data (Toenhake et al., 2018) and mRNA dynamics data (Painter et al., 2018) from similar time points in the IDC revealed that SMC3 binding at the promoter regions of these genes inversely correlates with chromatin accessibility (Fig. 4C) and their mRNA levels (Fig. 4E), which both peak in schizont stages. These data are consistent with a role of SMC3 in repressing this gene subset until their appropriate time of expression in the IDC."

The presented correlations certainly make an intriguing point towards the authors conclusion that SMC3/cohesin depletion from the promoter regions of the genes results in a de-repression of these genes and their transcriptional activation. However, the SMC3 knockdown is not complete and only up to 69% as presented on RNA level in

these parasites. Therefore a control experiment which needs to be done is to actually show the loss of SMC3 from the presented activated example genes in the knockdown parasites. This could easily be done by ChIP-qPCR or even ChIP-seq, to get a global picture of the actual changes in SMC3 occupation in the knockdown parasites in correlation with changes in transcript levels.

"These data suggest that SMC3 knockdown results in a faster progression through the cell cycle or a higher rate of egress/invasion."

The authors could greatly strengthen their conclusions by investigating this thoroughly. Pinpointing the observed phenotype to an actual increase in invasion or egress would add to the authors main conclusion that the loss of SMC3 de-regulates the timing of gene expression for these invasion related genes thereby increasing their transcript levels and thus leading to a higher rate of egress/invasion.
To determine cell cycle progression simple comparisons between DNA content using a flow cytometer at timepoints together with visual inspection of Giemsa stained blood smears would give a ggod indication towards changes in cell cycle progression. In addition invasion/egress assays by counting newly invaded rings per schizont could reveal, if there are changes in the rate of egress/invasion upon SMC3 knockdown.

Minor points:

In the MM section on the Cas9 experiments it says dCas9 where it should be Cas9 (line 425)

It would be great to add which HP1 antibody was used in which dilution in the IFAs to the MM section.

In Figure 4C for the gap45 gene there's is some green peak floating around which should not be there.

2. Significance:

Significance (Required)

The manuscript investigates a very timely topic by trying to uncover new molecular mechanisms of transcriptional regulation in P. falciparum. Investigating the role of the cohesin complex/SMC3 in this context provides valuable new insights to the field. While the first part with the description of the SMC3 cell line and the co-IP experiments largely confirms published data on the existence and composition of the cohesin complex in Plasmodium and its enrichment at the centromeres, the second part is especially intriguing since it investigates the molecular function of SMC3 in more detail. The results pointing to a role of SMC3/cohesin as a transcriptional repressor are of great interest to the field and will open up new concepts for future investigation.

Audience:

The work is particularly interesting for people interested in gene regulatory processes in Plasmodium and Apicomplexan parasites in general. At the same time it also nicely points towards shared principles of gene regulation to other eukaryotes in relation to the spatial organization of the genome making the work also very interesting for a broader audience with interest in the general principles of gene regulatory processes in eukaryotic organisms.

Expertise:

P. falciparum epignetics and chromatin biology / gene regulation / Cas9 gene editing

3. How much time do you estimate the authors will need to complete the suggested revisions:

Estimated time to Complete Revisions (Required)

(Decision Recommendation)

Between 3 and 6 months

4. *Review Commons* values the work of reviewers and encourages them to get credit for their work. Select 'Yes' below to register your reviewing activity at Web of Science Reviewer Recognition Service (formerly Publons); note that the content of your review will not be visible on Web of Science.

Web of Science Reviewer Recognition

No

Manuscript number: RC- 2022-01595
Corresponding author(s): Jessica, Bryant

1. General Statements

Dear Editor,

We are writing to submit a full revision of our manuscript, “Cohesin is involved in transcriptional repression of stage-specific genes in the human malaria parasite”. We appreciate that all reviewers found that our work addresses an important topic and is of interest to a broad audience. We thank them for having raised constructive criticism to fortify the main message and improve the clarity of the manuscript.

We believe that we have thoroughly addressed the concerns of the reviewers, especially with regard to 1) performing another SMC3 chromatin immunoprecipitation and sequencing (ChIP-seq) replicate and control, 2) including a later time point for the transcriptional data, and 3) performing additional characterization of the growth phenotype of the SMC3 conditional knockdown.

1. We have performed the SMC3 ChIP-seq with an additional clone of SMC3-3HA-*glmS* parasites at all time points sampled with the original clone. We compared peaks between the two clones at each time point, which showed significant overlap. The overlapped peaks from both clones were then subjected to GO enrichment analysis and analyzed for distribution over different genomic features. Overall, our conclusions from the previous version of the manuscript are unchanged with regard to the SMC3 ChIP-seq analysis. In addition, we provide evidence that our SMC3 ChIP was specific and robust by comparing our SMC3 ChIP-seq peaks to peaks of a control ChIP-seq using the same anti-HA antibody in the wildtype parent strain, which showed no significant overlap.
2. We now provide transcriptional data for a time point later in the parasite cell cycle. We did not include this data in our original manuscript due to the fact that it was not possible to normalize it as we did with the other time points. However, these data demonstrate that the up-regulation of late stage genes that we saw upon SMC3 knockdown in interphase parasites is not present in late-stage parasites.
3. One significant change to our original manuscript is the phenotypic characterization of the growth of SMC3 knockdown parasites. We have repeated the growth curve analysis with more clones and no longer observe an increase in parasite growth with SMC3 knockdown. We believe that with the knockdown system we are using, SMC3 is not depleted to an extent that results in a significant growth phenotype, which has also been seen for cohesin components in other model eukaryotes studied. Moreover, SMC3 is most likely one of several proteins organizing centromeres and controlling the transcription of invasion and egress genes.

These new experiments and analyses have greatly strengthened our manuscript. We believe that the conclusions from our original version of the manuscript remain the same and that our data support an exciting new role for cohesin in the temporal transcriptional regulation of stage-specific genes.

Please find attached a point-by-point response to the reviewers' comments to aid in your editorial decision.

Thank you for your consideration of our revised manuscript,

Jessica Bryant

2. Point-by-point description of the revisions

Reviewer #1
(Evidence, reproducibility and clarity (Required)):

Summary
The present work by Rosa et al., provides convincing data about the presence and functional relevance of the cohesin complex in Plasmodium falciparum blood stages. In accordance with other organisms, the composition of the cohesin complex containing SMC1, SMC3 RAD21 and putatively STAG could be confirmed via pulldown and mass spectrometry. Basic characterization of endogenous tagged SMC3 demonstrated the expression and nuclear localization during IDC, as well as the relatively stable accumulation at centromeric regions, consistent with the known cohesin function in chromatid separation. Furthermore, dynamic and stage-dependent binding to intergenic regions observed in ChIPseq and major transcriptome aberrations upon knockdown of SMC3 (<70% mRNA reduction via glmS-system) suggest an important function in transcriptional regulation. The underlying mechanism of gene regulation by cohesin via the creation of chromatin clusters or DNA loops as well as its specificity to target sites remain speculation.

Major criticism

• *Accuracy of ChIPseq with only one replicate per time point (and lacking negative controls without antibody) is not convincing. The authors should include more replicates.*

Response: As we regularly perform ChIP-seq experiments in the lab, we have generated multiple negative control datasets. In our opinion, the most stringent negative control for an HA-tagged protein is performing ChIP with an HA antibody in a WT strain. We have recently published an in-depth analysis of this (and other) negative ChIP-seq controls (Baumgarten & Bryant, 2022, https://doi.org/10.12688/openreseurope.14836.2). We show in this publication that non-specific ChIP-seq experiments (such as negative controls) result in an over-representation of HP1-heterochromatinized regions due to differences in sonication efficiency of heterochromatin and technical challenges with mapping regions with high levels of homology. In the anti-HA in WT ChIP negative control (performed at 12hpi), we do not see any enrichment at centromeric regions, but rather at heterochromatinized regions where clonally variant gene families are located. We performed peak calling analysis and found no significant overlap between the negative control ChIP-seq and the SMC3-3HA ChIP-seq data at 12hpi.

In addition, we have now performed a second biological replicate of the SMC3-3HA ChIP-seq with a different clone at all time points. We compared this data to that from the original clone and found significant overlap of the peaks called (see what is now Table 4 and Supp. Fig. 3A). We generated a stringent list of peaks that were shared between both clones at each time point and repeated all downstream analyses (see what are now Tables 5-8). We found that our conclusions were largely unchanged. Text describing these experiments and analyses have been added throughout the results section.

• *Proposed mechanism of repressive effect of SMC3 early in IDC on genes, that get de-repressed in late stages: To claim this mode of function, it would be necessary to include a KD on late stage parasites. If there is an early repressive role of SMC3, upregulated genes should not be affected by late SMC3-KD.*

Response: To be clear, we are most interested in the transcriptional role of SMC3 during interphase, where results are not confounded by its potential role in mitosis. However, we did collect a 36hpi time point in the SMC3-3HA-*glmS* and WT strain, with and without glucosamine. We have added this last time point and the WT data from the other two time points to the manuscript (see Tables 11-13). Unfortunately, and for reasons unknown, the WT replicates treated with glucosamine showed a significantly advanced "transcriptional age" compared to the other replicates at 36hpi (see what is now Supp. Fig. 5B). Thus, we did not feel comfortable performing the RNA-seq analysis as we did with the other two time points (i.e. subtracting up- and down-regulated genes from the WT control from the SMC3-3HA-*glmS* data sets). We have added this information to the results section (Lines 256 and 261). As the WT parasites treated with glucosamine were approximately 8 hours in advance of the untreated WT parasites for the 36hpi time point, any up- and down-regulated genes might have been due to differences in the cell cycle rather than due to glucosamine treatment. The *glmS* system of inducible knockdown is widely used in *P. falciparum*; however, to our knowledge, no lab has investigated whether glucosamine treatment affects transcription in wildtype cells over the course of the IDC. Thus, for accurate phenotypic characterization of any protein with this system with regard to transcriptomics, we thought it was important to provide an RNA-seq dataset to define the cohort of genes affected by glucosamine treatment in WT parasites. We hope that our study will

demonstrate the importance of using stringent controls when using inducible knockdown systems.

To address the question of whether genes that are upregulated upon depletion of SMC3 at early stages are affected at the 36hpi time point, we performed differential expression analysis of the SMC3-3HA-*glmS* parasites with and without glucosamine at 36hpi (we have added this data in Table 11). Again, significantly up- and down-regulated genes were not filtered using the WT dataset. With this analysis, we see only three genes from the list of invasion-related genes (Hu et al., 2010) that are up-regulated, but none of them have a significant *q*-value (Tab 5 of Table 18). Thus, depletion of SMC3 in late stage parasites does not lead to up-regulation of the same genes that are upregulated at 12 and 24hpi. We have added this information to the text (Line 273).

Furthermore, the hypothesized repressive effect of SMC3 does not explain the numerous genes downregulated in KD.
Response: As we state on line 350, we do not observe enrichment of SMC3 at downregulated genes, suggesting an indirect or secondary effect of SMC3 KD on these genes.

• *Due to the fact, that the KD was induced at the exact same timepoint and analysed 12h and 24h after induction it is possible that identified, differentially expressed genes at 24h are not directly regulated by SMC3, but rather due to a general deregulation of gene expression. Did the authors attempt to analyse gene expression upon induction at ring, trophozoite and schizont stage?*
Response: As we state on line 230, in order to achieve SMC3 KD at the protein level, we had to treat the parasite with glucosamine for two cell cycles (approximately 96 hours). After two cell cycles of glucosamine treatment, the parasites were tightly synchronized and sampled 12 and 24 hours later. Thus, SMC3 KD takes place over the course of multiple days, but parasites are collected after stringent synchronization. Giemsa staining and bioinformatic analysis (line 250) of the RNA-seq data from parasites (with or without glucosamine) harvested at 12 and 24 hpi show that these parasites were synchronous and that there were no gross differences in genome-wide transcript levels. It is certainly possible that differentially expressed genes at 12 or 24hpi are not directly regulated by SMC3, and this is precisely why we perform ChIP-seq of SMC3: to provide evidence of direct involvement via binding, as stated on line 281.

• *Based on rapid parasite growth, the authors hypothesize a higher invasion rate due to upregulation of invasion genes. This hypothesis is not supported by quantitative invasion assays or quantification of invasion factors on the protein level. An alternative explanation could be a shorter cell cycle (<48h), as the different cell cycle progression estimation of KD/WT could indicate (SuppFigure 3). Giemsa-stain images of KD/WT parasites should be included to show normal stage development over time.*
Response: We have repeated the growth curve analysis with additional clones and no longer observe a growth phenotype in the SMC3 knockdown condition. We have added images of Giemsa-stained parasites from the knockdown time course we performed to what is now Supp.

Fig. 5A. We see no obvious differences in cell morphology caused by glucosamine treatment in the WT or SMC3-3HA-glmS parasites.

• *Correlation of SMC3-occupancy/ATAC/expression profile of the exemplary genes rap2 and gap45 (Figure 4C,D,E): is this representative for all upregulated genes?*
Response: SMC3 occupancy shown at *rap2* and *gap45* is representative for all upregulated genes (see Fig. 4A and B). It is difficult to provide a general representation of the average expression profiles of all up-regulated genes over the course of the IDC, but Fig. 3E shows that the vast majority of up-regulated genes normally reach their peak expression in late stage parasites. With regard to ATAC-seq profiles, we have performed a metagene analysis of chromatin accessibility (data taken from (Toenhake et al., 2018)) at all up-regulated genes at time points that closely correspond to the time points used in our study: 15, 25, and 35, and 40 hpi (new Fig. 4C). This metagene analysis confirms what we observe at individual genes: increasing chromatin accessibility over the course of the IDC at these genes' promoters. While metagene analyses offer important information, we always try to show the raw data (as in new Figs. 4D-F) from individual examples as proof of principle.

• *Given that SMC3 appears to be not essential for parasite growth, the authors could generate a null mutant for SMC3, which might allow for easier analysis of differences in gene regulation, cell cycle progression and/or invasion efficiency.*
Response: As we explain on line 327, very little cohesin is required for normal growth and/or mitosis in our study and two studies in *S. cerevisiae* and *D. melanogaster*. However, SMC3 is essential in *S. cerevisiae*. We were unable to knock out SMC3, and a recent mutagenesis study suggests that SMC3 and SMC1 are essential to the parasite during the intraerythrocytic developmental cycle (Zhang *et al*. Science, 2018). This is why we chose an inducible knockdown system.

Reviewer #1 (Significance (Required)):

Own opinion
The authors provide a basic characterization of the cohesin component SMC3 using NGS methods to investigate chromatin binding sites and its potential influence on gene expression.
Response: We respectfully disagree that our study offers only a basic characterization of SMC3. We combine IFA, mass spectrometry, and both ChIP-seq and RNA-seq of SMC3 across the entire intraerythrocytic developmental cycle to provide the most detailed and comprehensive functional analysis of SMC3 in *P. falciparum* to date.

The localisation of SMC3 at centromers as described previously (Batugedara 2020) was confirmed. However, the dynamic binding to other regions in the genome, potentially mediated by other proteins, could not be resolved unequivocal with only one replicate of ChIPseq per time point.
Response: With regard to the replicates for ChIP-seq, please see our response to this same point above.

Similarly, the RNAseq data demonstrate the relevance of SMC3 for gene expression, but no clear picture of a regulatory mechanism can be drawn at his point. Lacking information about the mode of binding as well as the setup of transcriptome analysis (only two time-shifted sampling points after simultaneous glmS treatment for 96h resulting in incomplete knockdown) cannot definitely elucidate, if SMC3/cohesin is a chromatin factor that affects transcription of genes in general or a specific repressor of stage-specific genes.
Response: We agree that we have not established a regulatory mechanism for how SMC3 achieves binding specificity. However, the combination of inducible knockdown (as SMC3 is essential to the cell cycle) and differential expression analysis with ChIP-seq from the same time points across the intraerythrocytic developmental cycle is the most stringent and standard approach in the field of epigenetics for determining the direct role of a chromatin-associated protein in gene expression. We provide a detailed explanation of how the transcriptome analysis was set up in the Results (lines 229-234) and Materials and Methods (lines 715-719) section. With regard to our sampling points being "time-shifted," we provide bioinformatic analysis (line 246-251, what is now Supp. Fig. 5B) of the RNA-seq data from untreated and glucosamine-treated parasites showing highly similar "ages" with regard to progression through the intraerythrocytic developmental cycle. While we of course also monitor progression through the cell cycle with Giemsa staining (Supp. Fig. 5A), this bioinformatic analysis is the most stringent method of determining specific times in the cell cycle.

The work will be interesting to a general audience, interested in gene regulation and chromatin remodelling

The reviewers are experts in Plasmodium cell biology and epigenetic regulation.

Reviewer #2
(Evidence, reproducibility and clarity (Required)):

Rosa et al, Review Commons
The manuscript by Rosa et al. addresses the function of the cohesion subunit Smc3 in gene regulation during the asexual life cycle of P. falciparum. Cohesin is a conserved protein complex involved in sister chromatin cohesion during mitosis and meiosis in eukaryotic cells. Cohesin also modulates transcription and DNA repair by mediating long range DNA interactions and regulating higher order chromatin structure in mammals and yeast. In P. falciparum, the Cohesin complex remains largely uncharacterized. In this manuscript, the authors present mass spectrometry data from co-IPs showing that Smc3 interacts with Smc1 and a putative Rad21 orthologue (Pf3D7_1440100, consistent with published data from Batugedara et al and Hilliers et al), as well as a putative STAG domain protein orthologue (PF3D7_1456500). Smc3 protein appears to be most abundant in schizonts, but ChIPseq indicates predominant enrichment of Smc3 in centromers in ring and trophozoite stages. In addition, Smc3 dynamically binds with low abundance to other loci across the genome; however, the enrichment is rather marginal and

only a single replicate was conducted for each time point making the data interpretation difficult. Conditional knock-down using a GlmS ribozyme approach indicates that parasites with reduced levels of Smc3 have a mild growth advantage, which is only evident after five asexual replication cycles and which the authors attribute to the transcriptional upregulation of invasion-linked genes following Smc3 KD. Indeed, Smc3 seems to be more enriched upstream of genes that are upregulated after Smc3 KD in rings than in downregulated genes, indicating that Smc3/cohesin may have a function in supressing transcription of these schizont specific genes until they are needed. The manuscript is concise and very well written, however it suffers from the lack of experimental replicates for ChIP experiments and a better characterization of the phenotype of conditional KD parasites.

Major comments

• In the mass spectrometry analysis, many seemingly irrelevant proteins are identified at similar abundance to the putative rad21 and ssc3 orthologues, and therefore the association with the cohesion complex seems to be based mostly on analogy to other species rather than statistical significance. Hence, it would be really nice to see a validation of the novel STAG domain and Rad21 proteins, for example by Co-IP using double transgenic parasites.

Response: While our IP-MS data did not yield high numbers of peptides, the top most enriched proteins were SMC3 and SMC1. As we state on line 157, two previous studies have already shown a robust interaction between SMC1, SMC3, and RAD21 in *Plasmodium*, supporting the existence of a conserved cohesin complex. While the identification of the STAG domain-containing protein is interesting, the purpose of our IP-MS was less about redefining the cohesin complex in *P. falciparum* and more about confirming that the epitope-tagged SMC3 we generated was incorporated correctly into the cohesin complex and was specifically immunoprecipitated by the antibody we later use for western blot, immunofluorescence, and ChIP-seq analyses. However, to validate the results of ours and others' mass spectrometry results, we generated two new parasite strains – SMC1-3HA-dd and STAG-3HA-dd – and an antibody against SMC3 (see what is now Supp. Fig. 1). We performed co-IP and western blot analysis with these strains and show an interaction between SMC1 and SMC3 and STAG and SMC3 (see what is now Supp. Fig. 2). This information has been added to the manuscript on lines 162-167.

• The ChIPseq analysis presented here is based on single replicates for each of the three time points. The significance cutoffs for the peaks are rather high ($q < 0.05$). Therefore, the relevance of the marginally enriched dynamic peaks (average relative enrichment of <1.2 fold for genes upregulated in rings 12 hpi in Figure 4A and B) does not appear to be very robust. Even in ChIPseq experiments using non-immune IgG, hundreds of peaks are usually called with MACS2 with a similar magnitude. So, to substantiate the data for extra-centromeric peaks convincingly, replicates and more stringent statistics are necessary. In addition, the authors should also compare their data to published PfSmc3 ChIP data from Batugedara et al 2020 (GSE116219).

Response: In our experience, a significance cutoff of FDR < 0.05 for peak analysis is fairly standard. The average relative SMC3 enrichment shown in Figures 4A and B are independent

of the peak calling analysis. Figures 4A and B show average ChIP/Input values for all genes considered (i.e. genes that are significantly up-regulated in the SMC3 KD), regardless of whether a significant peak was called for each gene.

As we regularly perform ChIP-seq experiments in the lab, we have generated multiple negative control datasets. In our opinion, the most stringent negative control for an HA-tagged protein is performing ChIP with an HA antibody in a WT strain. We have recently published an in-depth analysis of this (and other) negative ChIP-seq controls (Baumgarten & Bryant, 2022, https://doi.org/10.12688/openreseurope.14836.2). We show in this publication that non-specific ChIP-seq experiments (such as negative controls) result in an over-representation of HP1-heterochromatinized regions due to differences in sonication efficiency of heterochromatin and technical challenges with mapping regions with high levels of homology. In the anti-HA in WT ChIP negative control (performed at 12hpi), we do not see any enrichment at centromeric regions, but rather at heterochromatinized regions where clonally variant gene families are located. We performed peak calling analysis and found no significant overlap between the negative control ChIP-seq and the SMC3-3HA ChIP-seq data at 12hpi.

In addition, we have now performed a second biological replicate of the SMC3-3HA ChIP-seq with a different clone at all time points. We compared this data to that from the original clone and found significant overlap of the peaks called (see what is now Table 4 and Supp. Fig. 3A). We generated a stringent list of peaks that were shared between both clones at each time point and repeated all downstream analyses (see what are now Tables 5-8). We found that our conclusions were largely unchanged. Text describing these experiments and analyses have been added throughout the results section.

The SMC3 ChIP from Batugedara et al., 2020 was performed with an in-house generated antibody (not a commercially available, widely validated antibody as we use) at a single time point in the IDC: trophozoites. Batugedara et al. performed one replicate and did not have an input sample for normalization. Rather, it seems that they incubated beads, which were not bound by antibody or IgG, with their chromatin and used any sequenced reads from this beads sample to subtract from their SMC3 ChIP signal as means of normalization. According to ENCODE ChIP-seq standards, this is not a standard nor stringent way of performing ChIP-seq and the subsequent analysis. Because they did not generate a dataset for their ChIP input, it is not possible to call peaks as we do in our study and compare those peaks with ours.

• *The authors argue that during schizogony, cohesin may no longer be required at centromers, explaining the low ChIPsignal at this stage (Line 301). However, during schizogony parasites undergo repeated rounds of DNA replication (S-phase) and mitosis (M-phase) to generate multinucleated parasites; and concentrated spots of Smc3 are observed in each nucleus in schizonts by IFA. In turn, the strong presence of Smc3 at centromers in ring stage parasites is surprising, particularly since the Western Blot in Figure 1D shows most expression of Smc3 in schizonts and least in rings; and Smc3 is undetectable in rings by IFA. Yet, the ChIP signal shows very strong enrichment at centromers, long before S phase produces sister chromatids. What could be the reason for this discrepancy? Again, ChIP replicates and controls would be helpful in distinguishing technical problems with the ChIP from biologically relevant differences.*

Response: We discuss in lines 337-342 not that cohesin is no longer required at centromeres during schizogony, but that its removal from centromeres may be required specifically for separation of sister chromatids, as is seen in other eukaryotes. We also discuss that the unique asynchronous mitosis in *Plasmodium* may lead to a mixed population of parasites at the time point sampled where there may be some centromeres with SMC3 present and some where it is absent to promote sister chromatid separation. Even though SMC3 may be evicted from centromeres to promote sister chromatid separation, it is likely re-loaded onto centromeres once this process is complete. This is most likely why we see foci of SMC3 in each nucleus of mature schizonts by IFA. With regard to the discrepancy between SMC3 levels in rings seen in total nuclear extracts (by western blot) and at centromeres (by ChIP-seq): the total level of a protein in the nucleus does not necessarily dictate the genome-wide binding pattern or the level of enrichment of that protein at specific loci in the genome. Moreover, if one molecule of SMC3 binds to each centromere, 14 molecules would be needed in a ring stage parasite while over 500 would be needed in a schizont (assuming that there are ~36 merozoites present). SMC3 binds to centromeres in interphase cells in other eukaryotes as well, and we speculate that this binding may play a role in the nuclear organization of centromeres, as we discuss starting on line 333.

• *It is surprising that a conserved protein like Smc3 shows such a subtle phenotype, given that it is predicted to be essential and its orthologues have a function in mitosis. Generally, only limited data are presented to characterize the Smc3 KD parasites, and more detail should be included. For example validation of the parasite line using a PCR screen for integration and absence of wt, parasite morphology after KD, and/or analysis of the KD parasites for cell cycle status.*
Response: First, we have repeated our growth curve analysis several times and with more clones and have concluded that there is not a significant growth phenotype in SMC3 KD parasites (see what is now Supp. Fig. 4B). As we discuss on line 342, very little intact cohesin complex seems to be required for normal growth and mitosis in *S. cerevisiae* and *D. melanogaster*, which is probably why we do not see an obvious growth or morphological phenotype. Because we could not generate SMC3 knockout parasites, there may be just enough SMC3 left to perform its vital function in our KD strain. We have added PCR data to demonstrate integration of the 3HA tag- and glmS ribozyme-encoding sequence in the clonal strains we are using for all experiments (see what is now Supp. Fig. 1A). Sanger sequencing was performed on these PCR products to confirm correct sequences. We also added images of Giemsa-stained parasites in untreated and glucosamine-treated parasites at all time points to demonstrate a lack of an obvious morphological phenotype in SMC3 KD parasites (see what is now Supp. Fig. 5A).

• *Synchronization was performed at the beginning of the growth time course, which would be expected to result in a stepwise increase in parasitemia every 48 hours; however, the parasitemia according to Fig. 4F rises steadily, which would indicate that the parasites are actually not very synchronous.*

Response: We did indeed tightly synchronize these parasites and hope that the stepwise increase in parasitemia is seen better in our new growth curve analysis (see what is now Supp. Fig. 4B).

• *The question of whether Smc3 causes a shorter parasite life cycle (quicker progression) or more invasion is important and could be experimentally addressed by purifying synchronous schizont stage parasites and determining their invasion rates as well as morphological examination of the Giemsa smears over the time course.*
Response: We have repeated our growth curve analysis several times and with more clones and have concluded that there is not a significant growth phenotype in SMC3 KD parasites (see what is now Supp. Fig. 4B).

• *Please also compare Smc3 transcriptional levels in transgenic parasites to those in wt parasites to rule out that the genetic modification has lead to artificial upregulation of Smc3 transcription.*
Response: We have added this data to what is now Supp. Fig. 4C, showing that there is no significant difference in SMC3 transcript levels between WT and SMC3-3HA-*glmS* strains. We have added this information to the text of the manuscript (Line 243). As we also generated an SMC3 antibody, we could demonstrate that there is no appreciable difference in SMC3 protein levels between WT and SMC3-3HA-glmS strains (see what is now Supp. Fig. 1D).

• *According to Figure S2, even more genes were deregulated at the 12 hpi time point in the WT parasites than in Smc3 parasites, and even to a much higher extent. What "transcriptional age" did the WT control parasites have at each time point?*
Response: We have now included the transcriptional age of all strains, replicates, and treatments in what is now Supp. Fig. 5B. At the 12 hpi time point in particular, regardless of glucosamine treatment, the SMC3-3HA-*glmS* and WT parasites were highly synchronous. The only large discrepancy we see in transcriptional age is between untreated and glucosamine-treated WT parasites at 36 hpi, which is why we did not include this time point in our transcriptional analysis. We were also surprised by the number of genes that were de-regulated with simple glucosamine treatment. The *glmS* system of inducible knockdown is widely used in *P. falciparum*; however, to our knowledge, no lab has investigated whether glucosamine treatment affects transcription in wildtype cells over the course of the IDC. Thus, for accurate phenotypic characterization of any protein with this system with regard to transcriptomics, we thought it was important to provide an RNA-seq dataset to define the cohort of genes affected by glucosamine treatment in WT parasites. We hope that our study will demonstrate the importance of using stringent controls when using inducible knockdown systems.

• *A negative correlation with transcription is well established in S. cerevisiae, particularly at inducible genes. How does Smc3 enrichment generally look like for genes that show maximal expression at each of the time point?*
Response: We have performed a metagene analysis of SMC3 enrichment at all genes at each respective time point, which we divided into quartiles of expression based on their FPKM values

in the RNA-seq data from the corresponding time point in untreated SMC3-3HA-glmS parasites. This quartile analysis considers *all* genes, including genes that are not transcribed at all and regardless of whether a gene has a significant SMC3 peak or is differentially expressed upon SMC3 knockdown. At the 12 hpi time point, we do see an inverse correlation between SMC3 enrichment and gene transcription level, but this enrichment is most pronounced across genes bodies. We see the highest SMC3 enrichment at genes in the 4th (lowest) quartile category. For the other two time points, we do not see any obvious pattern of SMC3 enrichment with regard to transcriptional status.

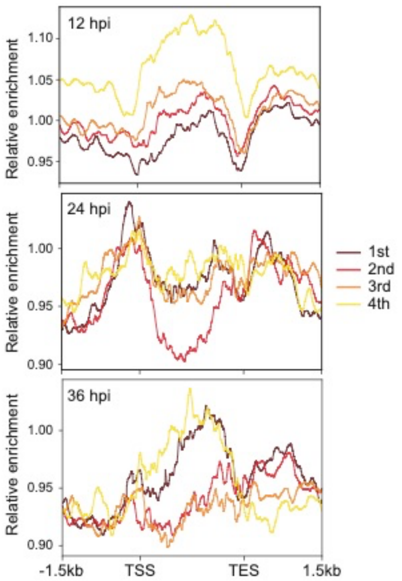

• *Line 590: according to the methods, a 36 hpi KD time point was also harvested. Why are the data not shown/analysed?*
Response: To be clear, we are most interested in the transcriptional role of SMC3 during interphase, where results are not confounded by its potential role in mitosis. However, we did collect a 36hpi time point in the SMC3-3HA-*glmS* and WT strain, with and without glucosamine. We have added this last time point and the WT data from the other two time points to the manuscript (see Tables 11-13). Unfortunately, and for reasons unknown, the WT replicates treated with glucosamine showed a significantly advanced "transcriptional age" compared to the other replicates at 36hpi (see what is now Supp. Fig. 5B). Thus, we did not feel comfortable performing the RNA-seq analysis as we did with the other two time points (i.e. subtracting up- and down-regulated genes from the WT control from the SMC3-3HA-*glmS* data sets). We have added this information to the results section (Lines 256 and 261). As the WT parasites treated with glucosamine were approximately 8 hours in advance of the untreated WT parasites for the 36hpi time point, any up- and down-regulated genes might have been due to differences in the cell cycle rather than due to glucosamine treatment. The *glmS* system of inducible knockdown is widely used in *P. falciparum*; however, to our knowledge, no lab has investigated whether glucosamine treatment affects transcription in wildtype cells over the course of the IDC. Thus, for accurate phenotypic characterization of any protein with this system with regard to

transcriptomics, we thought it was important to provide an RNA-seq dataset to define the cohort of genes affected by glucosamine treatment in WT parasites. We hope that our study will demonstrate the importance of using stringent controls when using inducible knockdown systems.

Minor Comments

• Line 103/104: the hinge domain and ATPase head domain are mentioned, please annotate these in Figure 1A.

Response: We have annotated the hinge and ATPase domains.

• Figure 1D: the kDa scale is missing from the H3 WB.

Response: We have added a kDa scale.

• What is the scale indicated by different colors in Fig. 2A?

Response: The different colors (blue, coral, and green) only represent the 12, 24, and 36hpi time points, respectively. This color scheme is used throughout the manuscript. If the reviewer is referring to the color gradation within each circos plot, this does not indicate a specific scale. The maximum y-axis value for all circos plots is 24, as indicated in the figure legend.

• Line 189: it would also be interesting how many peaks are "conserved" between the different time points studied, so not only to compare the gene lists of closest genes but also the intersecting peaks and then the closest genes to the intersecting peaks.

Response: We have added this information in Table 7 and in the manuscript starting on Line 203. Using the new dataset of consensus peaks between two replicates, there were 88 genes associated with an SMC3 peak across all three time points, most of which were close to a centromeric region.

• What is the distribution of the peaks over diverse genetic elements, such as gene bodies, introns, convergent/ divergent/ tandem intergenic regions? In yeast, cohesion is particularly enriched in convergent intergenic regions, so it would be interesting to see how this behaves in P. falciparum.

Response: We would have liked to define how many peaks were in intergenic versus genic regions of the genome, but the dataset of “genes” from PlasmoDB includes UTRs. Thus, we would need a better annotation of the genome to perform this analysis. Regardless, we calculated the average SMC3 peak enrichment (shared between both replicates) in intergenic regions between convergent and divergent genes (see what is now Supp. Fig. 3B and Table 6). As we now state in the manuscript on line 198, we see a slight enrichment in regions between convergent genes at all time points, but the differences were not significant.

• Line 130 intra-chromosomal interactions (word missing)

Response: Thank you for pointing this out. We have corrected this.

• *Contrary to Figure 1D, the WB in Figure 3A indicates strong expression of Smc3 in rings. Please comment on this discrepancy.*
Response: While extracts from all time points were run on the same western blot in Fig. 1D and thus developed for the same amount of time, this was not the case for Fig. 3A. In Fig. 3A, the samples were run on different blots and exposed for different times, so while we can compare SMC3-HA levels between – and + glucosamine for each time point, the levels at 12 hpi cannot be quantitatively compared to those at 24 or 36hpi.

• *What time point after glucosamine addition represents the WB in Fig. 3A?*
Response: The "12hpi" parasites were sampled approximately 108 hours post glucosamine addition and the "24hpi" parasites sampled approximately 120 hours post glucosamine addition. Basically, the parasites were treated with glucosamine for 96 hours, synchronized, and then harvested 12 and 24 hours later.

• *Line 233 / Suppl Figure 3: Isn't it a bit concerning that the untreated control parasites at 24 hpi statistically corresponded to 18-19 hpi? And to what timepoint did the wt parasites correspond?*
Response: We are not concerned by this, and we have included the WT parasites in what is now Supp. Fig. 5B for better comparison. In the analysis presented in Supp. Fig. 5B, regardless of glucosamine presence or absence, the differences among replicates and strains at 12 and 24hpi are, in our opinion, minimal, amounting to one or two hours of the 48-hour IDC. In our extensive experience with RNA-seq across the *P. falciparum* IDC, this synchronization is extremely tight. As we describe on line 430 of the Materials and Methods, there is a ±3 hour window in our synchronization method, meaning that parasites harvested at 24hpi could be anywhere from 21-27hpi. In addition, the dataset that was used for comparison (from Bozdech et al., 2003) was generated in 2003 in a different laboratory using different strains with microarray. While comparing more recent RNA-seq data to this classic study has become well-established practice and is useful for comparing transcriptional age between replicates and strains, it is inevitable that the calculated "hpi" from (Bozdech et al., 2003) will differ somewhat from our experimental "hpi". We have indeed seen this small discrepancy in predicted transcriptional age in several of our RNA-seq datasets (unrelated to this study) from trophozoites harvested at 24hpi.

• *Line 264: "whether naturally or via knockdown" - the meaning of this sentence is not entirely clear*
Response: We are referring to depletion of SMC3 at promoters, either naturally (i.e. lack of binding at the promoter at 36hpi that is not the result of SMC3 knockdown, as we show in Fig. 4B) or via SMC3 knockdown, which is not natural but artificial.

• *Figure 4 Legend: A, B, C etc. are mixed up.*
Response: Thank you for pointing this out. We have corrected this.

• *Figure 4D: the differences seem to be marginally significant, even not significant at all (q=0.8) for gap45 at 12hpi.*
Response: If one defines a significance cutoff of q = 0.05 (as is common practice in differential expression analyses), then the differences are significant. For a small minority of invasion genes (such as *gap45*), we do observe significance at either 12 hpi or 24 hpi, but not both. Thus, we have removed the word "significant" from the descriptions of each dataset in Tab 1 of what is now Table 18. however, we do not believe that this rules out a role for SMC3 at such a gene during interphase. What is now Table 18 offers a longer list of invasion-related genes, most of which are more "significantly" affected than *rap2* and *gap45*.

• *Figure 4F shows FACS data using SYBR green as a DNA stain. The authors could exploit this data to look at the relative DNA content per cell as a measure of parasite stage, since more mature parasites will have more DNA (mean fluorescence intensity). How did the corresponding parasite cultures look in Giemsa smears?*
Response: We have repeated our growth curve analysis several times and with more clones and have concluded that there is not a significant growth phenotype in SMC3 KD parasites (see what is now Supp. Fig. 4B). We have added images of Giemsa-stained parasites in untreated and glucosamine-treated parasites at all time points to demonstrate a lack of an obvious morphological phenotype in SMC3 KD parasites (see what is now Supp. Fig. 5A).

• *Are RNAseq replicates biological replicates from independent experiments or technical replicates?*
Response: RNA-seq replicates are technical replicates from the same parasite clone.

• *Why does the number of genes analysed for differential gene expression differ between the comparisons?*
Response: If the reviewer is referring to the discrepancy between the total number of genes for different time points [for example, between what is now Table 9 (12hpi) and Table 10 (24hpi)], this is because in the RNA-seq/differential expression analysis, there have to be reads mapping back to a gene in order for that gene to be included in the analysis. Thus, if a gene is not transcribed at a given time point in the treated or untreated samples, it will not be included in the analysis. Gene transcription fluctuates significantly over the course of the IDC, so different time points will have different total numbers of transcribed genes.

• *Line 372: Do you mean the proteins or the genes? AP2-I has a peak at 24 hpi and 36 hpi, and its interacting AP2 factor Pf3D7_0613800 at all time points.*
Response: We are referring to the genes. With the new ChIP-seq analysis including the second replicate, there are no consensus SMC3 peaks associated with *ap2-I, bdp1,* or Pf3D7_0613800 (see what is now Table 7).

• *Line 480: no aldolase was shown.*
Response: We have removed this sentence.

Review COMMONS

• *Line 838: include GO analysis in methods*
Response: We have added this.

Reviewer #2 (Significance (Required)):
The paper addresses the function of the cohesin complex in gene regulation of malaria parasites for the first time. Due to the conserved nature of the complex, the data may be interesting for a broad audience of scientists interested in nuclear biology and cell division/ gene regulation.

Reviewer #3
(Evidence, reproducibility and clarity (Required)):

Summary:

In the presented manuscript by Rosa et al. the authors investigate the longstanding question of how P. falciparum achieves the tight transcriptional regulation of its genome despite the apparent absence of many canonical sequence specific transcription factor families found in other eukaryotes. To do this the authors investigate the role of the spatial organization of the genome in this context, by performing a functional characterization of the conserved cohesion subunit SMC3 and its putative role in transcriptional regulation in P. falciparum.
Using Cas9 mediated genome editing the authors generated a SMC3-3xHA-glmS parasite line, which they subsequently used to show expression of the protein over the asexual replication cycle by western blot and IFA analysis. In addition, using co-IP experiments coupled with mass spectrometry they identified the additional components of the cohesion complex also found in other eukaryotes as interaction partners of SMC3 in the parasite, thereby confirming the presence of the conserved cohesin complex in P. falciparum.
By using a combination of ChIP-seq and RNA-seq experiments in SMC3 knockdown parasites the authors furthermore show that a reduction of SMC3 resulted in the up-regulation of a specific set of genes involved in invasion and egress in the early stages of the asexual replication cycle and that this up-regulation in transcription is correlated with a loss of SMC3 enrichment at these genes. From these observations the authors conclude, that SMC3 binds dynamically to a subset of genes and works as a transcriptional repressor, ensuring the timely expression of the bound genes.
Overall, the presented data is intriguing, of high quality and very well presented. However, there are some points, which should be addressed to bolster the conclusions drawn by the authors.

Major points:
I was not able to find the deposited datasets in the BioProject database under the given accession number. This should obviously be addressed and would have been nice to be able to have a look at these datasets also for the review process.
Response: We apologize for not giving the reviewers access. As the manuscript has been

made available as a pre-print (which includes data accession numbers), but has not yet been published, we have not activated access to the data on the database.

SMC3-ChIP-seq experiments:

"168 were bound by SMC3 across all three time points (Fig. 2D). However, most SMC3-bound genes showed a dynamic binding pattern, with a peak present at only one or two time points (Fig. 2B,D)."

Here it would be interesting to actually have more than one replicate of each of these ChIP-seq time points. This could provide a better idea of how "dynamic" these binding patterns actually are. Furthermore, I was missing a list of these 168 genes, which are constantly bound by SMC3. Anything special about those? What actually happens to this subset of genes in the SMC3 knockdown parasites? Do they show similar transcriptional changes?
Response: We have now performed a second biological replicate of the SMC3-3HA ChIP-seq with a different clone at all time points. We compared this data to that from the original clone and found significant overlap of the peaks called (see what is now Table 4 and Supp. Fig. 3A). We generated a stringent list of peaks that were shared between both clones at each time point and repeated all downstream analyses (see what are now Tables 5-8). We found that our conclusions were largely unchanged. Text describing these experiments and analyses have been added throughout the results section. Using the new dataset of consensus peaks between two replicates, there were 88 genes associated with an SMC3 peak across all three time points (see what is now Table 7). The genes that are associated with an SMC3 peak at all time points are, in general, those closest to centromeric/pericentromeric regions and show no obvious functional relationship to each other. Out of these 88 genes, four are significantly up- or downregulated at 12 hpi and 26 are significantly up- or downregulated at 24 hpi. The most significantly downregulated of these genes in both datasets is *smc3* itself.

SMC3-knockdown experiments:

In Sup. Fig. 1 there is a double band in the HA-western blot in the 2nd cycle -GlcN. sample. This second band is absent in all other HA-western shown. Have the authors any idea where that second band comes from?
Response: As the reviewer says, we do not see this second band in most of our western blots. It is possible that it is just a small amount of degradation in the lysate.

In Figure 3A, the WB data shown is slightly contrasting the RNA-seq quantification (3B). The knock-down on protein level seems to be stronger in the 12 hpi samples here than in the 24 hpi samples. Although the band for HA-SMC3 is stronger at the 12 hpi TP there's no band visible in the + GlcN. sample. There's however in the 24 hpi samples. Could the authors comment on this?
Response: With regard to the discrepancy of the knockdown and protein versus RNA level, it is quite common for transcript levels to not agree with protein levels. This is why we always

confirm a transcriptional knockdown with western blot analysis using appropriate loading controls. We are not sure why there is a more dramatic knockdown of SMC3 at 12hpi than at 24hpi, as these samples came from the same culture, but were simply harvested 12 hours apart.

"Comparison of our RNA-seq data to the time course transcriptomics data from (Painter et al., 2018) revealed that SMC3 depletion at 12 hpi caused downregulation of genes that normally reach their peak expression in the trophozoite stage (18-30 hpi), with the majority of upregulated genes normally reaching their peak expression in the schizont and very early ring stages (40-2 hpi) (Fig. 3E). At 24 hpi, a similar trend is observed, with most downregulated genes normally peaking in expression in trophozoite stage (24-32 hpi) and the majority of upregulated genes peaking in expression at very early ring stage (2 hpi) (Fig. 3F)."

I'm not fully convinced by these presented results/conclusions. This dataset would greatly benefit from the inclusion of additional later time points.
Response: To be clear, we are most interested in the transcriptional role of SMC3 during interphase, where results are not confounded by its potential role in mitosis. However, we did collect a 36hpi time point in the SMC3-3HA-*glmS* and WT strain, with and without glucosamine. We have added this last time point and the WT data from the other two time points to the manuscript (see Tables 11-13). Unfortunately, and for reasons unknown, the WT replicates treated with glucosamine showed a significantly advanced "transcriptional age" compared to the other replicates at 36hpi (see what is now Supp. Fig. 5B). Thus, we did not feel comfortable performing the RNA-seq analysis as we did with the other two time points (i.e. subtracting up- and down-regulated genes from the WT control from the SMC3-3HA-*glmS* data sets). We have added this information to the results section (Lines 256 and 261). As the WT parasites treated with glucosamine were approximately 8 hours in advance of the untreated WT parasites for the 36hpi time point, any up- and down-regulated genes might have been due to differences in the cell cycle rather than due to glucosamine treatment. The *glmS* system of inducible knockdown is widely used in *P. falciparum*; however, to our knowledge, no lab has investigated whether glucosamine treatment affects transcription in wildtype cells over the course of the IDC. Thus, for accurate phenotypic characterization of any protein with this system with regard to transcriptomics, we thought it was important to provide an RNA-seq dataset to define the cohort of genes affected by glucosamine treatment in WT parasites. We hope that our study will demonstrate the importance of using stringent controls when using inducible knockdown systems.

We performed differential expression analysis of the SMC3-3HA-*glmS* parasites with and without glucosamine at 36hpi (we have added this data in Table 11). Again, significantly up- and down-regulated genes were not filtered using the WT dataset. With this analysis, we see only three genes from the list of invasion-related genes (Hu et al., 2010) that are up-regulated, but none of them have a significant *q*-value (Tab 5 of Table 18). Thus, depletion of SMC3 in late stage parasites does not lead to up-regulation of the same genes that are upregulated at 12 and 24hpi. We have added this information to the text (Line 277).

The presented upregulation of the egress and invasion related genes is hard to pinpoint to be a direct effect of transcriptional changes due to the SMC3 knockdown. While there's a slight upregulation of these genes they still seem to be regulated in their normal overall transcriptional program as shown in Figure 4D/E.
Response: We provide evidence of a direct effect of SMC3 binding by combining differential expression analysis performed upon SMC3 knockdown with SMC3 ChIP-seq at corresponding time points. As we show in what is now Fig. 4C and D, promoter accessibility of these egress/invasion genes correlates with their transcriptional activity. However, SMC3 binding to the promoters of these same genes shows inverse correlation with their transcriptional activity (what is now Fig. 4B and D). While we believe that SMC3 does contribute to the repression of these genes at specific time points during the cell cycle, it is highly likely that SMC3 is just one protein of many that regulates these genes. Moreover, and especially since we do not see a growth phenotype in the SMC3 KD, it is possible that another protein or even SMC1 could compensate for loss of SMC3 at these promoter regions. We now state these possibilities on lines 346 383 of the Discussion.

So the changes could in theory also be explained by the differences in cell cycle progression which are present between +/- GlcN. cultures (Sup. Fig. 3). The presented normalization to the microarray data is a well-established practice to correct for this but, as presented seems to have its limitation with these parasite lines (line 233, glucosamine treated parasites harvested at 24 hpi correspond statistically to approximately 18-19 hpi (Supp. Fig. 3).)
Response: In the analysis presented in what is now Supp. Fig. 5B, regardless of glucosamine presence or absence, the differences among replicates and strains at 12 and 24hpi are, in our opinion, minimal, amounting to one or two hours of the 48-hour IDC. In our extensive experience with RNA-seq across the *P. falciparum* IDC, this synchronization is extremely tight. As we describe on lines 416-421 of the Materials and Methods, there is a ±3 hour window in our synchronization method, meaning that parasites harvested at 24hpi could be anywhere from 21-27hpi. In addition, the dataset that was used for comparison (from Bozdech et al., 2003) was generated in 2003 in a different laboratory using different strains with microarray. While comparing more recent RNA-seq data to this classic study has become well-established practice and is useful for comparing transcriptional age between replicates and strains, it is inevitable that the calculated "hpi" from (Bozdech et al., 2003) will differ somewhat from our experimental "hpi". We have indeed seen this small discrepancy in predicted transcriptional age in several of our RNA-seq datasets from trophozoites harvested at 24hpi.

By including additional later time points, one could actually follow the expression profiles over the whole cycle and elucidate if there's an actual transcriptional up-regulation of the genes, or if the + GlcN. parasites show a faster cell cycle progression, with a shifted peak expression timing compared to the - GlcN. parasites.
Response: We did collect a 36hpi time point in the SMC3-3HA-*glmS* and WT strain, with and without glucosamine. We have added this last time point and the WT data from the other two time points to what is now Supp. Fig. 5. Unfortunately, and for reasons unknown, the WT replicates treated with glucosamine showed a significantly advanced "transcriptional age"

compared to the other replicates at 36hpi. Thus, we did not feel comfortable performing the RNA-seq analysis as we did with the other two time points (i.e. subtracting up- and down-regulated genes from the WT control from the SMC3-3HA-*glmS* data sets). We have added this information to the results section (Lines 256 and 261). As the WT parasites treated with glucosamine were approximately 8 hours in advance of the untreated WT parasites for the 36hpi time point, any up- and down-regulated genes might have been due to differences in the cell cycle rather than due to glucosamine treatment. The *glmS* system of inducible knockdown is widely used in *P. falciparum*; however, to our knowledge, no lab has investigated whether glucosamine treatment affects transcription in wildtype cells over the course of the IDC. Thus, for accurate phenotypic characterization of any protein with this system with regard to transcriptomics, we thought it was important to provide an RNA-seq dataset to define the cohort of genes affected by glucosamine treatment in WT parasites. We hope that our study will demonstrate the importance of using stringent controls when using inducible knockdown systems.

"These genes show SMC3 enrichment at their promoter regions at 12 and 24 hpi, but not at 36 hpi (Fig. 4C), and depletion of SMC3 resulted in upregulation at both 12 and 24 hpi (Fig. 4D). Comparison of the SMC3 ChIP-seq data with published Assay for Transposase-Accessible Chromatin using sequencing (ATAC-seq) data (Toenhake et al., 2018) and mRNA dynamics data (Painter et al., 2018) from similar time points in the IDC revealed that SMC3 binding at the promoter regions of these genes inversely correlates with chromatin accessibility (Fig. 4C) and their mRNA levels (Fig. 4E), which both peak in schizont stages. These data are consistent with a role of SMC3 in repressing this gene subset until their appropriate time of expression in the IDC."

The presented correlations certainly make an intriguing point towards the authors conclusion that SMC3/cohesin depletion from the promoter regions of the genes results in a de-repression of these genes and their transcriptional activation. However, the SMC3 knockdown is not complete and only up to 69% as presented on RNA level in these parasites. Therefore a control experiment which needs to be done is to actually show the loss of SMC3 from the presented activated example genes in the knockdown parasites. This could easily be done by ChIP-qPCR or even ChIP-seq, to get a global picture of the actual changes in SMC3 occupation in the knockdown parasites in correlation with changes in transcript levels.
Response: While SMC3-3HA-glmS knockdown is not complete at the RNA level, it is fairly robust at the protein level, especially at 12hpi (Fig. 3A).

"These data suggest that SMC3 knockdown results in a faster progression through the cell cycle or a higher rate of egress/invasion."

The authors could greatly strengthen their conclusions by investigating this thoroughly. Pinpointing the observed phenotype to an actual increase in invasion or egress would add to the authors main conclusion that the loss of SMC3 de-regulates the timing of gene expression for these invasion related genes thereby increasing their transcript levels and thus leading to a

higher rate of egress/invasion.
To determine cell cycle progression simple comparisons between DNA content using a flow cytometer at timepoints together with visual inspection of Giemsa stained blood smears would give a ggod indication towards changes in cell cycle progression. In addition invasion/egress assays by counting newly invaded rings per schizont could reveal, if there are changes in the rate of egress/invasion upon SMC3 knockdown.
Response: We have repeated our growth curve analysis several times and with more clones and have concluded that there is not a significant growth phenotype in SMC3 KD parasites (see what is now Supp. Fig. 4B). We have added images of Giemsa-stained parasites from the knockdown time course we performed to what is now Supp. Fig. 5A. We see no obvious differences in cell morphology caused by glucosamine treatment in the WT or SMC3-3HA-glmS parasites. As we discuss on line 327, very little intact cohesin complex seems to be required for normal growth and mitosis in *S. cerevisiae* and *D. melanogaster*, which is probably why we do not see an obvious growth or morphological phenotype. We believe that SMC3 is probably only a part of a complex controlling transcription of these invasion or egress genes. Thus, the up-regulation of these genes upon SMC3 KD might not be enough to lead to a significant growth or invasion phenotype.

Minor points:

In the MM section on the Cas9 experiments it says dCas9 where it should be Cas9 (line 425)
Response: Thank you for pointing this out. We have corrected this.

It would be great to add which HP1 antibody was used in which dilution in the IFAs to the MM section.
Response: We have added this information to the Materials and Methods section.

In Figure 4C for the gap45 gene there's is some green peak floating around which should not be there.
Response: Thank you for pointing this out, we have corrected it.

Reviewer #3 (Significance (Required)):

Significance:
The manuscript investigates a very timely topic by trying to uncover new molecular mechanisms of transcriptional regulation in P. falciparum. Investigating the role of the cohesin complex/SMC3 in this context provides valuable new insights to the field.
While the first part with the description of the SMC3 cell line and the co-IP experiments largely confirms published data on the existence and composition of the cohesin complex in Plasmodium and its enrichment at the centromeres, the second part is especially intriguing since it investigates the molecular function of SMC3 in more detail. The results pointing to a role of SMC3/cohesin as a transcriptional repressor are of great interest to the field and will open up new concepts for future investigation.

Full Revision

Review COMMONS

Audience:
The work is particularly interesting for people interested in gene regulatory processes in Plasmodium and Apicomplexan parasites in general. At the same time it also nicely points towards shared principles of gene regulation to other eukaryotes in relation to the spatial organization of the genome making the work also very interesting for a broader audience with interest in the general principles of gene regulatory processes in eukaryotic organisms.

Expertise:
P. falciparum epignetics and chromatin biology / gene regulation / Cas9 gene editing

CROSS-CONSULTATION COMMENTS
All reviewers agree that the paper addresses an important topic and provides convincing evidence for enrichment of the cohesin component Smc3 at P. falciparum centromers. In contrast, evidence for a function of Smc3 as a transcriptional repressor of genes in the first part of the parasite life cycle is less well supported. All reviewers agree that the statistical significance of the ChIP experiments needs to be impoved by including biological replicates. In addition, the phenotype of the conditional knock-down should be analysed in more detail by clarifying whether faster cell cycle progression or higher invasion rate are responsible for the observed growth adavantage. Inclusion of transcriptional data from a later time point in addition to the presented data for 12 hpi and 24 hpi was also requested by all reviewers. Finally, several inconsistencies require clarification.

1st Editorial Decision 20th Apr 2023

Dear Dr. Bryant,

Thank you for the submission of your revised manuscript. We have now received the enclosed comments from the referees.

As you will see, while the referees acknowledge that the study has been improved, both referees 1 and 2 point out that the main concern has not been adequately addressed. Given that you indicated that you can address these last comments, I would like to invite you to revise your manuscript one more time. Please co-submit a point-by-point response with your final ms.

In case the outstanding concerns can be satisfactorily addressed, a few editorial requests will also need to be addressed:

- Your ms has 4 main figures but the results and discussion sections are separate. You can either publish your study as a short report with a maximum of 5 figures and combined results and discussion sections, and a maximum of 27.000 characters, or as a full article that needs to have more than 5 figures and separate results and discussion sections. You can find more information about our article types and formats in our guide to authors online.

- Please add up to 5 keywords to the ms file.

- Please correct the conflict of interest subheading to "Disclosure and Competing Interest Statement".

- Please remove the author credits from the ms file. We now use CRediT to specify the contributions of each author in the journal submission system. CRediT replaces the author contribution section. Please use the free text box to provide more detailed descriptions, if you wish. See also guide to authors https://www.embopress.org/page/journal/14693178/authorguide#authorshipguidelines.

- Please correct the REFERENCE FORMAT to Harvard/EMBO reports style. Not more than 10 authors may be listed. Remove the DOIs and rather use issue and page numbers where appropriate.

- A completed author checklist must be submitted with your revised ms. The checklist can be found here: . The completed author checklist will also be part of the transparent peer-review process file.

- The Supplementary figures must be uploaded as EV figures (Figure EV1, etc) and the callouts corrected. We offer a maximum of 5 EV figures. Additional supplementary figures can be included in an Appendix file. Alternatively, all supplementary figures can be moved to the Appendix file. Please see our guide to authors for more information.

- Tables 1-19 should be named Dataset EV#. The legends need to be removed from the word file and added to the excel files. Please also correct all table callouts in the ms text.

- I attach to this email a related ms file with comments by our data editors. Please address all comments in the final ms file.

- The Abstract needs to be shortened. Please describe your findings in the abstract in present tense as per journal policy.

- The ms sections are in the wrong order, please correct.

EMBO press papers are accompanied online by A) a short (1-2 sentences) summary of the findings and their significance, B) 2-3 bullet points highlighting key results and C) a synopsis image that is exactly 550 pixels wide and 200-600 pixels high (the height is variable). You can either show a model or key data in the synopsis image. Please note that text needs to be readable at the final size. Please send us this information along with the final manuscript.

I look forward to seeing a final form of your manuscript as soon as possible. Please use this link to submit your revision: https://embor.msubmit.net/cgi-bin/main.plex

Best regards,
Esther

Esther Schnapp, PhD
Senior Editor
EMBO reports

Referee #1:

In general the revised manuscript addresses most of the major points from my initial review. Especially the addition of another ChIP-seq replicate greatly strengthens this data.

However, concerning the RNA-seq data and the described up-regulation of the invasion genes normally "silenced" by SMC3 I still have some questions concerning the now included 36 hpi time point. While there's no up-regulation of the invasion related genes here as described by the authors, doesn't it strike the authors, that a large part of the genes/proteins described as the invadome in the cited Hu et. al. 2010 paper are actually down regulated in the SMC3 knock down parasites at this time point? So it's basically a completely inverse to the 12 and 24 hpi time points. How does that fit with the observation that there's no accumulation of SMC3 at this time point at these genes in the ChIP-seq? Could the authors comment on this? Because of the described problems with the 36 hpi WT control samples, one could actually consider to repeat the experiments to include a proper WT control. It would be very interesting to see if this down regulation of invasion related genes in the SMC3 knock down is a real difference between the knock down and the control or if this would disappear as a +/- GlcN phenotype (which I doubt).

Finally it is a bit of a pity that the interesting growth phenotype didn't hold up during the additional experiments. Looking at the growth curves, I wonder however, if there's really no difference. The WT and no GlcN cultures display a very steady growth behavior, especially in the 2nd cycle. In contrast the +GlcN cultures show a slightly different growth with a markedly more "stepwise" growth one would expect. In general the replication rate in the 2nd cycle is rather low. Did the authors look into this?

Referee #2:

The authors have responded to many points raised by the reviewers; however, the main criticism (lack of ChIP replicates) has not been adequately addressed. Indeed, the authors have performed a second set of ChIP replicates on another parasite clone, but except for a GO analysis of the overlapping peaks, the second ChIP replicate has not been used in any analysis for backing up the main conclusion of the paper, which is the proposed silencing function of Smc3 in pre-replicative (12 hpi and 24 hpi) parasites. As there is quite a significant difference in the ChIP performance as indicated by the divergent peak numbers, this would be absolutely critical. Without a clear demonstration that the conclusions hold true in a second replicate, I can not recommend the manuscript for publication with EMBO reports in its current form.

Specific comments:

• Line 183: the two replicates showed very different peak numbers, particularly in rings (0,3 x, 2x, 3x in replicate B over replicate A). Given this divergence, it would be critical to provide a more thorough analysis of the associations with gene expression for the second clone. Only one clone has been incorporated into the plots shown in figures 2A and B, and particularly 4A, B, and D, which are the key data on which the conclusion of the manuscript is based. Please provide the corresponding data from the second clone to back up the conclusion.

• Line 187 ff: I could not find any information on how the lack of significant overlap between the ChIP and negative controls was determined. Bedtools intersect? What "time point" was used - there are three? Please provide more details on peak correction.

• Fig. 4: The title states: "SMC3 is involved in transcriptional regulation of genes involved in invasion". The authors propose that Smc3 silences invasion genes in pre-replicative parasites. However, the GO analysis in table 8 indicates that there are actually many more peaks close to invasion related genes at the 36 hpi time point than at the 12 hpi time point. In fact, only 6 of the 67 genes upregulated 12 hpi (table 14) have a called peak at 12 hpi (table 7), whereas 11 genes of the 67 genes upregulated 12 hpi have a peak at 36 hpi, including some invasion genes. So how does the average Smc3 enrichment profile for the 67 genes upregulated at 12 hpi look like at 36 hpi? If Smc3 is indeed involved in their silencing in early stages, then the association should not be evident at 36 hpi.

• Fig. 4: It is not clear to me why the authors chose a gene (gap45) that is actually not significantly upregulated 12 hpi to demonstrate that Smc3 peaks are correlated with silencing 12 hpi. How about msp9, which is the most significantly upregulated invasion gene? Or msp1, which actually has a called peak at 12 hpi?

Minor points:

• Line 205: would be interesting to see whether the 88 common genes (except for the ~24 near centromers) are enriched in exoerythrocytic or sexual genes, i.e. whether they have expression maximum outside of asexual cycle and are commonly silenced by Smc3.

• Line 203 and Figure 2D: numbers don't add up to the ones stated in the text (166, 632, 434).

• Suppl. Fig. 4C: the authors show a time point when Smc3 is virtually not expressed (3 FPKM). Please provide data for the other time points.

• Suppl. Fig. 5: there are only 2 replicates shown for 12hpi - Glcn Smc3 parasites.

• Fig. 4E: The q-value for gap45 at 12 hpi is reported incorrectly from table 18 (0.08 = 8x10-2).

• The link provided for the original ChIP and RNAseq data did not work.

Referee #3:

Within the revised study, the authors widely addressed the referee's concerns. The second biological replicate of the ChIPseq experiment as well as the analysis regarding the negative control strengthen the previous findings. This additional effort improves the quality of the data and the manuscript.
The authors now also consider RNAseq at later time points. Although a correction for glucosamine-treated WT control was difficult, the performed comparisons support the hypothesis regarding cohesin function in transcriptional control.
In summary the authors addressed all concerns and only two minor points remain:
1. The finding that STAG interacts with cohesin is nicely demonstrated in the result section. A short discussion regarding STAG in other eukaryotes would be beneficial to put this finding into context.
2. Figure 3F - numbers of downregulated genes - seems not correctly depicted. As mentioned in the MS:"After filtering for the 12 and 24 hpi time points, 104 and 932 genes were downregulated" However, in Figure 3F it seems that downregulted genes do not add up to 932 genes and might correspond to 12 hpi?

We congratulate the authors for a very well performed study.

Referee 1:

In general the revised manuscript addresses most of the major points from my initial review. Especially the addition of another ChIP-seq replicate greatly strengthens this data.

However, concerning the RNA-seq data and the described up-regulation of the invasion genes normally "silenced" by SMC3 I still have some questions concerning the now included 36 hpi time point. While there's no up-regulation of the invasion related genes here as described by the authors, doesn't it strike the authors, that a large part of the genes/proteins described as the invadome in the cited Hu et. al. 2010 paper are actually down regulated in the SMC3 knock down parasites at this time point? So it's basically a completely inverse to the 12 and 24 hpi time points. How does that fit with the observation that there's no accumulation of SMC3 at this time point at these genes in the ChIP-seq? Could the authors comment on this? Because of the described problems with the 36 hpi WT control samples, one could actually consider to repeat the experiments to include a proper WT control. It would be very interesting to see if this down regulation of invasion related genes in the SMC3 knock down is a real difference between the knock down and the control or if this would disappear as a +/- GlcN phenotype (which I doubt).
As the reviewer suggested, we have repeated the 36 hpi WT control time point and performed RNA-seq. This time, we saw no large difference in visual or transcriptional age (Fig. EV3C and D), so we continued with the differential expression analysis (Dataset EV14) and filtered the corresponding SMC3-HA-glmS data set at 36 hpi (Fig. EV4 and Dataset EV17). We have added the resulting MA plot (Fig. 5B), gene frequency plot (Fig. 5C), and metagene analyses (Fig. 6A and B). Interestingly, the new dataset has confirmed that many invasion-related genes (59 out of the 63 "invasion-related" genes from Dataset EV21) were significantly de-regulated (46 down- and 13 upregulated) in response to glucosamine addition and not necessarily to SMC3 knockdown at this time point. After filtering the SMC3-3HA-glmS 36 hpi dataset with the WT 36 hpi dataset, no egress/invasion-related genes were significantly upregulated, but ten were downregulated specifically in response to SMC3 KD at 36hpi. We do not see SMC3 enrichment at downregulated genes at 36hpi (Fig. 6A, right), so we have no evidence of direct action of SMC3 at these genes at this time point. Our hypothesis is that SMC3 may coordinate invasion-related genes in a spatiotemporal manner, so if these genes are not properly repressed/regulated by SMC3 in early-stage parasites, they may not be able to be properly activated as they normally would be at 36 hpi. We are currently trying to address this hypothesis, but we have no data to support it yet.

With this new 36 hpi time point, we also found that genes enriched for the GO terms "translation" and "ribosome biogenesis" were significantly upregulated in response to SMC3 knockdown at this time point (Dataset EV20). However, when we performed metagene analysis for SMC3 enrichment at upregulated genes (Fig. 6A and B, right) and at translation-related genes specifically, we did not observe specific patterns of enrichment at these genes as we did with SMC3 enrichment at invasion/egress genes at earlier time points. We are not sure why these genes are de-regulated at 36 hpi, but it may be an effect of SMC3 knockdown on chromosome structure or segregation during schizogony. Thus, the correlation of SMC3 enrichment and temporal gene repression is unique to subsets of genes at early time points in the IDC.

Finally it is a bit of a pity that the interesting growth phenotype didn't hold up during the additional experiments. Looking at the growth curves, I wonder however, if there's really no difference. The

WT and no GlcN cultures display a very steady growth behavior, especially in the 2nd cycle. In contrast the +GlcN cultures show a slightly different growth with a markedly more "stepwise" growth one would expect. In general the replication rate in the 2nd cycle is rather low. Did the authors look into this?

We thank the reviewer for pointing out the low replication rate, which might have been due to the quality of blood being used. We have repeated the growth curve analysis and see a replication rate (~5-fold per 48 hr cycle) that one might expect from a normal parasite culture (Fig. 4B). We plotted growth rate (parasitemia at Day X/parasitemia at Day 1) this time rather than parasitemia alone to account for any small, but important differences in parasitemia for the starting cultures of the WT and two SMC3-3HA-glms clones. However, our original conclusion holds true: SMC3 knockdown does not seem to affect parasite growth significantly. On Day 4 only, there seems to be a lag in SMC3-3HA-glmS clone A (+ or – GlcN) compared to clone C (+ or – GlcN) and WT, but this difference resolves at Day 5. If any differences, while not significant, were seen by Days 4 or 5 (which is one cycle after we sampled for RNA-seq), these seem to be due to glucosamine addition in the WT parasites only.

Referee 2:

The authors have responded to many points raised by the reviewers; however, the main criticism (lack of ChIP replicates) has not been adequately addressed. Indeed, the authors have performed a second set of ChIP replicates on another parasite clone, but except for a GO analysis of the overlapping peaks, the second ChIP replicate has not been used in any analysis for backing up the main conclusion of the paper, which is the proposed silencing function of Smc3 in pre-replicative (12 hpi and 24 hpi) parasites. As there is quite a significant difference in the ChIP performance as indicated by the divergent peak numbers, this would be absolutely critical. Without a clear demonstration that the conclusions hold true in a second replicate, I can not recommend the manuscript for publication with EMBO reports in its current form.

Specific comments:

• Line 183: the two replicates showed very different peak numbers, particularly in rings (0,3 x, 2x, 3x in replicate B over replicate A). Given this divergence, it would be critical to provide a more thorough analysis of the associations with gene expression for the second clone. Only one clone has been incorporated into the plots shown in figures 2A and B, and particularly 4A, B, and D, which are the key data on which the conclusion of the manuscript is based. Please provide the corresponding data from the second clone to back up the conclusion.

It is not uncommon for two replicates to show different ChIP-seq peak numbers. Nevertheless, we would like to point out that all the analyses - peak association with genes (Dataset EV7), centromeric and non-centromeric enrichment (Dataset EV5), and convergent versus divergent gene enrichment (Dataset EV6) - were performed with consensus peaks shared between the two replicates. While Figure 2B shows the bedgraphs for ChIP enrichment for replicate A, the bars beneath each track represent consensus peaks shared between replicates A and B. We have added circos plots (Fig. EV2B) and the bedgraph ChIP tracks for replicate B (Fig. EV2C), which show the same pattern of high centromeric

enrichment as replicate A. We found that the quality of the SMC3 ChIP in the 24 hpi sample for clone B was not as high as the other samples for clones A and B, as there is less enrichment over background. We found that all major conclusions we made in the original manuscript are still true, with the exception of SMC3 enrichment decreasing at centromeric regions at 36 hpi. We do not see the same pattern in clone B and have removed this text from the manuscript.

We have now performed the same analysis comparing SMC3 ChIP enrichment in replicate B with differentially expressed genes from the SMC3 knockdown (Fig. EV5A,B). These analyses show the same enrichment of SMC3 in early-stage parasites at genes that are up-regulated upon SMC3 knockdown. Using both SMC3 ChIP-seq replicates, we have performed further metagene analyses in what are now Fig. 6C (clone A) and Fig. EV5C (clone B), which show SMC3 enrichment at the promoters of all invasion-related genes defined in (Hu et. al. 2010) in early, but not late, stage parasites. To provide even further evidence that this pattern of SMC3 enrichment is unique to invasion-related genes, we have performed metagene analysis with the same number of genes that belong to unrelated functional categories. First, we provide a metagene analysis for 62 randomly selected genes that normally reach peak transcription in early-stage parasites (Fig. EV5D). Second, we provide a metagene analysis for 62 genes that reach peak expression in late-stage gametocytes (Fig. EV5E). At both cohorts of genes, very little SMC3 enrichment is seen at any of the time points in the promoter or gene body.

We have also provided the SMC3 ChIP-seq data shown for clone A in former Fig. 4D (now Fig. 6E) for clone B in what is now Fig. EV5F. For clone B, one can also see SMC3 enrichment at *rap2* and *gap45* in early, but not late, stage parasites.

• Line 187 ff: I could not find any information on how the lack of significant overlap between the ChIP and negative controls was determined. Bedtools intersect? What "time point" was used - there are three? Please provide more details on peak correction.
The significant overlap between replicates (or lack thereof between the negative control and SMC3 replicates) was calculated using bedtools fisher. The control ChIP-seq experiment originated from a 12 hpi time point. The lack of a significant overlap at this timepoint was therefore performed with SMC3 from 12hpi. We have added this information in the Materials and Methods section of the manuscript

• Fig. 4: The title states: "SMC3 is involved in transcriptional regulation of genes involved in invasion". The authors propose that Smc3 silences invasion genes in pre-replicative parasites. However, the GO analysis in table 8 indicates that there are actually many more peaks close to invasion related genes at the 36 hpi time point than at the 12 hpi time point. In fact, only 6 of the 67 genes upregulated 12 hpi (table 14) have a called peak at 12 hpi (table 7), whereas 11 genes of the 67 genes upregulated 12 hpi have a peak at 36 hpi, including some invasion genes. So how does the average Smc3 enrichment profile for the 67 genes upregulated at 12 hpi look like at 36 hpi? If Smc3 is indeed involved in their silencing in early stages, then the association should not be evident at 36 hpi.

With this study, we took two approaches to determine SMC3 function in the parasite. First, we performed SMC3 ChIP-seq and looked in an unbiased fashion at which genes were associated with an SMC3 peak. For this, the peak could be located anywhere between 500bp upstream to 500bp downstream of each gene. While this is a valid method, please keep in mind that this includes peaks within the gene body and downstream of the gene, and that the intergenic regions in P. falciparum are rather small, which means it is often difficult to determine which gene a peak actually functionally associates with if it is located in an intergenic region. In addition, while macs peak calling is useful, it may miss important ChIP enrichments that do not fit a "traditional" peak profile, such as broader enrichments.

At the end of the day, the presence or absence of ChIP peaks can provide little information about the function of the chromatin-associated feature without functional analysis, especially (as the reviewer has pointed out) since peaks can be called even in negative control experiments. This is why we took a complementary and functional approach: determine which genes are affected by SMC3 knockdown and then look at SMC3 enrichment at those genes. The enrichment analysis was independent of peak calling. The analysis that the reviewer asks for to look at average SMC3 enrichment at 36hpi of the 67 upregulated genes at 12hpi is actually shown in former Fig. 4B and D (what is now Fig. 6B and E). Indeed, SMC3 is enriched at these gene promoters in early-stage, but not late-stage parasites. We also show this pattern with clone B in Fig. EV5B,F,G.

• Fig. 4: It is not clear to me why the authors chose a gene (gap45) that is actually not significantly upregulated 12 hpi to demonstrate that Smc3 peaks are correlated with silencing 12 hpi. How about msp9, which is the most significantly upregulated invasion gene? Or msp1, which actually has a called peak at 12 hpi?
We chose at random genes that were up-regulated at 12 and 24hpi, and space does not allow us to show the individual ChIP-seq profiles for all genes affected, so we have provided metagene analysis for all "invasion-related" genes (what is now Fig. 6C and EV5C). As requested, we now provide the SMC3 ChIP-seq tracks for both clones A and B at *msp1* and *msp9* in what is now Fig. EV5G. The same pattern of SMC3 enrichment at early stages, but not late, can be seen for these two genes.

Minor points:

• Line 205: would be interesting to see whether the 88 common genes (except for the ~24 near centromers) are enriched in exoerythrocytic or sexual genes, i.e. whether they have expression maximum outside of asexual cycle and are commonly silenced by Smc3.
The majority of the 88 genes that all three time points have in common are located very near to centromeres (please see the screenshot of the genomic view of all 88 genes below from PlasmoDB). As we state in the manuscript, these genes do not seem to be functionally related. GO term enrichment analysis did not yield any categories with a *q* value less than 0.05. We have added metagene analysis of SMC3 enrichment at randomly-chosen genes that reach peak expression in late-stage gametocytes (Fig. EV5E), which does not show the same temporal

pattern of SMC3 binding to gene promoters at any time point during the IDC. Thus, although we do not have ChIP-seq data for sexual stages, we do not think that SMC3 helps to repress exoerythrocytic or sexual stage genes.

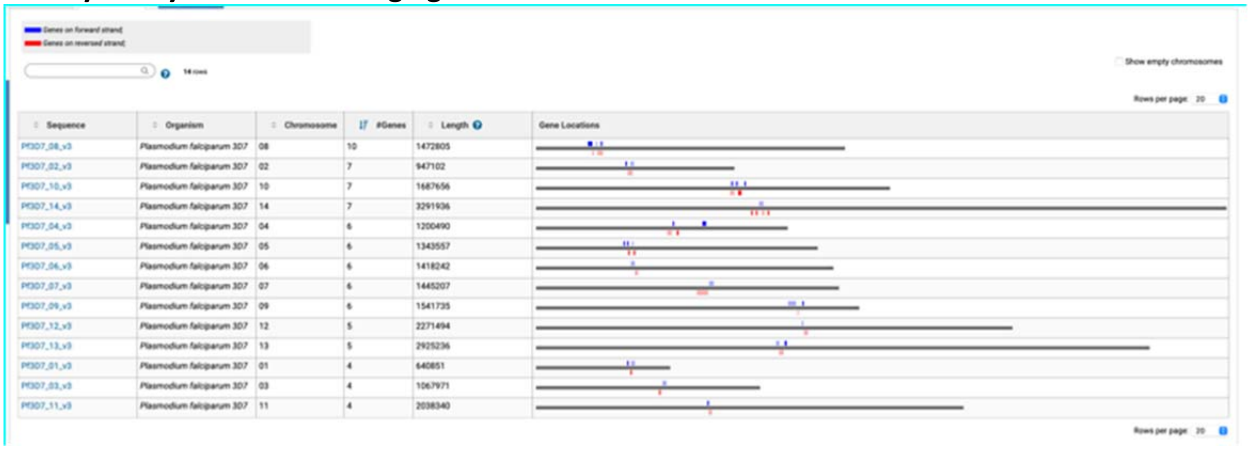

Sequence	Organism	Chromosome	#Genes	Length	Gene Locations
Pf3D7_08_v3	*Plasmodium falciparum 3D7*	08	10	1472805	
Pf3D7_02_v3	*Plasmodium falciparum 3D7*	02	7	947102	
Pf3D7_10_v3	*Plasmodium falciparum 3D7*	10	7	1687656	
Pf3D7_14_v3	*Plasmodium falciparum 3D7*	14	7	3291936	
Pf3D7_04_v3	*Plasmodium falciparum 3D7*	04	6	1200490	
Pf3D7_05_v3	*Plasmodium falciparum 3D7*	05	6	1343557	
Pf3D7_06_v3	*Plasmodium falciparum 3D7*	06	6	1418242	
Pf3D7_07_v3	*Plasmodium falciparum 3D7*	07	6	1445207	
Pf3D7_09_v3	*Plasmodium falciparum 3D7*	09	6	1541735	
Pf3D7_12_v3	*Plasmodium falciparum 3D7*	12	5	2271494	
Pf3D7_13_v3	*Plasmodium falciparum 3D7*	13	5	2925236	
Pf3D7_01_v3	*Plasmodium falciparum 3D7*	01	4	640851	
Pf3D7_03_v3	*Plasmodium falciparum 3D7*	03	4	1067971	
Pf3D7_11_v3	*Plasmodium falciparum 3D7*	11	4	2038340	

- Line 203 and Figure 2D: numbers don't add up to the ones stated in the text (166, 632, 434).

We have re-run these analyses and corrected the numbers in what is now Figure 3D, Dataset EV7, and the text. Some of the totals changed by a few genes due to slight differences in the algorithm, but we are confident in these final figures.

- Suppl. Fig. 4C: the authors show a time point when Smc3 is virtually not expressed (3 FPKM). Please provide data for the other time points.

We have added the data for the additional time points to what is now Fig. EV3B. There is a significant difference in *smc3* transcript levels between untreated WT and SMC3-3HA-*glmS* parasites at 24 and 36 hpi; however, this difference does not seem to translate into a major difference in protein levels, as we show in what is now Fig. EV1D with a western blot of nuclear lysates from untreated trophozoite WT and SMC3-3HA-glmS parasites using an antibody generated in-house against SMC3. It is possible that the mere presence of the *glmS* ribozyme leads to some degradation of the attached transcript, even in the absence of glucosamine. If we were comparing transcript levels between glucosamine-treated WT and SMC3-3HA-glmS parasites, we might see an even more pronounced phenotype than we do see by comparing untreated and glucosamine-treated SMC3-3HA-glmS parasites at 24 hpi. However, to be stringent in our analysis, we only perform differential expression analyses within each strain, not between them.

- Suppl. Fig. 5: there are only 2 replicates shown for 12hpi - Glcn Smc3 parasites.

This is correct, and was stated in the legend for former Figure 3C/D. We were unable to use the third replicate due to low quality of the sequenced DNA library. We have also added text to specify the number of replicates used in the other figure legends concerned, as well as the materials and methods section.

- Fig. 4E: The q-value for gap45 at 12 hpi is reported incorrectly from table 18 (0.08 = 8x10-2).

We thank the reviewer for pointing this out. We have changed it from 8 x 10^-1 to 8 x 10^-2.

• The link provided for the original ChIP and RNAseq data did not work.
We made all data publicly available on April 7th, as requested.

Referee 3:

Within the revised study, the authors widely addressed the referee's concerns. The second biological replicate of the ChIPseq experiment as well as the analysis regarding the negative control strengthen the previous findings. This additional effort improves the quality of the data and the manuscript.
The authors now also consider RNAseq at later time points. Although a correction for glucosamine-treated WT control was difficult, the performed comparisons support the hypothesis regarding cohesin function in transcriptional control.
In summary the authors addressed all concerns and only two minor points remain:
1. The finding that STAG interacts with cohesin is nicely demonstrated in the result section. A short discussion regarding STAG in other eukaryotes would be beneficial to put this finding into context.
We have added this text to the discussion.

2. Figure 3F - numbers of downregulated genes - seems not correctly depicted. As mentioned in the MS:"After filtering for the 12 and 24 hpi time points, 104 and 932 genes were downregulated" However, in Figure 3F it seems that downregulted genes do not add up to 932 genes and might correspond to 12 hpi?
We thank the reviewer for pointing out this discrepancy. We have re-made all the gene frequency plots (now Fig. 5C) for 12, 24, and 36 hpi. For certain genes, there were no data available in Painter et al. dataset used to make these bar graphs, so these genes were somehow grouped together at the 2 hpi time point in the original figure. We have now corrected this in the figure and text. We still find that genes that are upregulated upon SMC3 knockdown at 12 hpi normally reach peak expression in late-stage parasites.

We congratulate the authors for a very well performed study.

1st Revision - Editorial Decision 12th Jul 2023

Dear Jessica,

Thank you for the submission of your revised manuscript. We have now received the enclosed reports from the referees that were asked to assess it, and referee 2 still has one more comment that I would like you to address before we can proceed with the official acceptance of your manuscript.

If you can address referee 1's comment, this would also be very welcome, of course.

I would like to suggest some minor changes to the short summary. Do you agree with :

In the human malaria parasite Plasmodium falciparum, the cohesin subunit SMC3 plays a role in stage-specific gene repression that is distinct from its role in heterochromatin-mediated repression of genes involved in antigenic variation and gametocytogenesis.

The synopsis image is not of sufficient resolution (below 550 pixels wide). Can you please send us a new image as an image file that is exactly 550 pixels wide and between 200 and 600 pixels high? Also, the histone modifications are not readable at the final image size. Please modify.

I would like to suggest some minor changes to the title and abstract. Please let me know whether you agree with the following:

Cohesin contributes to transcriptional repression of stage-specific genes in the human malaria parasite

The complex life cycle of the human malaria parasite, Plasmodium falciparum, is driven by specific transcriptional programs, but it is unclear how most genes are activated or silenced at specific times. There is an association between transcription and spatial organization; however, the molecular mechanisms behind genome organization are unclear. While P. falciparum lacks key genome-organizing proteins found in metazoans, it has all core components of the cohesin complex. To investigate the role of cohesin in P. falciparum, we functionally characterize the cohesin subunit Structural Maintenance of Chromosomes protein 3 (SMC3). SMC3 knockdown during early stages of the intraerythrocytic developmental cycle (IDC) upregulates a subset of genes involved in erythrocyte egress and invasion, which are normally expressed at later stages. ChIP-seq analyses reveal that in the IDC, SMC3 enrichment at the promoter regions of these genes inversely correlates with gene expression and chromatin accessibility. These data suggest that SMC3 binding contributes to repress specific genes until their appropriate time of expression, revealing a new mode of stage-specific gene repression in P. falciparum.

I look forward to seeing a final version of your manuscript as soon as possible. Please use this link to submit your revision: https://embor.msubmit.net/cgi-bin/main.plex

Best regards,
Esther

Esther Schnapp, PhD
Senior Editor
EMBO reports

Referee #1:

The revised manuscript addresses the major points raised by me in the last round of review. The hypothesis raised by the authors "that SMC3 may coordinate invasion-related genes in a spatiotemporal manner, so if these genes are not properly repressed/regulated by SMC3 in early-stage parasites, they may not be able to be properly activated as they normally would be at 36 hpi." is intriguing and it would be very interesting to see some more data in this direction. This could also provide further insights on the molecular mechanisms involved in this regulatory process. However, I 'm well aware that this probably lies out of the scope of this manuscript and might be saved for the next story. So all in all I congratulate the authors to their nice study.

Referee #2:

The authors now have incorporated the ChIP data for the second replicate as suggested, and these seem to largely support the findings of the first replicate of Smc3 enrichment at invasion genes. I have one final suggestion for an additional control to underpin the sepcificity of the Smc3 enrichment in invasion gene promoters in ring stage parasites, which would be to do the metagene analyses shown in Fig. 6 and EV5 with the IgG control ChIP perfromed at 12 hpi.

Referee #1:

The revised manuscript addresses the major points raised by me in the last round of review. The hypothesis raised by the authors "that SMC3 may coordinate invasion-related genes in a spatiotemporal manner, so if these genes are not properly repressed/regulated by SMC3 in early-stage parasites, they may not be able to be properly activated as they normally would be at 36 hpi." is intriguing and it would be very interesting to see some more data in this direction. This could also provide further insights on the molecular mechanisms involved in this regulatory process. However, I 'm well aware that this probably lies out of the scope of this manuscript and might be saved for the next story. So all in all I congratulate the authors to their nice study.

We agree that this would be an interesting direction to take the study. At the moment, we are trying to develop a combination of expansion microscopy and DNA FISH to show that SMC3 or other cohesin components colocalize with invasion-related genes at specific sub-nuclear loci in early stage, but not late-stage parasites. We are also trying to perform HiC in the presence and absence of SMC3 at different stages of the IDC. We hope to get these techniques working soon and elucidate the underlying mechanism more in a future manuscript.

Referee #2:

The authors now have incorporated the ChIP data for the second replicate as suggested, and these seem to largely support the findings of the first replicate of Smc3 enrichment at invasion genes. I have one final suggestion for an additional control to underpin the sepcificity of the Smc3 enrichment in invasion gene promoters in ring stage parasites, which would be to do the metagene analyses shown in Fig. 6 and EV5 with the IgG control ChIP perfromed at 12 hpi.

We have generated metagene plots (just like the ones in Fig. 6 and EV5) with the control anti-HA ChIP that was performed in WT parasites at 12hpi (what are now Figures EV5B & G). These analyses show no control ChIP enrichment at:
- the promoters of genes that are up- or downregulated upon SMC3 knockdown at 12hpi (Fig. EV5B)
- the promoters of invasion-related genes (Fig. EV5G)
- the promoters of randomly chosen genes that normally peak transcription in ring stage parasites (asexual) or gametocytes (sexual) (Fig. EV5G)
We have incorporated these analyses into the main text in the corresponding results section.

Jessica Bryant
Institut Pasteur
Parasites and Insect Vectors
25 rue du Docteur Roux
Biology of Host Parasite Interactions
Paris, France F-75724 Paris Cedex 15
France

Dear Dr. Bryant,

I am very pleased to accept your manuscript for publication in the next available issue of EMBO reports. Thank you for your contribution to our journal.

At the end of this email I include important information about how to proceed. Please ensure that you take the time to read the information and complete and return the necessary forms to allow us to publish your manuscript as quickly as possible.

As part of the EMBO publication's Transparent Editorial Process, EMBO reports publishes online a Review Process File to accompany accepted manuscripts. As you are aware, this File will be published in conjunction with your paper and will include the referee reports, your point-by-point response and all pertinent correspondence relating to the manuscript.

If you do NOT want this File to be published, please inform the editorial office within 2 days, if you have not done so already, otherwise the File will be published by default [contact: emboreports@embo.org]. If you do opt out, the Review Process File link will point to the following statement: "No Review Process File is available with this article, as the authors have chosen not to make the review process public in this case."

Thank you again for your contribution to EMBO reports and congratulations on a successful publication. Please consider us again in the future for your most exciting work.

Yours sincerely,

Esther Schnapp, PhD
Senior Editor
EMBO reports

****

THINGS TO DO NOW:

Please note that you will be contacted by Wiley Author Services to complete licensing and payment information. The required 'Page Charges Authorization Form' is available here: https://www.embopress.org/pb-assets/embo-site/er_apc.pdf - please download and complete the form and return to embopressproduction@wiley.com

EMBO Press participates in many Publish and Read agreements that allow authors to publish Open Access with reduced/no publication charges. Check your eligibility: https://authorservices.wiley.com/author-resources/Journal-Authors/open-access/affiliation-policies-payments/index.html

You will receive proofs by e-mail approximately 2-3 weeks after all relevant files have been sent to our Production Office; you should return your corrections within 2 days of receiving the proofs.

Please inform us if there is likely to be any difficulty in reaching you at the above address at that time. Failure to meet our deadlines may result in a delay of publication, or publication without your corrections.

All further communications concerning your paper should quote reference number EMBOR-2023-57090V3 and be addressed to emboreports@wiley.com.

Should you be planning a Press Release on your article, please get in contact with emboreports@wiley.com as early as possible, in order to coordinate publication and release dates.

EMBO Press Author Checklist

Corresponding Author Name: Jessica M. Bryant	
Journal Submitted to: EMBO Reports	
Manuscript Number: EMBOR-2023-57090V1	

USEFUL LINKS FOR COMPLETING THIS FORM
The EMBO Journal - Author Guidelines
EMBO Reports - Author Guidelines
Molecular Systems Biology - Author Guidelines
EMBO Molecular Medicine - Author Guidelines

Reporting Checklist for Life Science Articles (updated January 2022)

This checklist is adapted from Materials Design Analysis Reporting (MDAR) Checklist for Authors. MDAR establishes a minimum set of requirements in transparent reporting in the life sciences (see Statement of Task: 10.31222/osf.io/9sm4x). Please follow the journal's guidelines in preparing your manuscript.

Please note that a copy of this checklist will be published alongside your article.

Abridged guidelines for figures

1. Data

The data shown in figures should satisfy the following conditions:

- the data were obtained and processed according to the field's best practice and are presented to reflect the results of the experiments in an accurate and unbiased manner.
- ideally, figure panels should include only measurements that are directly comparable to each other and obtained with the same assay.
- plots include clearly labeled error bars for independent experiments and sample sizes. Unless justified, error bars should not be shown for technical replicates.
- if n<5, the individual data points from each experiment should be plotted. Any statistical test employed should be justified.
- Source Data should be included to report the data underlying figures according to the guidelines set out in the authorship guidelines on Data Presentation.

2. Captions

Each figure caption should contain the following information, for each panel where they are relevant:

- a specification of the experimental system investigated (eg cell line, species name).
- the assay(s) and method(s) used to carry out the reported observations and measurements.
- an explicit mention of the biological and chemical entity(ies) that are being measured.
- an explicit mention of the biological and chemical entity(ies) that are altered/varied/perturbed in a controlled manner.
- the exact sample size (n) for each experimental group/condition, given as a number, not a range;
- a description of the sample collection allowing the reader to understand whether the samples represent technical or biological replicates (including how many animals, litters, cultures, etc.).
- a statement of how many times the experiment shown was independently replicated in the laboratory.
- definitions of statistical methods and measures:
  - common tests, such as t-test (please specify whether paired vs. unpaired), simple χ2 tests, Wilcoxon and Mann-Whitney tests, can be unambiguously identified by name only, but more complex techniques should be described in the methods section;
  - are tests one-sided or two-sided?
  - are there adjustments for multiple comparisons?
  - exact statistical test results, e.g., P values = x but not P values < x;
  - definition of 'center values' as median or average;
  - definition of error bars as s.d. or s.e.m.

Please complete ALL of the questions below.
Select "Not Applicable" only when the requested information is not relevant for your study.

Materials

Newly Created Materials	Information included in the manuscript?	In which section is the information available? (Reagents and Tools Table, Materials and Methods, Figures, Data Availability Section)
New materials and reagents need to be available; do any restrictions apply?	Yes	Strains, antibodies, and plasmids are described in the Materials and Methods section and are available upon request. Links to the NextGen sequencing data and proteomics data are given in the Data Availability section.

Antibodies	Information included in the manuscript?	In which section is the information available? (Reagents and Tools Table, Materials and Methods, Figures, Data Availability Section)
For **antibodies** provide the following information:<br>- Commercial antibodies: RRID (if possible) or supplier name, catalogue number and or/clone number<br>- Non-commercial: RRID or citation	Yes	Materials and Methods

DNA and RNA sequences	Information included in the manuscript?	In which section is the information available? (Reagents and Tools Table, Materials and Methods, Figures, Data Availability Section)
**Short novel DNA or RNA including primers, probes**: provide the sequences.	Yes	Materials and Methods and Dataset EV22

Cell materials	Information included in the manuscript?	In which section is the information available? (Reagents and Tools Table, Materials and Methods, Figures, Data Availability Section)
**Cell lines:** Provide species information, strain. Provide accession number in repository **OR** supplier name, catalog number, clone number, and/**OR** RRID.	Not Applicable	
**Primary cultures:** Provide species, strain, sex of origin, genetic modification status.	Not Applicable	
Report if the cell lines were recently **authenticated** (e.g., by STR profiling) and tested for mycoplasma contamination.	Not Applicable	

Experimental animals	Information included in the manuscript?	In which section is the information available? (Reagents and Tools Table, Materials and Methods, Figures, Data Availability Section)
**Laboratory animals or Model organisms:** Provide species, strain, sex, age, genetic modification status. Provide accession number in repository **OR** supplier name, catalog number, clone number, **OR** RRID.	Yes	Materials and Methods
**Animal observed in or captured from the field:** Provide species, sex, and age where possible.	Not Applicable	
Please detail **housing and husbandry conditions**.	Yes	Materials and Methods

Plants and microbes	Information included in the manuscript?	In which section is the information available? (Reagents and Tools Table, Materials and Methods, Figures, Data Availability Section)
**Plants:** provide species and strain, ecotype and cultivar where relevant, unique accession number if available, and source (including location for collected wild specimens).	Not Applicable	
**Microbes:** provide species and strain, unique accession number if available, and source.	Yes	Materials and Methods

Human research participants	Information included in the manuscript?	In which section is the information available? (Reagents and Tools Table, Materials and Methods, Figures, Data Availability Section)
If collected and within the bounds of privacy constraints report on age, sex and gender or ethnicity for all study participants.	Not Applicable	

Core facilities	Information included in the manuscript?	In which section is the information available? (Reagents and Tools Table, Materials and Methods, Figures, Data Availability Section)
If your work benefited from core facilities, was their service mentioned in the acknowledgments section?	Yes	Acknowledgements

Design

Study protocol	Information included in the manuscript?	In which section is the information available? (Reagents and Tools Table, Materials and Methods, Figures, Data Availability Section)
If study protocol has been **pre-registered, provide DOI in the manuscript**. For clinical trials, provide the trial registration number **OR** cite DOI.	Not Applicable	
Report the **clinical trial registration number** (at ClinicalTrials.gov or equivalent), where applicable.	Not Applicable	

Laboratory protocol	Information included in the manuscript?	In which section is the information available? (Reagents and Tools Table, Materials and Methods, Figures, Data Availability Section)
Provide DOI OR other citation details if **external detailed step-by-step protocols** are available.	Not Applicable	

Experimental study design and statistics	Information included in the manuscript?	In which section is the information available? (Reagents and Tools Table, Materials and Methods, Figures, Data Availability Section)
Include a statement about **sample size** estimate even if no statistical methods were used.	Not Applicable	
Were any steps taken to minimize the effects of subjective bias when allocating animals/samples to treatment (e.g. **randomization procedure**)? If yes, have they been described?	Not Applicable	
Include a statement about **blinding** even if no blinding was done.	Not Applicable	
Describe **inclusion/exclusion criteria** if samples or animals were excluded from the analysis. Were the criteria pre-established?<br><br>If sample or data points were omitted from analysis, report if this was due to attrition or intentional exclusion and provide justification.	Yes	Materials and Methods: One technical replicate for one time point of the RNA-seq experiment was dropped due to the low quality of the sequencing data resulting from that library. Standards for quality are pre-established in our pipeline.
For every figure, are **statistical tests** justified as appropriate? Do the data meet the assumptions of the tests (e.g., normal distribution)? Describe any methods used to assess it. Is there an estimate of variation within each group of data? Is the variance similar between the groups that are being statistically compared?	Yes	Figure Legends and/or Materials and Methods

Sample definition and in-laboratory replication	Information included in the manuscript?	In which section is the information available? (Reagents and Tools Table, Materials and Methods, Figures, Data Availability Section)
In the figure legends: state number of times the experiment was **replicated** in laboratory.	Yes	Figure Legends
In the figure legends: define whether data describe **technical or biological replicates**.	Yes	Figure Legends

Ethics

Ethics	Information included in the manuscript?	In which section is the information available? (Reagents and Tools Table, Materials and Methods, Figures, Data Availability Section)
Studies involving **human participants**: State details of **authority granting ethics approval** (IRB or equivalent committee(s), provide reference number for approval.	Not Applicable	
Studies involving **human participants**: Include a statement confirming that **informed consent** was obtained from all subjects and that the experiments conformed to the principles set out in the WMA Declaration of Helsinki and the Department of Health and Human Services Belmont Report.	Not Applicable	
Studies involving **human participants:** For publication of **patient photos**, include a statement confirming that consent to publish was obtained.	Not Applicable	
Studies involving experimental **animals**: State details of **authority granting ethics approva**l (IRB or equivalent committee(s), provide reference number for approval. Include a statement of compliance with ethical regulations.	Yes	Materials and Methods
Studies involving **specimen and field samples:** State if relevant **permits** obtained, provide details of authority approving study; if none were required, explain why.	Not Applicable	

Dual Use Research of Concern (DURC)	Information included in the manuscript?	In which section is the information available? (Reagents and Tools Table, Materials and Methods, Figures, Data Availability Section)
Could your study fall under dual use research restrictions? Please check biosecurity documents and list of **select agents and toxins** (CDC): https://www.selectagents.gov/sat/list.htm	Not Applicable	
If you used a select agent, is the security level of the lab appropriate and reported in the manuscript?	Not Applicable	
If a study is subject to dual use research of concern regulations, is the name of the **authority granting approval and reference number** for the regulatory approval provided in the manuscript?	Not Applicable	

Reporting

The MDAR framework recommends adoption of discipline-specific guidelines, established and endorsed through community initiatives. Journals have their own policy about requiring specific guidelines and recommendations to complement MDAR.

Adherence to community standards	Information included in the manuscript?	In which section is the information available? (Reagents and Tools Table, Materials and Methods, Figures, Data Availability Section)
State if relevant guidelines or checklists (e.g., **ICMJE, MIBBI, ARRIVE, PRISMA**) have been followed or provided.	Not Applicable	
For **tumor marker prognostic studies,** we recommend that you follow the **REMARK** reporting guidelines (see link list at top right). See author guidelines, under 'Reporting Guidelines'. Please confirm you have followed these guidelines.	Not Applicable	
For **phase II and III randomized controlled trials**, please refer to the **CONSORT** flow diagram (see link list at top right) and submit the CONSORT checklist (see link list at top right) with your submission. See author guidelines, under 'Reporting Guidelines'. Please confirm you have submitted this list.	Not Applicable	

Data Availability

Data availability	Information included in the manuscript?	In which section is the information available? (Reagents and Tools Table, Materials and Methods, Figures, Data Availability Section)
Have **primary datasets** been deposited according to the journal's guidelines (see 'Data Deposition' section) and the respective accession numbers provided in the Data Availability Section?	Yes	Data Availability
Were **human clinical and genomic datasets** deposited in a public access-controlled repository in accordance to ethical obligations to the patients and to the applicable consent agreement?	Not Applicable	
Are **computational models** that are central and integral to a study available without restrictions in a machine-readable form? Were the relevant accession numbers or links provided?	Not Applicable	
If publicly available data were reused, provide the respective **data citations in the reference list**.	Yes	Reference List
